# The treehoppers (Hemiptera, Membracidae) of Singapore: An annotated checklist, new records, and an illustrated identification key

**DOI:** 10.3897/zookeys.1295.204613

**Published:** 2026-10-23

**Authors:** Varen Tan, Wei Song Hwang

**Affiliations:** 1 Department of Biological Sciences, National University of Singapore, Block S3 #05-01, 16 Science Drive 4, Singapore 117558, Singapore Department of Biological Sciences, National University of Singapore Singapore Singapore; 2 Lee Kong Chian Natural History Museum, National University of Singapore (NUS), 2 Conservatory Drive, Singapore 117377, Singapore Lee Kong Chian Natural History Museum, National University of Singapore (NUS) Singapore Singapore

**Keywords:** Ant attendants, biodiversity, Centrotinae, host plants, morphology, taxonomy

## Abstract

An updated account of the treehoppers (Hemiptera: Membracidae) of Singapore is presented here. The addition of 16 new records increases the known fauna to 52 species (inclusive of 8 morphospecies) distributed across 11 tribes and 17 genera. An annotated checklist is presented, incorporating old combinations, synonyms, type localities, known distributions, host plant associations, ant attendants, and taxonomic remarks. A visual identification key covering 40 locally occurring species is also provided. Together, this study provides a foundation for future research on Singapore’s membracids and contributes to a broader understanding of the still incompletely documented Oriental fauna.

## Introduction

Treehoppers (Auchenorrhyncha: Membracidae) are phytophagous insects consisting of approximately 3,450 species and 428 genera worldwide (Deitz et al. 2011). They are renowned for their diverse array of ornate pronotal forms, the hypothesised functions of which include crypsis, mimicry, aposematism, pheromone dispersal, and electroreception (Wood 1993; England et al. 2025). Besides the pronotum, other notable traits of these insects include maternal care and ant attendance (Wood 1993). While treehoppers are presently better known from their New World representatives, there exists an abundance of species within the Old World, with the Oriental zoogeographic region being regarded as a centre of diversity of subfamily Centrotinae Amyot & Serville, 1843 (approximately 1,500 spp.) (Wallace and Deitz 2004). In fact, all membracids distributed across Asia are centrotines, the only known exception at present being *Stictocephala bisonia* Kopp & Yonke, 1977 (subfamily Smiliinae Stål, 1866), a species native to the Nearctic, adventive in Europe, and recently discovered in Asia (Yuan et al. 2020). Tribal positions within Centrotinae were thoroughly revised by Wallace and Deitz (2004).

The bulk of Oriental treehopper taxonomy was published during the late 19^th^ to early half of the 20^th^ century. These works were extensively catalogued by Funkhouser (1927b), Metcalf and Wade (1965), and McKamey (1998). Goding was the first to attempt a comprehensive account of Oriental species through his keys to the Old World Membracidae, published in several parts over a span of 20 years (Goding 1931, 1934, 1939, 1949a, 1949b, 1950, 1951). Subsequently, the treehopper fauna of India and China received comprehensive treatment through the works of Ananthasubramanian (1996) and Yuan and Chou (2002) respectively. Taxonomic work on Chinese treehoppers has since been supplemented by Cai and Xu (2004), Zeng (2005), and Li et al. (2019, 2020, 2022a, 2022b). Given that some treehopper species are distributed across the entirety of the Orient (Dmitriev et al. 2022), a better understanding of India’s and China’s treehoppers adds to the documented diversity of the adjacent Southeast Asia.

Funkhouser was a prominent worker on Southeast Asia’s treehoppers, publishing detailed descriptions for various species throughout the region, mainly from Indonesia, Malaysia, and the Philippines. He produced the only proper checklist for treehoppers of a Southeast Asian state with his review of the Philippines Membracidae (Funkhouser 1915). After Funkhouser, work on Southeast Asia’s treehoppers focussed on specific taxa and ecology rather than broad diversity at the country or regional level. Hayashi and Endo (1990) described two new species of *Sipylus* Stål, 1866 from Peninsular Malaysia and Thailand. Stegmann published several works on Malaysian treehoppers, including field observations of *Gigantorhabdus enderleini* Schmidt, 1906 (Stegmann et al. 1998) and other Malaysian taxa (Stegmann and Linsenmair 2002a, 2002b), with a taxonomic revision of *Pyrgauchenia* Breddin, 1901 (Stegmann et al. 2002). Su et al. (2014) utilised molecular tools to uncover a cryptic complex within *Pyrgonota bifoliata* Westwood, 1837, a Philippine endemic, and Lin et al. (2024) further added to our understanding of the species via investigations of their host plants and nymphal morphology. While we now possess a greater understanding of specific taxa, broader information on diversity at the country level remains confined to old and disparate texts. Furthermore, all Southeast Asian states have not had their treehopper fauna broadly re-examined since their original descriptions decades ago. Singapore, an island city-state situated at the tip of the Malay Peninsula, presents a suitable starting point for taxonomic updates.

The Republic of Singapore consists of a central island mass, Pulau Ujong, surrounded by 64 smaller offshore islands which collectively make up 735.7 km^2^ as of December 2024 (Department of Statistics Singapore 2025). While Singapore’s primeval vegetation consisted of lowland dipterocarp forest, mangroves, and freshwater swamp (Yee et al. 2011), British colonisation and the nation’s subsequent independence after 1965 greatly altered the landscape (Corlett 1992). Today, approximately 0.28% of Singapore’s original vegetative cover remains (Yee et al. 2011). Nonetheless, these vestigial habitats paired with networks of newly established parks, secondary forest, and planted streetscapes provide refuges for local insect diversity (Cheong 2011). Treehopper species from Singapore were first collected by Alfred Russel Wallace and described by Walker (1857) (3 spp.). Funkhouser (1918b) subsequently provided the most thorough account of Singapore’s treehoppers to date, noting new species and inventorying several others (24 spp.). Other locality records include those contributed by Buckton (1903) (1 sp.), Matsumura (1912) (1 sp.), Distant (1915, 1916a, 1916b) (3 spp.), Funkhouser (1920, 1935b) (2 spp.), Goding (1939) (1 sp.), and Kato (1940) (1 sp.) (refer to the annotated species checklist for the species recorded by each author). Since then, no further contributions were published, leaving ambiguity over the exact state of Singapore’s current treehopper fauna. To address this knowledge gap, the present study aims to clarify the diversity of treehoppers within Singapore. This is achieved through an annotated species checklist that includes previous combinations, synonyms, type localities, recorded distributions, host plants, and all known ant attendants for locally occurring species. Such an effort not only consolidates the current state of knowledge on many species from scattered texts, but also brings to light new, unpublished records from the Lee Kong Chian Natural History Museum’s Zoological Reference Collection (ZRC), the main specimen source of this study. With an established species checklist, a natural next step is to build a species identification key. Stegmann et al. (2002) cited Goding’s keys to the Old World Membracidae as unsatisfactory, and indeed, we faced difficulty utilising them due to vague terminologies. While alternate keys are available (Funkhouser 1951; Wallace and Deitz 2004), they do not key down to species. Hence, the present study here provides a visual identification key for the updated treehopper species of Singapore.

## Materials and methods

**Figure 1.**
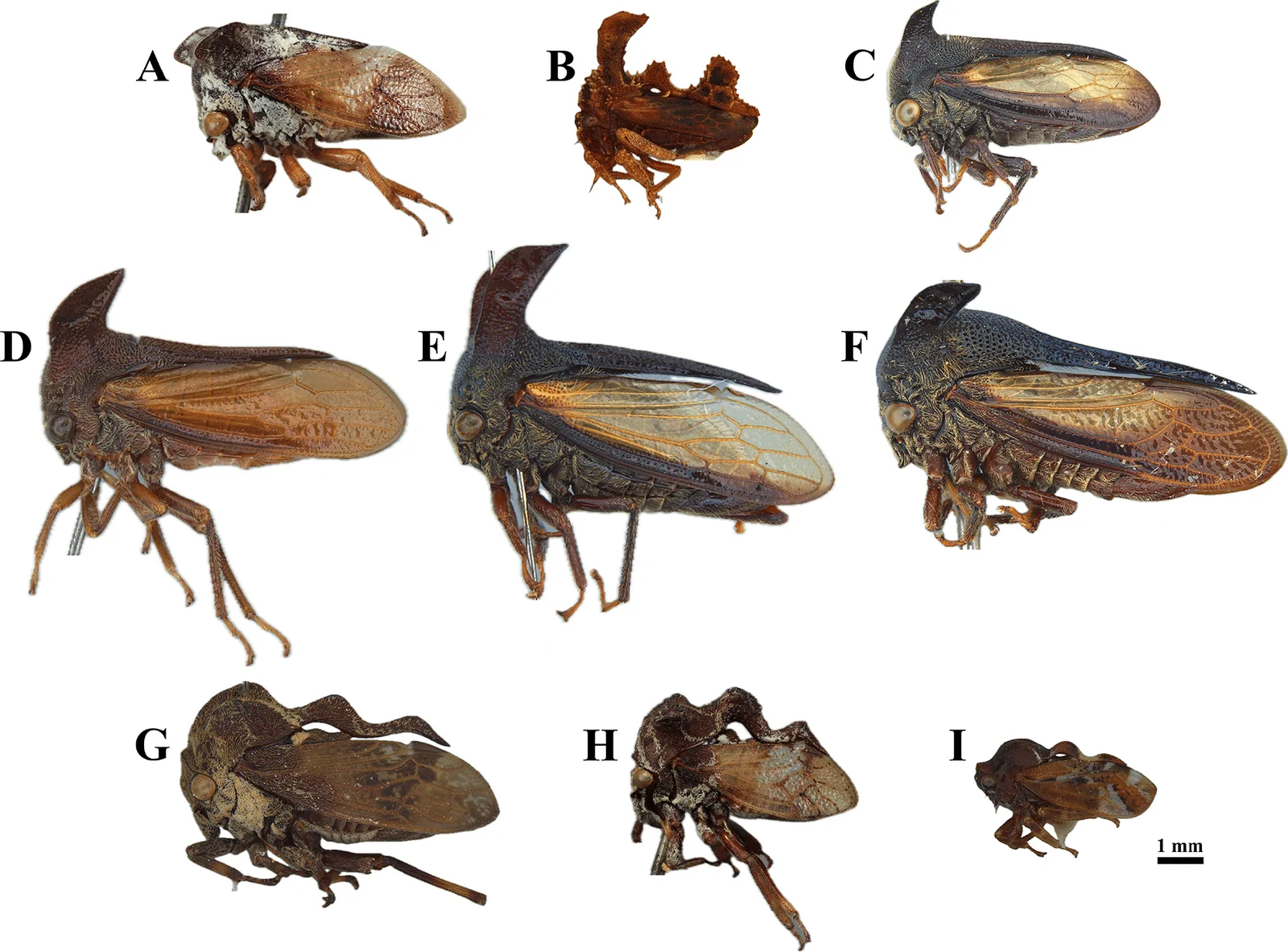
Beaufortianini Wallace & Deitz, 2004: *Centruchus laticornis* Funkhouser, 1918 (**A**). Centrocharesini Goding, 1931: *Centrochares ridleyana* Distant, 1915 (**B**). Centrotypini Haupt, 1929: *Centrotypus belus* (Buckton, 1903) (**C**), *C. laminifer* (Walker, 1857) (**D**), *C.* sp. 1 (**E**), *C.* sp. 2 (**F**). Choucentrini Yuan, 1988: *Evanchon maculatum* Funkhouser, 1937 (**G**), *E. variegatum* (Funkhouser, 1918) (**H**). Ebhuloidesini Goding, 1931: *Ebhul varius* (Walker, 1858) (**I**). All figured specimens are female; (**A**) and (**H**) courtesy of USNM.

**Figure 2.**
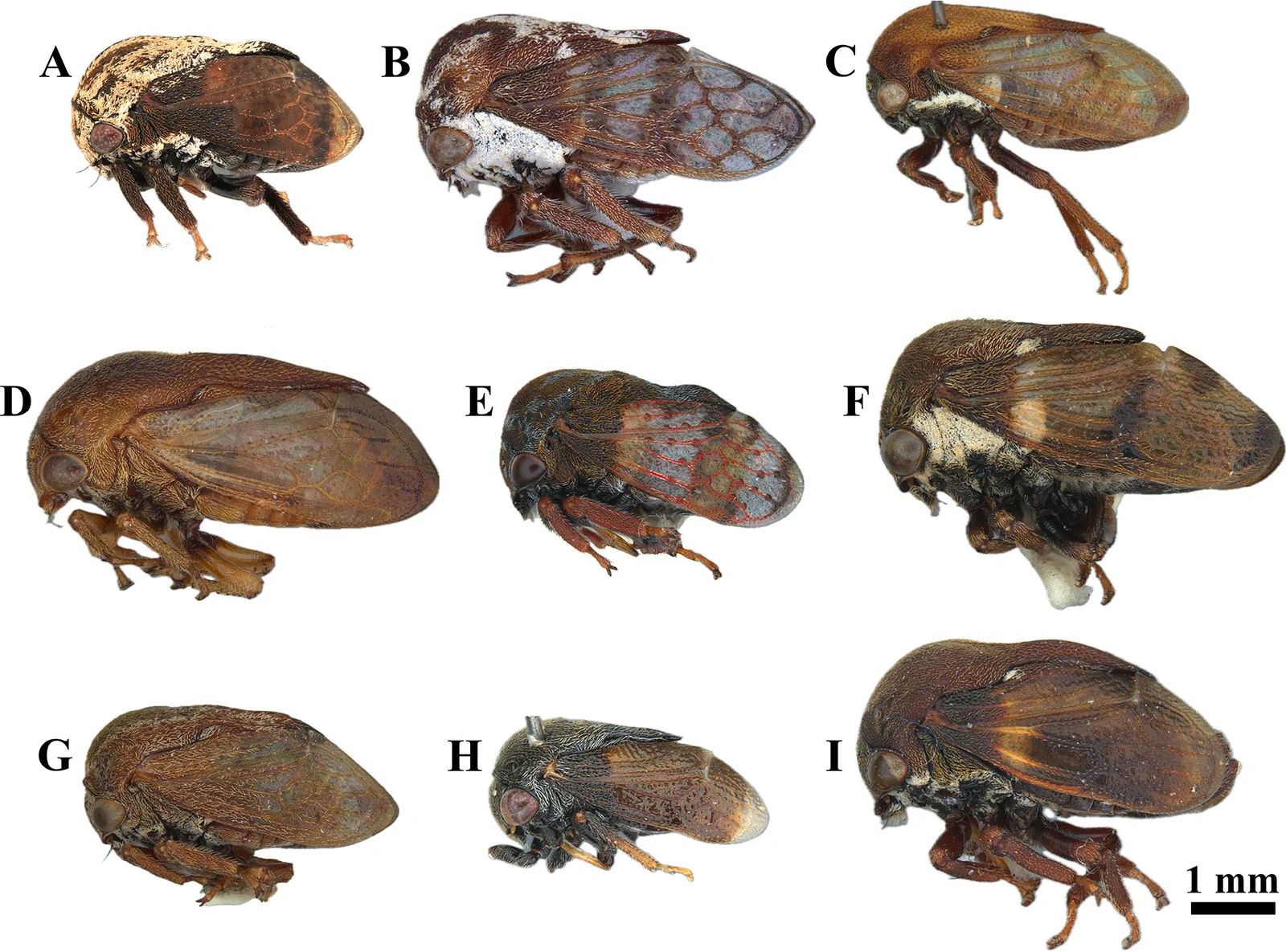
Gargarini Distant, 1908: *Gargara luconica* (Fairmaire, 1846) (**A**), *G. nervosa* Funkhouser, 1918 (**B**), *G. nitidipennis* Funkhouser, 1914 (**C**), *G. projecta* Funkhouser, 1918 (**D**), *G. rubrogranulata* Bierman, 1910 (**E**), *G. sordida* Funkhouser, 1918 (**F**), *G. triangulata* Funkhouser, 1918 (**G**), *G.* sp. 1 (**H**), *G.* sp. 2 (**I**). All figured specimens are female.

**Figure 3.**
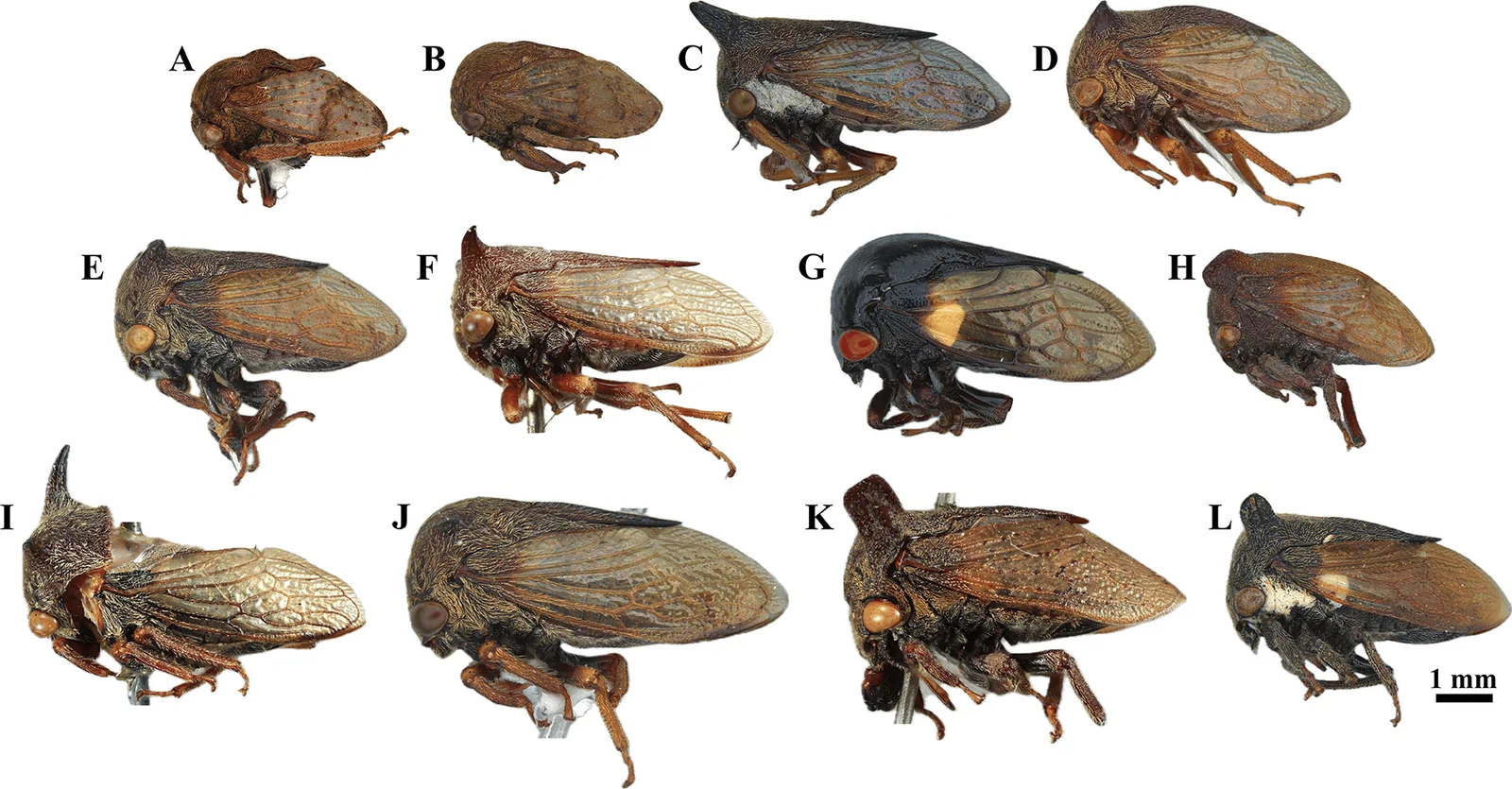
Gargarini Distant, 1908, continued: *Sipylus dilatatus* (Walker, 1851) (**A**); *S.* sp. (**B**); *Tricentrus acuticornis* Funkhouser, 1919 (**C**); *T. assamensis* Distant, 1908 (**D**); *T. banguensis* Funkhouser, 1914 (**E**); *T. brunneus* Funkhouser, 1918 (**F**); *T. fulgidus* Funkhouser, 1919 (**G**); *T. intermedius* (Schmidt, 1911) (**H**); *T. spinicornis* Funkhouser, 1918 (**I**); *T. spinis* Funkhouser, 1935 (**J**); *T. truncaticornis* Funkhouser, 1918 (**K**); *T. cf. albomaculatus* Distant, 1906 (**L**). All figured specimens are female. (**F**), (**I**), and (**K**) courtesy of USNM.

**Figure 4.**
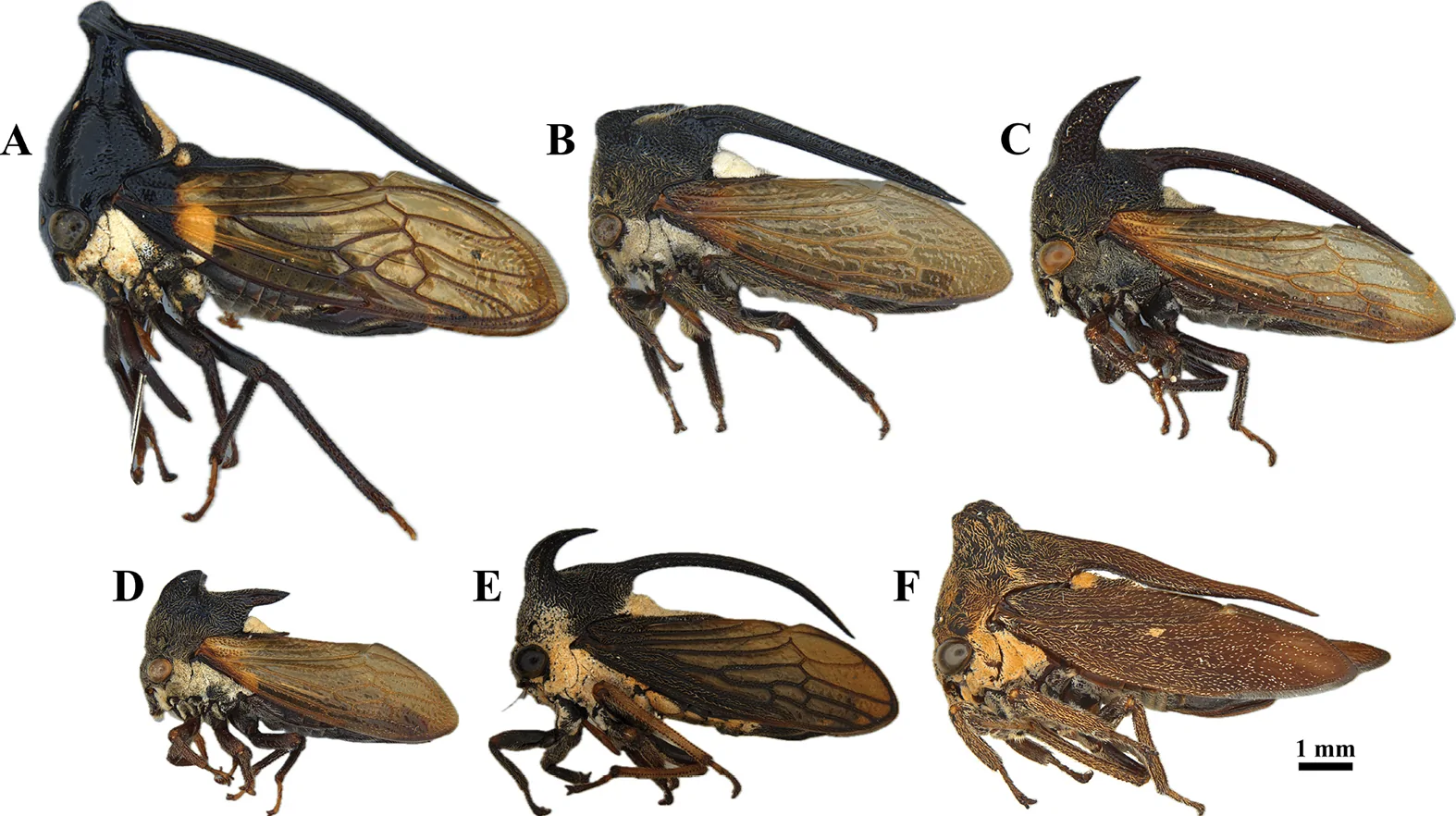
Leptobelini Yuan, 2002: *Leptobelus aff. elevatus* Funkhouser, 1929 (**A**). Leptocentrini Distant, 1908: *Leptocentrus taurus* (Fabricius, 1755) (**B**), *L.* sp. (**C**), *Nilautama minutispina* Funkhouser, 1918 (**D**). Maarbarini Wallace & Deitz, 2004: *Telingana recurvata* Distant, 1916 (**E**). Terentiini Haupt, 1929: *Ceraon orientale* Funkhouser, 1918 (**F**). All figured specimens are female.

**Figure 5.**
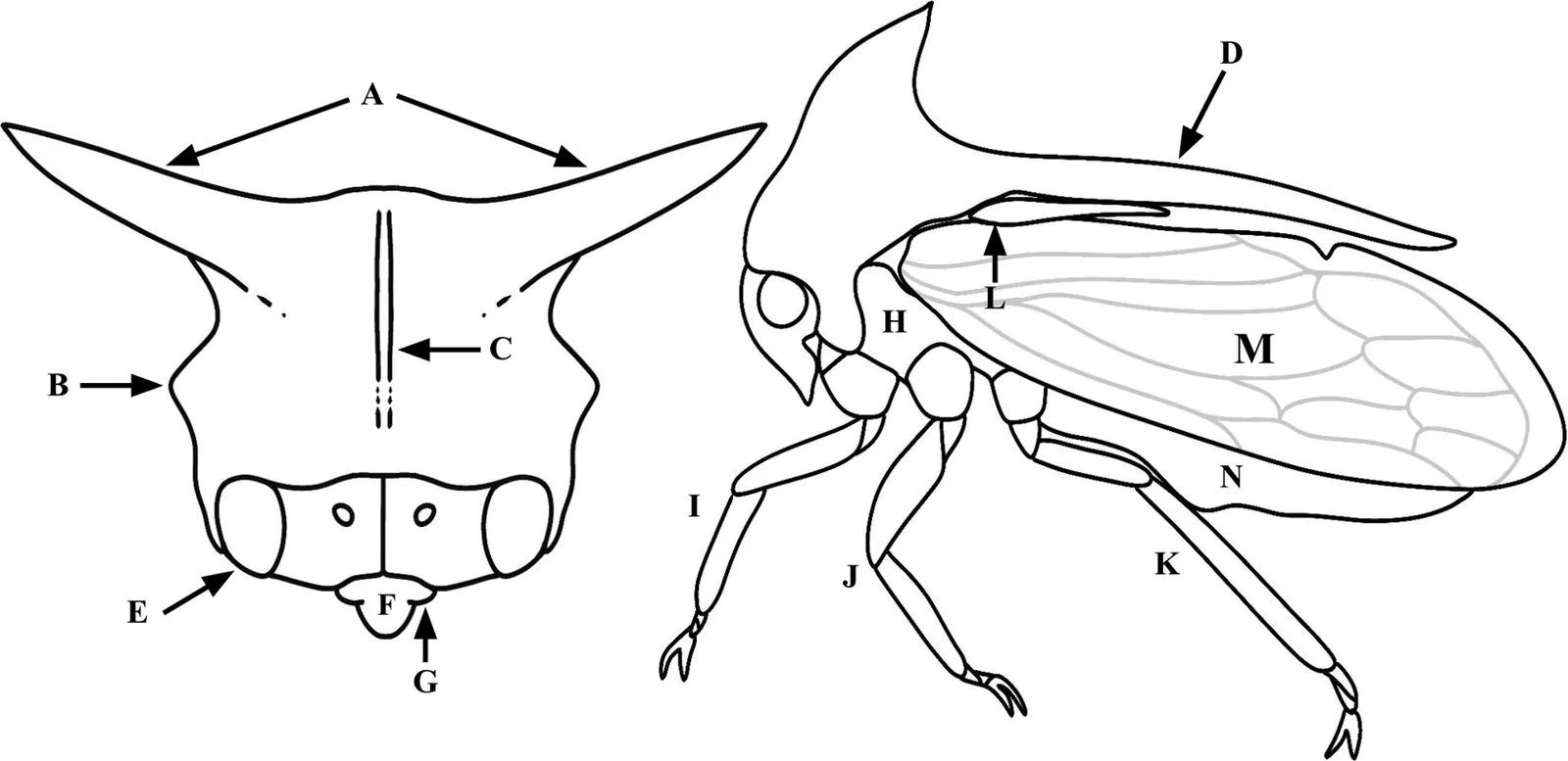
Simplified centrotine external morphology. Pronotum: suprahumerals (**A**), humeral angle (**B**), median carina (**C**), posterior process (**D**). Head: eye (**E**), frontoclypeus (**F**), frontoclypeal lobe (**G**). Thorax: pleuron (note that the metapleuron is concealed by the forewing) (**H**), prothoracic leg (**I**), mesothoracic leg (**J**), metathoracic leg (**K**), scutellum (**L**), forewing (**M**). Abdomen (**N**). Illustrations based on *Centrotypus belus* (Buckton, 1903) ♀.

**Figure 6.**
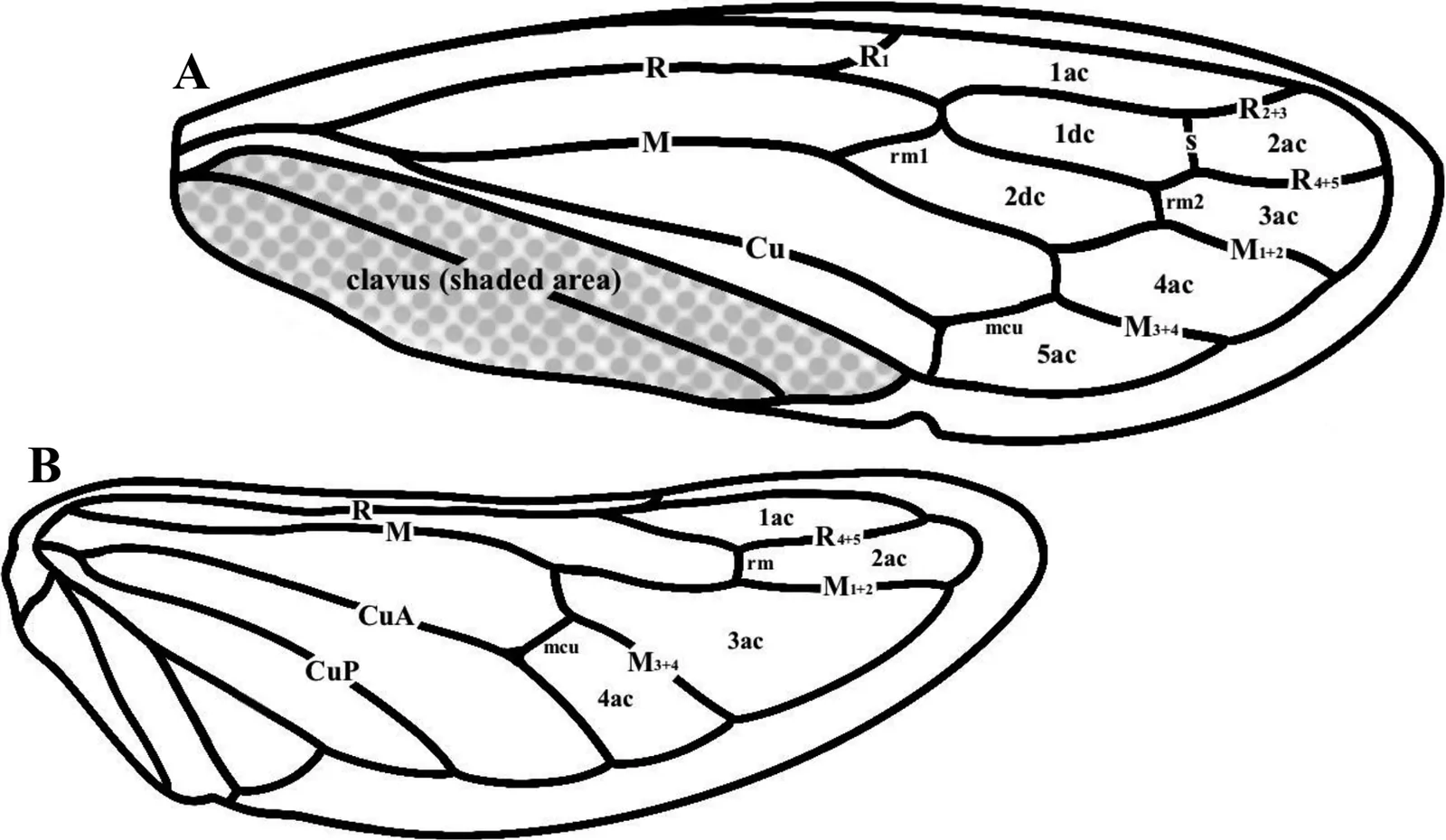
Left forewing (**A**) and left hindwing (**B**). Naming of venation follows Wallace and Deitz (2004) while names of discoidal and apical cells follow Yuan and Chou (2002). Illustrations based on *Centrotypus* sp. 1 ♀.

Subfamily delineations of this study follow Dietrich et al. (2001), while tribal concepts and the naming of general morphological characters follow Wallace and Deitz (2004). The names of forewing cells specifically follow Yuan and Chou (2002). Figures clarifying the basic morphological terms used are provided at the beginning of the visual key (Figs 5, 6). A total of 497 specimens were examined digitally or physically or both, with the majority of the study material (466 specimens) derived from the Lee Kong Chian Natural History Museum’s Zoological Reference Collection (**ZRC**). All ZRC specimens derive from opportunistic collection events and were not obtained through any targeted survey. ZRC specimens were physically examined using a binocular stereo-microscope (Leica M125). A total of 31 specimens from the ZRC (one representative per species for which specimens were available) were photographed using Leica Microsystems CMS GmbH LAS X Version: 3.7.4.23463, and these make up the majority of images in Figs 1, 2, 3, 4 and the visual key. All images were edited using PROCREATE Version 5.4.7. In addition to the ZRC, specimen images from the Smithsonian National Museum of Natural History (**USNM**) (17 specimens, 16 of which are holotypes) and Natural History Museum (**NHMUK**) (14 specimens, two of which are lectotypes) were also examined. All non-types digitally examined from NHMUK were identified by W.D. Funkhouser.

Several ZRC specimens could not be confidently assigned to any named species based on available morphological evidence. These specimens either fell outside the observed intraspecific variation of well-delineated species or could not be assessed against species whose descriptions lacked sufficient diagnostic information. Due to taxonomic issues surrounding several Oriental membracid genera (see discussion section on taxonomic issues), these specimens are retained herein as morphospecies, with their identities to be resolved by future studies.

### Annotated species checklist

Data for the checklist were primarily sourced from literature listed in the catalogues of Funkhouser (1927b), Metcalf and Wade (1965), and McKamey (1998). All membracid species names were standardised according to the World Auchenorrhyncha Database (Dmitriev et al. 2022). Global distributional data presented in the checklist also derive from Dmitriev et al. (2022). Species with type locality outside of Singapore have the authority of their local distribution record indicated. Host plant and ant attendance data are supplemented from ZRC specimen labels and personal observations. All host plant names consolidated from specimen labels and literature were updated according to the World Flora Online Plant List, June 2026 (WFO 2026). Where historical plant names from the literature lacked sufficient information for an updated species-level assignment, they were retained at the genus level.

The true host plant of a treehopper is regarded as the plant on which nymphs develop or female oviposition occur (Lin et al. 2024), while genuine ant attendants consume “honeydew” excreted by adults and nymphs (Wood 1993). However, most literature and museum labels simply list hosts and ant attendants without explicitly acknowledging these phenomena. Hence, all records stated must be treated as preliminary data awaiting future confirmation via targeted field observations for each species. Certain host plant or ant attendant data from labels were subject to interpretation and these are herein clarified. First, labels listing “*Melastoma* sp.” as a host are interpreted to be the commonly occurring *Melastoma malabathricum* given that the only other locally occurring *Melastoma* species, *M. molle*, is locally extinct (Lindsay et al. 2022). Second, labels listing “*Ficus retusa*” as host are treated as referring to *Ficus* sp. due to historical misapplication of the name to either *F. kochummeniana* or *F. microcarpa* (Lindsay et al. 2022). Third, labels listing “*Oecophylla* sp.” as an ant attendant are interpreted to mean *Oecophylla smaragdina* as the species is the sole member of the genus known to occur in Singapore (Wang et al. 2022).

It is noteworthy that most treehopper species display some levels of intraspecific variation. Cases for which known variation are deemed extensive enough to cause potential confusion in species identification is noted within the Remarks section for each species. Species for which the opposite sex is unknown are also explicitly indicated. Some specimens examined (both physically and digitally) have sex indicated as unknown because of the damaged or obscured state of the abdominal venter.

To facilitate ease of reference, a simplified checklist is provided in Table 1, while summaries of host plant and ant attendant data are provided in the Suppl. material 1.

**Table 1.** Simplified checklist of Singapore’s treehoppers. Species with Singapore (SG) as type locality, new species records, new morphospecies records, and species absent from the visual key are also indicated. *Eufrenchia laminifer* is indicated as having uncertain Singapore type locality due to ambiguity surrounding its distribution record (see respective remarks in the annotated species checklist).

**Tribe**	**Species**	**Type locality SG**	**New lit. record**	**New morphospecies record**	**Absent from key**
Beaufortianini Wallace & Deitz, 2004	*Centruchus laticornis* Funkhouser, 1918	X			
Centrocharesini Goding, 1931	*Centrochares horrifica* (Westwood, 1837)				X
	*Centrochares ridleyana* Distant, 1915	X			
Centrotypini Haupt, 1929	*Centrotypus belus* (Buckton, 1903)				
	*Centrotypus laminifer* (Walker, 1857)	X			
	*Centrotypus* sp. 1			X	
	*Centrotypus* sp. 2			X	
Choucentrini Yuan, 1988	*Evanchon maculatum* Funkhouser, 1937		X		
	*Evanchon variegatum* (Funkhouser, 1918)	X			
Ebhuloidesini Goding, 1931	*Ebhul varius* (Walker, 1858)				
Gargarini Distant, 1908	*Cryptaspidia piceola* (Melichar, 1903)				
	*Gargara luconica* (Fairmaire, 1846)		X		
	*Gargara nervosa* Funkhouser, 1918	X			
	*Gargara nitidipennis* Funkhouser, 1914				
	*Gargara projecta* Funkhouser, 1918	X			
	*Gargara rubrogranulata* Bierman, 1910				
	*Gargara semivitrea* (Walker, 1857)	X			X
	*Gargara sordida* Funkhouser, 1918	X			
	*Gargara triangulata* Funkhouser, 1918	X			
	*Gargara* sp. 1			X	
	*Gargara* sp. 2			X	
	*Pantaleon bufo* (Kato, 1928)				
	*Sipylus dilatatus* (Walker, 1851)				
	*Sipylus* sp.			X	
	*Tricentrus acuticornis* Funkhouser, 1919		X		
	*Tricentrus albipes* Funkhouser, 1927				X
	*Tricentrus albomaculatus* Distant, 1908				X
	*Tricentrus assamensis* Distant, 1908				
	*Tricentrus banguensis* Funkhouser, 1914		X		
	*Tricentrus basalis* (Walker, 1851)				X
	*Tricentrus brunneus* Funkhouser, 1918	X			
	*Tricentrus caliginosus* (Walker, 1857)				X
	*Tricentrus congestus* (Walker, 1858)				X
	*Tricentrus fulgidus* Funkhouser, 1919		X		
	*Tricentrus gibbosulus* (Walker, 1858)				X
	*Tricentrus intermedius* (Schmidt, 1911)		X		
	*Tricentrus resectus* Distant, 1916				X
	*Tricentrus spinicornis* Funkhouser, 1918	X			
	*Tricentrus spinis* Funkhouser, 1935				
	*Tricentrus truncaticornis* Funkhouser, 1918	X			
	*Tricentrus cf. albomaculatus* Distant, 1906			X	
Leptobelini Yuan, 2002	*Leptobelus aff. elevatus* Funkhouser, 1929			X	
Leptocentrini Distant, 1908	*Leptocentrus leucaspis* (Walker, 1858)				X
	*Leptocentrus longispinus* Distant, 1908				X
	*Leptocentrus obortus* Distant, 1916				X
	*Leptocentrus taurus* (Fabricius, 1755)				
	*Leptocentrus* sp.			X	
	*Nilautama minutispina* Funkhouser, 1918		X		
Maarbarini Wallace & Deitz, 2004	*Telingana recurvata* Distant, 1916		X		
Micreunini Distant, 1908	*Micreune formidanda* Walker, 1857	X			
Terentiini Haupt, 1929	*Ceraon orientale* Funkhouser, 1918	X			
	*Eufrenchia laminifer* (Buckton, 1903)	?			

### Visual key

The visual key consists mostly of specimen images produced using the imaging protocol described prior. The key is further supplemented by images from other sources, including photographs of eight specimens from the USNM (utilised in steps 3’, 12, 15, 16, 16’, 20, 21, 22, 25, 27, 35, 37’, 39, 40), illustrations of two species from Yuan and Chou (2002: fig. 148A–B, fig. 201D) (utilised in step 20’, 23), and photographs of two specimens from Wallace and Deitz (2004: fig. 16.1B, fig. 22.1O) (utilised in step 2, 8’).

Early taxonomic studies of most membracid species (late 1800s to early 1900s) primarily relied on pronotal characters for species delineation. In contrast, later works have recognised the diagnostic value of additional features, such as the prominence of frontoclypeal lobes, forewing venation, and leg chaetotaxy (particularly the presence of ab- and adlateral cucullate setae) (Ananthasubramanian 1996; Yuan and Chou 2002; Wallace and Deitz 2004). As many species endemic to Southeast Asia were not covered in these treatments, the present key incorporates an updated methodology to delineate Singapore’s treehoppers. The key was also designed to favour a fan-shaped structure, minimising the number of steps required for identification (Packer 2024).

The visual key accounts for sexual dimorphism by ensuring that delineations apply to both female and male members of a species. Sexual differences are generally minor, with males usually being smaller and darker in colouration (sexual dichromatism), but this does not affect the key. Exceptions to this include species for which the sexes differ significantly such that they need to be keyed out in separate steps (e.g., *Tricentrus acuticornis*), or species for which the morphology of either sex is unknown (in cases for which one sex is unknown, the key specifies which sex the couplets apply to). Notably, the key incorporates images of two species (*Cryptaspidia nigris*, *Eufrenchia falcata*) (utilised in step 2, 3’, 21) which do not occur in Singapore. These were used to visualise the diagnostic genus-level characters of locally occurring *Cryptaspidia piceola* and *Eufrenchia laminifer* for which no available specimens could be sourced.

Across all 52 species treated in this study, only 40 are included in the visual key. Twelve species were excluded because their diagnostic characters could not be confidently assessed from the available literature alone, owing to associated taxonomic issues (for more details, see remarks in the annotated species checklist for *Centrochares horrifica*, *Gargara semivitrea*, *Tricentrus albipes*, *T. albomaculatus*, *T. basalis*, *T. caliginosus*, *T. congestus*, *T. gibbosulus*, *T. resectus*, *Leptocentrus leucaspis*, *L. longispinus*, *L. obortus*). Hence, the present key should be regarded as preliminary pending future redescriptions anchored upon type material. Despite these gaps, effort was made to ensure that the key fully accounted for Singapore’s recorded diversity at the genus level following diagnostic characters established by past workers (primarily Wallace and Deitz 2004). Notably, the diagnostic features used to delineate *Centruchus laticornis* and *Ceraon orientale* are the only ones which deviate substantially from Wallace and Deitz (2004) due to the tribal and genus placements of these species likely being erroneous and in need of future revision (refer to the respective remarks for each species in the annotated species checklist).

## Results

### Annotated species checklist

**Membracidae Rafinesque, 1815**

#### Centrotinae Amyot & Serville, 1843

**Beaufortianini Wallace & Deitz, 2004**

***Centruchus* Stål, 1866**

##### *Centruchus laticornis* Funkhouser, 1918

**Type locality**. Singapore (Funkhouser 1918b).

**Recorded distribution**. Malaysia, Singapore.

**Host plant(s)**. Unknown.

**Ant attendant(s)**. Unknown.

**Remarks**. Male unknown. Funkhouser (1918b) placed this species within *Centruchus* on the grounds of its unarmed hind trochanters which he believed was a “sufficient generic character to distinguish the genera *Tricentrus* and *Centruchus* which are in other respects very similar”. However, examination of the holotype suggests that the species instead possesses a closer affinity to tribe Gargarini on account of presence of ab- and adlateral cucullate setae on the mesothoracic femur and frontoclypeal margins converging towards apex. Further investigations are required to ascertain a more accurate tribal and genus placement.

**Material examined (digitally). *Holotype***. Singapore • 1♀; 1917; Baker C.F. leg.; USNMhttp://n2t.net/ark:/65665/34c036ebc-cd15-4722-8c56-5a097e2d5ea4.

#### Centrocharesini Goding, 1931

***Centrochares* Stål, 1866**

##### *Centrochares horrifica* (Westwood, 1837)

*Centrotus horrificus* Westwood, 1837

*Pterygia horrifica*: Walker 1851

*Pterygia postica* Buckton, 1903

*Pterygia spinula* Buckton, 1903

*Centrochares bucktoni* Distant, 1916

*Centrochares posticus* Distant, 1916

**Type locality**. Manila, Luzon, Philippines (Westwood 1837).

**Recorded distribution**. Indonesia, Philippines, Singapore (Funkhouser 1918b).

**Host plant(s)**. Acalypha amentacea var. amentacea (Funkhouser 1919) (Euphorbiaceae).

**Ant attendant(s)**. Unknown.

**Remarks**. Funkhouser noted considerable amounts of morphological variation within *Centrochares horrifica* but repeatedly cited insufficient justification to split the species into separate taxa (Funkhouser 1918a, 1918b, 1919). However, he did not specify what this variation entailed. Further examination of type material is needed to diagnose this species.

**Material examined (digitally). *Non-type specimens***. Philippines – Luzon • 1♀; “Bungyas Laguna”; 1931; P.I. leg.; Brit. Mus. 1934–102.; NHMUK 013387575.

##### *Centrochares ridleyana* Distant, 1915

**Type locality**. Singapore (Distant 1915).

**Recorded distribution**. Malaysia, Singapore.

**Host plant(s)**. *Anacardium* sp. (Anacardiaceae). *Dillenia excelsa* (Dilleniaceae). *Melastoma malabathricum* (Cheong 2011) (Melastomataceae). *Averrhoa carambola* (Oxalidaceae). *Leea indica* (Vitaceae).

**Ant attendant(s)**. *Oecophylla smaragdina*.

**Remarks**. This species was misspelled as “*Centrochares ridleyanum*” by Cheong (2011).

**Material examined (digitally). *Lectotype***. Singapore • 1♀; 1902; Ridley H.N. leg.; NHMUK 015028874.

**Material examined (physically). *Non-type specimens***. Malaysia – Peninsular Malaysia • 2♀♀, 1♂; “Mawai, Johore” [Johor, Mawai]; 10 Nov. 1960; Murphy D.H. leg.; on Melastomum; ZRC ENT00066234 to ENT00066236. Singapore • 1♀, 1 Nymph; 1961; Murphy D.H. leg.; Melastomum; ZRC ENT00066207 to ENT00066208 • 3♀♀, 1♂, 6 Nymph; “Bukit Timah Forest” [Bukit Timah Nature Reserve]; 18 Mar. 1978; Murphy D.H. leg.; Melastomum, Oecophylla; ZRC ENT00065944 to ENT00065945, ENT00066224 to ENT00066231 • 2♀♀; “Bukit Timah Forest edge” [Edge of Bukit Timah Nature Reserve]; 18 Mar. 1978; Murphy D.H. leg.; Melastomum; ZRC ENT00066232 to ENT00066233 • 1♀; 21 Mar. 1978; Murphy D.H. leg.; Anacardium (cashew); ZRC ENT00066031 • 2♀♀, 1♂, 9 Nymph, 1 Unknown sex; “Tyersall Ave” [Near Singapore Botanic Gardens, Tyersall Avenue]; 01 Sep. 1978; Murphy D.H. leg.; Melastomum; ZRC ENT00066209 to ENT00066221 • 1♀; Kent Ridge; 01 Dec. 1991; Tan G.S. leg.; ZRC.6.15806; ZRC ENT00066223 • 1♂; Singapore Botanic Gardens, The Rainforest; 12 Dec. 2024; Yap E.H. leg.; Dillenia excelsa, accompanying female observed egg guarding; ZRC BDP0373647.

#### Centrotypini Haupt, 1929

***Centrotypus* Stål, 1866**

##### *Centrotypus belus* (Buckton, 1903)

*Otinotus belus* Buckton, 1903

*Centrotypus asmodeus* Distant, 1908

**Type locality**. Unknown (Buckton 1903).

**Recorded distribution**. India, Malaysia, Myanmar, Singapore (Funkhouser 1918b).

**Host plant(s)**. *Archidendron* sp. (Fabaceae).

**Ant attendant(s)**. Unknown.

**Remarks**. Males occasionally possess suprahumerals that are produced sub-horizontally as opposed to being obliquely raised in the females.

**Material examined (physically). *Non-type specimens***. Singapore • 1♀; “Bt Timah” [Bukit Timah]; 02 Oct. 1965; Murphy D.H. leg.; ADR; ZRC ENT00066302 • 2♀♀, 1♂, 1 Unknown sex; Nee Soon Swamp Forest; 11 Feb. 1974; Murphy D.H. leg.; ZRC ENT00066296 to ENT00066299 • 1♂; “Fern Valley” [Bukit Timah Nature Reserve, Fern Valley Catchment]; 23 Feb. 1974; Murphy D.H. leg.; Caught by sweeping low foliage; ZRC ENT00066303 • 1♂; “Bukit Timah forest, Tabak Valley” [Bukit Timah Nature Reserve, Taban Valley]; 05 Feb. 1976; Murphy D.H. leg.; ZRC ENT00066301 • 1♀; “Bukit Timah forest, Taban Valley” [Bukit Timah Nature Reserve, Taban Valley]; 02 Jun. 1977; Murphy D.H. leg.; ZRC ENT00066300 • 1♂; Dairy Farm Road; 1.3624°N, 103.7710°E; 18 Oct. 2014; Tan K.T. leg.; ZRC ENT00066304.

##### *Centrotypus laminifer* (Walker, 1857)

*Centrotus laminifer* Walker, 1857

**Type locality**. Singapore (Walker 1857).

**Recorded distribution**. Malaysia, Singapore.

**Host plant(s)**. Unknown.

**Ant attendant(s)**. Unknown.

**Material examined (physically). *Non-type specimens***. Singapore • 1♀; “Island Clulr fvrst” [Forest near Singapore Island Country Club]; 26 Aug. 1983; Murphy D.H. leg.; ZRC ENT00066288.

##### *Centrotypus* sp. 1

**Host plant(s)**. *Uncaria* sp. (Rubiaceae).

**Ant attendant(s)**. Unknown.

**Remarks**. Male unknown.

**Material examined (physically)**. Singapore • 1♀; “Bukit Timah forest” [Bukit Timah Nature Reserve]; 02 Nov. 1973; Murphy D.H. leg.; ZRC ENT00066334 • 3♀♀; “Bukit Timah forest” [Bukit Timah Nature Reserve]; 23 Jan. 1974; Murphy D.H. leg.; Uncaria; ZRC ENT00066327 to ENT00066329 • 3♀♀, 1 Unknown sex; “Bukit Timah forest” [Bukit Timah Nature Reserve]; 27 Jan. 1974; Murphy D.H. leg.; tree trunk; ZRC ENT00066330 to ENT00066333.

##### *Centrotypus* sp. 2

**Host plant(s)**. Unknown.

**Ant attendant(s)**. Unknown.

**Remarks**. Male unknown.

**Material examined (physically)**. Singapore • 1♀; “Murmare Resv.” [Murnane Reservoir]; 08 Apr. 1971; Murphy D.H. leg.; scrub; ZRC ENT00066289.

#### Choucentrini Yuan, 1988

***Evanchon* Goding, 1930**

##### *Evanchon maculatum* Funkhouser, 1937

**Type locality**. Preanger, Java, Indonesia (Funkhouser 1937).

**Recorded distribution**. Indonesia, Singapore (new record).

**Host plant(s)**. *Syzygium grande*, *Syzygium* sp. (Myrtaceae).

**Ant attendant(s)**. *Dolichoderus* sp.

**Remarks**. Male unknown.

**Material examined (digitally). *Holotype***. Indonesia – Java • 1♀; Preanger; May 1936; Funkhouser W.D. leg.; USNMhttp://n2t.net/ark:/65665/3293202c5-9d73-40a0-bb5a-9b4c8d8b3813.

**Material examined (physically). *Non-type specimens***. Singapore • 2♀♀, 1 Unknown sex; “Bukit Timah forest” [Bukit Timah Nature Reserve]; 23 Feb. 1974; Murphy D.H. leg.; on Eugenia. A410-2; ZRC ENT00066197 to ENT00066199 • 2♀♀; “Bukit Timah forest” [Bukit Timah Nature Reserve]; 06 May. 1974; Murphy D.H. leg.; Lower path on Eugenia grandis, Fem v. low path on Eugenia grandis; ZRC ENT00066193 to ENT00066194 • 2♀♀; “Bukit Timah forest” [Bukit Timah Nature Reserve]; 18 Mar. 1978; Murphy D.H. leg.; on Eugenia grandis, Dolichoderus sp.; ZRC ENT00066195 to ENT00066196.

##### *Evanchon variegatum* (Funkhouser, 1918)

*Anchonoides variegatus* Funkhouser, 1918

*Maguva variegata*: Funkhouser 1927b

**Type locality**. Singapore (Funkhouser 1918b).

**Recorded distribution**. Formosa, Indonesia, Malaysia, Singapore.

**Host plant(s)**. Unknown.

**Ant attendant(s)**. Unknown.

**Material examined (digitally). *Holotype***. Singapore • 1♀; 1917; Baker C.F. leg.; USNM 01113146 http://n2t.net/ark:/65665/3aa778891-efeb-4562-807b-63f53a932fb7.

#### Ebhuloidesini Goding, 1931

***Ebhul* Distant, 1907**

##### *Ebhul varius* (Walker, 1858)

*Centrotus varius* Walker, 1858b

*Leptobelus varius*: Stål 1869

**Type locality**. Myanmar (Walker 1858b).

**Recorded distribution**. China, India, Indonesia, Malaysia, Myanmar, New Zealand, Singapore (Funkhouser 1918b).

**Host plant(s)**. *Hydnocarpus alpinus* (Yuan and Chou 2002) (Achariaceae). (*Cananga odorata* (Yuan and Chou 2002) (Annonaceae). *Tamarindus indica* (Yuan and Chou 2002) (Fabaceae). *Vitex pinnata* (Lamiaceae). *Cinnamomum burmanni* (Funkhouser 1927a; Yuan and Chou 2002) (Lauraceae). *Melastoma malabathricum* (Melastomataceae). *Ficus ampelos* (Funkhouser 1927a), *Ficus microcarpa* (Yuan and Chou 2002) (Moraceae). *Syzygium grande*, *Syzygium* sp. (Myrtaceae). *Uncaria rhynchophylla* (Yuan and Chou 2002) (Rubiaceae).

**Ant attendant(s)**. *Dolichoderus thoracicus* (Funkhouser 1927a).

**Material examined (physically). *Non-type specimens***. Singapore • 1♂; “Bukit Timah Forest” [Bukit Timah Nature Reserve]; 20 Feb. 1974; Murphy D.H. leg.; Eugenia; ZRC ENT00066038 • 3♀♀, 2♂♂, 2 Unknown sex; “Bukit Timah Forest” [Bukit Timah Nature Reserve]; 20 Jun. 1974; Murphy D.H. leg.; Eugenia grandis; ZRC ENT00066039 to ENT00066044, ENT00066075 • 1♀, 1♂; “TOE TUCK EST. (BT. TIMAH)” [Toh Tuck]; 18 Apr. 1976; Murphy D.H. leg.; wasteland with orchard and cattleshed; ZRC ENT00066067 to ENT00066068 • 1♀; “Racecourse sec forest “ [Secondary forest near Singapore Turf Club]; 05 Dec. 1976; Murphy D.H. leg.; ZRC ENT00066069 • 4♀♀; “Univ. campus” [National University of Singapore]; 21 Dec. 1977; Murphy D.H. leg.; ZRC ENT00066034 to ENT00066037 • 4♀♀, 1 Nymph, 1 Unknown sex; “Toh Tuck Wasteland” [Toh Tuck wasteland]; 10 Mar. 1978; Murphy D.H. leg.; On Melastomom; ZRC ENT00066045 to ENT00066050 • 3♀♀, 7 Nymph; Linden Drive; 12 Mar. 1978; Murphy D.H. leg.; On Melastomom; ZRC ENT00066051 to ENT00066060 • 3♀♀, 3 Nymph; “Bukit Timah Forest” [Bukit Timah Nature Reserve]; 20 Mar. 1978; Murphy D.H. leg.; On Eugenia infl.; ZRC ENT00066061 to ENT00066066.

#### Gargarini Distant, 1908

***Cryptaspidia* Stål, 1870**

##### *Cryptaspidia piceola* (Melichar, 1903)

*Gargara piceola* Melichar, 1903

**Type locality**. Peradeniya, Sri Lanka (Melichar 1903).

**Recorded distribution**. China, Formosa, India, Japan, Malaysia, Singapore (Funkhouser 1918b), Sri Lanka.

**Host plant(s)**. *Santalum album* (Funkhouser 1933) (Santalaceae).

**Ant attendant(s)**. Unknown.

#### *Gargara* Amyot & Serville, 1843

##### *Gargara luconica* (Fairmaire, 1846)

*Membracis luconica* Fairmaire, 1846

*Enchenopa luconica*: Walker 1851

**Type locality**. Philippines (Fairmaire 1846a).

**Recorded distribution**. Indonesia, Malaysia, Philippines, Singapore (new record).

**Host plant(s)**. *Dendrolobium umbellatum* (Fabaceae). *Sonneratia ovata* (Lythraceae). *Melastoma malabathricum* (Melastomataceae). *Rhodomyrtus tomentosa* (Myrtaceae). *Antidesma bunius*, *Bridelia tomentosa* (Phyllanthaceae). *Phaleria macrocarpa* (Thymelaeaceae).

**Ant attendant(s)**. *Anoplolepis gracilipes*.

**Material examined (digitally). *Non-type specimens***. Indonesia – Sumatra • 1♀; “Fort de Kock, 920M” [Bukittinggi, Fort de Kock]; “920M” [3018 ft]; 1925; Jacobson E. leg.; Brit. Mus. 1926-401.; NHMUK 013387653. Philippines – Luzon • 1♀; “Limay Baraan, Prod” [Limay, Bataan]; 21 Nov. 1924; P.I. leg.; Brit. Mus. 1934–102.; NHMUK 013387654.

**Material examined (physically). *Non-type specimens***. Malaysia – Peninsular Malaysia • 1♀, 1♂; “Batu Caves” [Selangor, Batu Caves]; 21 Feb. 1996; Chen L.M.J. leg.; ZRC ENT00066188, ENT00066592. Singapore • 13♀♀, 9♂♂, 7 Nymph, 2 Unknown sex; “Namly Av.” [Namly Avenue ]; 22 Sep. 1974; Murphy D.H. leg.; Shoots of climber, On Bridelia tomentosa; ZRC ENT00066156 to ENT00066186 • 2♂♂; “Toh Tuck Wasteland” [Toh Tuck wasteland]; 10 Mar. 1978; Murphy D.H. leg.; On Melastomum; ZRC ENT00066590 to ENT00066591 • 3♀♀, 1♂; “Tyersall Istana” [Near Singapore Botanic Gardens, Istana Woodneuk]; 12 Sep. 1978; Murphy D.H. leg.; Melastomum; ZRC ENT00065939, ENT00066153 to ENT00066155 • 2♀♀; “Tyersall Istana” [Near Singapore Botanic Gardens, Istana Woodneuk]; 15 Sep. 1978; Murphy D.H. leg.; ZRC ENT00065942 to ENT00065943 • 2♀♀; “Changi Mangrove “ [Mangrove at Changi]; 06 Aug. 1979; Murphy D.H. leg.; Sonneratia Ovoh; ZRC ENT00066588 to ENT00066589 • 1♀; Temasek Hall Blk C; 1.2927°N, 103.7705°E; 15 Oct. 2015; Lee N. leg.; ZRC ENT00066187 • 2♀♀; “SBG nr NOG” [Singapore Botanic Gardens, near National Orchid Garden]; 24 Nov. 2022; Yap E.H. leg.; Bridelia tomentosa; ZRC BDP0371771 to BDP0371772 • 2♀♀, 1♂; “SBG nr Raffles Building” [Singapore Botanic Gardens, near Raffles Building]; 03 Aug. 2023; Yap E.H. leg.; Rhodomyrtus tomentosa; ZRC BDP0372434 to BDP0372436.

##### *Gargara nervosa* Funkhouser, 1918

**Type locality**. Singapore (Funkhouser 1918b).

**Recorded distribution**. Indonesia, Malaysia, Singapore.

**Host plant(s)**. *Cinnamomum* sp. (Lauraceae).

**Ant attendant(s)**. Unknown.

**Remarks**. Male unknown.

**Material examined (digitally). *Holotype***. Singapore • 1♀; 1917; Baker C.F. leg.; USNMhttp://n2t.net/ark:/65665/35b6367af-2842-4a32-a723-fa9a1ce18526.

**Material examined (physically). *Non-type specimens***. Singapore • 1♀; “Namly Ave kpg” [Namly Avenue Kampung]; 02 Mar. 1974; Murphy D.H. leg.; Cinnamomum sp.; ZRC ENT00066593.

##### *Gargara nitidipennis* Funkhouser, 1914

**Type locality**. Los Baños, Luzon, Philippines (Funkhouser 1914b).

**Recorded distribution**. China, Formosa, Indonesia, Malaysia, Philippines, Singapore (Funkhouser 1918b).

**Host plant(s)**. *Hypolytrum nemorum*, *Scleria* sp. (Cyperaceae). *Melastoma malabathricum* (Melastomataceae).

**Ant attendant(s)**. *Anoplolepis gracilipes*.

**Remarks**. Funkhouser (1915, 1919, 1920) repeatedly noted this species to vary widely in size. This species is found primarily on the inflorescences of its Cyperaceous hosts.

**Material examined (digitally). *Holotype***. Philippines – Luzon • 1♀; “Los Banos” [Los Baños]; 1914; Baker P.I. leg.; USNMhttp://n2t.net/ark:/65665/3bc503fde-652b-4142-adf5-f35f573b86c9.

**Material examined (physically). *Non-type specimens***. Malaysia – Peninsular Malaysia • 1♀; “Penang, 1/2 way House” [Penang halfway house]; Oct. 1960; Murphy D.H. leg.; swept 1500; ZRC ENT00066141 • 1♂, 1 Unknown sex; “Mawai, Johore” [Mawai, Johor]; 10 Nov. 1960; Murphy D.H. leg.; Melastomum; ZRC ENT00066142, ENT00066144. Singapore • 1♂; “Bukit Timah vill. aore leand” [Bukit Timah]; 21 Mar. 1976; Murphy D.H. leg.; ZRC ENT00066137 • 6♀♀, 1♂, 1 Unknown sex; “Toh Tuck rd belukar” [Toh Tuck]; 05 Mar. 1978; Murphy D.H. leg.; Sclera, fls. of Sclera grass; ZRC ENT00066129 to ENT00066136 • 1♀; “BUKIT TIMAH, RIFLE RANGE ROAD” [Rifle Range Road]; 29 Apr. 1989; Yeo K.L. leg.; ZRC ENT00066139 • 1♂; Nee Soon Swamp Forest; 09 May 1992; Ong V. leg.; ZRC ENT00066138.

##### *Gargara projecta* Funkhouser, 1918

**Type locality**. Singapore (Funkhouser 1918b).

**Recorded distribution**. Indonesia, Malaysia, Singapore.

**Host plant(s)**. *Cinnamomum iners* (Lauraceae). *Melastoma malabathricum* (Melastomataceae). *Syzygium grande* (Myrtaceae). *Aquilaria malaccensis* (Thymelaeaceae).

**Ant attendant(s)**. Unknown.

**Material examined (digitally). *Holotype***. Singapore • 1♀; 1917; Baker C.F. leg.; USNM USNM01113020 http://n2t.net/ark:/65665/331ae2ff0-23d9-46d7-af0f-8926caf5b5f5.

**Material examined (physically). *Non-type specimens***. Malaysia – Peninsular Malaysia • 1♀; Kuala Lumpur, Templar Park; 1963; Pippet J.R. leg.; ZRC ENT00066029. Singapore • 1♀; “University Ground Spot e PHL” [National University of Singapore]; Jun. 1965; Murphy D.H. leg.; ZRC ENT00066120 • 1♂; “Sime Road Forest Clearance” [Forest clearance at Simon Road]; 02 Dec. 1976; Murphy D.H. leg.; ZRC ENT00066239 • 1♀; “TOH TUCK EST. (BT. TIMAH)” [Toh Tuck]; 05 Dec. 1976; Murphy D.H. leg.; Wasteland with orchard and cattleshed; ZRC ENT00066124 • 1♀; “Racecourse forest” [Singapore Turf Club]; 01 Sep. 1977; Murphy D.H. leg.; ZRC ENT00066123 • 8♀♀, 1 Nymph; “Toh Tuck Wasteland” [Toh Tuck wasteland]; 10 Mar. 1978; Murphy D.H. leg.; Melastomom; ZRC ENT00066099 to ENT00066107 • 2♀♀; “Tyersall Ave” [Near Singapore Botanic Gardens, Tyersall Avenue]; 09 Aug. 1978; Murphy D.H. leg.; Melastomom; ZRC ENT00066108 to ENT00066109 • 6♀♀, 1♂; “Tyersall Istana” [Near Singapore Botanic Gardens, Tyersall Avenue]; 12 Sep. 1978; Murphy D.H. leg.; Melastomom; ZRC ENT00066145 to ENT00066150, ENT00066237 • 1♀, 1♂; “Tyersall Istana” [Near Singapore Botanic Gardens, Tyersall Avenue]; 15 Sep. 1978; Murphy D.H. leg.; ZRC ENT00066110, ENT00066238 • 2♀♀; “Simi. Rd forest” [Forest at Simon Road]; 08 Nov. 1979; Murphy D.H. leg.; ZRC ENT00066121 to ENT00066122 • 1♀; “BUKIT TIMAH, RIFLE RANGE ROAD” [Rifle Range Road]; 29 Apr. 1989; Yeo K.L. leg.; ZRC ENT00066140 • 1♀; “Kent Ridge “ [Kent Ridge]; 23 Aug. 1990; K.N. leg.; ZRC ENT00066125 • 1♀; “NUS, Kent Ridge” [National University of Singapore]; 18 Jul. 1991; Chan S.H. leg.; ZRC ENT00066126 • 1♀; “NS113 Upper Pierce Reservoir Nr. Plot #30and31” [Upper Pierce Reservoir]; 17 Jun. 1993; Lim Y.H. leg.; ZRC ENT00066143 • 1♀; Bukit Panjang; 29 Dec. 2012; Lua H.K. leg.; ZRC ENT00066151 • 1♂; Upper Seletar Reservoir; 1.3991°N, 103.8052°E; 22 Jan. 2024; Yap E.H. leg.; Cinnamomum iners; ZRC BDP0372546 • 2♀♀; Singapore Botanic Gardens, Lawn E; 08 Jun. 2023; Yap E.H. leg.; Aquilaria malaccensis; ZRC BDP0372083 to BDP0372084.

##### *Gargara rubrogranulata* Bierman, 1910

**Type locality**. Semarang, Java, Indonesia (Bierman 1910).

**Recorded distribution**. Indonesia, Malaysia, Singapore (Funkhouser 1918b).

**Host plant(s)**. *Butea monosperma* (Funkhouser 1935a) (Fabaceae). *Melastoma malabathricum*, *Memecylon caeruleum*, *Memecylon* sp. (Melastomataceae). *Ficus* sp. (Moraceae). *Syzygium grande*, *Syzygium* sp. (Myrtaceae). *Citrus* sp. (Rutaceae).

**Ant attendant(s)**. Unknown.

**Material examined (physically). *Non-type specimens***. Malaysia – Peninsular Malaysia • 1♀; “Mauai Johore” [Mawai, Johor]; 10 Nov. 1960; Murphy D.H. leg.; Melasto..(?); ZRC ENT00065996. Singapore • 2♀♀, 2♂♂; “Fern Valley” [Bukit Timah Nature Reserve, Fern Valley Catchment]; 23 Feb. 1973; Murphy D.H. leg.; On Memec3lon; ZRC ENT00065946 to ENT00065949 • 1♀; Nee Soon Swamp Forest; 11 Feb. 1974; Murphy D.H. leg.; ZRC ENT00065950 • 2♀♀, 4♂♂, 1 Nymph; Fort Canning; 16 Mar. 1978; Murphy D.H. leg.; On Ficus retusa; ZRC ENT00065951, ENT00065952 to ENT00065956, ENT00066030 • 1 Nymph; “Bukit Timah Forest” [Bukit Timah Nature Reserve]; 20 Mar. 1978; Murphy D.H. leg.; On Eugenia infl; ZRC ENT00065957 • 1♀, 1♂, 4 Nymph; “Mt. Faber” [Mount Faber]; 21 Mar. 1978; Murphy D.H. leg.; On Melastomum; ZRC ENT00065958 to ENT00065963 • 1 Nymph; “Tyersall Ave” [Near Singapore Botanic Gardens, Tyersall Avenue]; 01 Sep. 1978; Murphy D.H. leg.; Melastomum; ZRC ENT00066222 • 2♀♀, 3 Nymph, 1 Unknown sex; “Tyersall Avenue” [Near Singapore Botanic Gardens, Tyersall Avenue]; 08 Sep. 1978; Murphy D.H. leg.; On Melastomum; ZRC ENT00065964 to ENT00065969 • 1♀; Kent Ridge; 20 Jan. 1992; Tan C. leg.; ZRC.6.15810; ZRC ENT00065970 • 1♂; Nee Soon Swamp Forest; 28 May 1992; Yeo K.L. leg.; Light trap 2130–2330; ZRC ENT00065971 • 5♀♀, 17♂♂; “NS 20 Lower Pierce Forest” [Lower Pierce Forest]; 04 Jun. 1992; Yeo K.L. leg.; Fogging; ZRC ENT00065973 to ENT00065994 • 1♀; “Bt. Timah Greenbank Park” [Greenbank Park]; 13 Oct. 1995; Tan H.H. leg.; ZRC ENT00065972 • 1♂; “SIN, Kampong Mandai Kechil” [Kampong Mandai Kechil]; 11 Jul. 1996; Goh Y.Y. leg.; Cilrus sp.; ZRC ENT00065995 • 2♀♀; Bukit Batok; 06 Feb. 2024; Yap E.H. leg.; on Syzygium sp.; ZRC BDP0372583 to BDP0372584.

##### *Gargara semivitrea* (Walker, 1857)

*Centrotus semivitreus* Walker, 1857

**Type locality**. Singapore (Walker 1857).

**Recorded distribution**. Malaysia, Singapore.

**Host plant(s)**. Unknown.

**Ant attendant(s)**. Unknown.

**Remarks**. The sole description of this species (Walker 1857) is brief. Besides black coloration and a horizontal posterior process slightly shorter than the abdomen, other features are vaguely worded. Examination of type(s) is needed to ascertain the diagnostic traits of this species.

##### *Gargara sordida* Funkhouser, 1918

**Type locality**. Singapore (Funkhouser 1918b).

**Recorded distribution**. Formosa, Indonesia, Malaysia, Singapore.

**Host plant(s)**. *Dalbergia latifolia* (Funkhouser 1935a) (Fabaceae). *Ayenia maingayi* (Malvaceae). *Bridelia tomentosa* (Phyllanthaceae).

**Ant attendant(s)**. Unknown.

**Material examined (digitally). *Holotype***. Singapore • 1♀; 1917; Baker C.F. leg.; USNM USNM01113087 http://n2t.net/ark:/65665/3f7833c2d-926a-4d5d-a360-007c15ba4ed6.

**Material examined (physically). *Non-type specimens***. Singapore • 1♀; Central Catchment Nature Reserve; 1.3525°N, 103.8242°E; 24 Jan. 2024; Yap E.H. leg.; on Byttneria maingayi; ZRC BDP0372557 • 1♀; Chestnut Nature Park; 1.3711°N, 103.7815°E; 27 Feb. 2024; Tan V. leg.; on Bridelia tomentosa; ZRC BDP0372626.

##### *Gargara triangulata* Funkhouser, 1918

**Type locality**. Singapore (Funkhouser 1918b).

**Recorded distribution**. Indonesia, Malaysia, Singapore.

**Host plant(s)**. *Dendrolobium umbellatum* (Fabaceae). *Vitex pinnata* (Funkhouser 1935a) (Lamiaceae).

**Ant attendant(s)**. Unknown.

**Remarks**. Male unknown.

**Material examined (digitally). *Holotype***. Singapore • 1♀; 1917; Baker C.F. leg.; USNM 01113123 http://n2t.net/ark:/65665/34cb10c97-84d4-42e1-bde9-71952a365624.

**Material examined (physically). *Non-type specimens***. Singapore • 2♀♀; “Canberra, near Canberra MRT station” [Near Canberra MRT station]; 27 Mar. 2022; Yap E.H. leg.; on Dendrolobium umbellatum; ZRC ENT00066491 to ENT00066492 • 1♀, 1♂; Toa Payoh; 1.3395°N, 103.8522°E; 22 Jan. 2024; Yap E.H. leg.; on Dendrolobium umbellatum; ZRC BDP0372550 to BDP0372551.

##### *Gargara* sp. 1

**Host plant(s)**. *Uncaria* sp. (Rubiaceae).

**Ant attendant(s)**. Unknown.

**Material examined (physically)**. Singapore • 1♀, 2♂♂, 2 Nymph; “Island Club forest” [Forest near The Singapore Island Country Club]; 10 Mar. 1992; Murphy D.H. leg.; on Uncaria sp.; ZRC ENT00066594 to ENT00066598.

##### *Gargara* sp. 2

**Host plant(s)**. *Calophyllum inophyllum* (Calophyllaceae). *Cinnamomum iners* (Lauraceae).

**Ant attendant(s)**. *Oecophylla smaragdina*.

**Remarks**. Male unknown.

**Material examined (physically)**. Singapore • 1♀, 1♂; “Punggol Way near Valour Primary School” [Punggol Way, near Valour Primary School]; 17 Apr. 2022; Yap E.H. leg.; on Calophyllum inophyllum; ZRC ENT00066493, ENT00066895.

#### *Pantaleon* Distant, 1916

##### *Pantaleon bufo* (Kato, 1928)

*Eupantaleon bufo* Kato, 1928

*Antialcidas bufo*: Kato 1940

**Type locality**. Kaohsiung, Formosa (Kato 1928).

**Recorded distribution**. China, Formosa, Singapore (Kato 1940).

**Host plant(s)**. Unknown.

**Ant attendant(s)**. Unknown.

**Remarks**. Male unknown.

#### *Sipylus* Stål, 1866

##### *Sipylus dilatatus* (Walker, 1851)

*Centrotus dilatatus* Walker, 1851

*Sipylus nodipennis* Funkhouser, 1914

**Type locality**. Philippines (Walker 1851).

**Recorded distribution**. Indonesia, Malaysia, Philippines, Singapore (Funkhouser 1918b).

**Host plant(s)**. Acalypha amentacea var. amentacea (Funkhouser 1919) (Euphorbiaceae). *Streblus asper* (Moraceae). *Phyllanthus reticulatus* (Funkhouser 1919) (Phyllanthaceae).

**Ant attendant(s)**. Unknown.

**Remarks**. Funkhouser (1919) noted this species to be solitary and without ant attendants.

**Material examined (digitally). *Non-type specimens***. Philippines – Luzon • 1♀; “Los Banos” [Los Baños]; 1914; Baker P.I. leg.; 208. Holotype of Sipylus nodipennis Funkhouser, 1914; USNMhttp://n2t.net/ark:/65665/3c1a4704f-c7d2-419c-9f4b-1cf75d9930eb.

**Material examined (physically). *Non-type specimens***. Singapore • 1♀; “Sima Rd. fwt” [Forest at Simon Road]; 05 May. 1974; Murphy D.H. leg.; ZRC ENT00065941.

##### *Sipylus* sp.

**Host plant(s)**. *Melastoma malabathricum* (Melastomataceae).

**Ant attendant(s)**. Unknown.

**Material examined (physically)**. Malaysia – Peninsular Malaysia • 1♀; “Penang Hill: 1500” [Penang Hill]; 25 Oct. 1960; Murphy D.H. leg.; University of Malaysia collection. 1500; ZRC ENT00066098 • 2♀♀; “Mawai Johore” [Johor, Mawai]; 10 Nov. 1960; Murphy D.H. leg.; On melastomom; ZRC ENT00066096 to ENT00066097. Singapore • 16♀♀, 3♂♂; “Toh Tuck Wasteland” [Toh Tuck wasteland]; 10 Mar. 1978; Murphy D.H. leg.; On melastomom; ZRC ENT00066076 to ENT00066091, ENT00066190 to ENT00066192 • 1♂; “Tyersall Istana” [Near Singapore Botanic Gardens, Istana Woodneuk]; 12 Sep. 1978; Murphy D.H. leg.; On melastomom; ZRC ENT00065940 • 1♀; Nee Soon Swamp Forest; 31 Dec. 1988; Koo Y.H. leg.; ZRC ENT00066094 • 1♀, 1♂; “Sinu Rd Forest” [Simon Road]; 03 May 1992; Murphy D.H. leg.; On melastomom; ZRC ENT00066092 to ENT00066093 • 1♀; Holland Woods; 1.3383°N, 103.7813°E; 18 Oct. 2016; Tan A. leg.; ZRC ENT00066095.

#### *Tricentrus* Stål, 1866

##### *Tricentrus acuticornis* Funkhouser, 1919

**Type locality**. Culasi, Visayas, Philippines (Funkhouser 1919).

**Recorded distribution**. China, Indonesia, Philippines, Singapore (new record).

**Host plant(s)**. *Bauhinia* sp., *Dendrolobium umbellatum* (Fabaceae). *Melastoma malabathricum* (Melastomataceae).

**Ant attendant(s)**. Unknown.

**Remarks**. ZRC specimens possess suprahumerals which are subequal in length to the distance between their bases, as opposed to longer than the length between their bases as in Funkhouser’s holotype. However, due to a lack of any other distinct morphological differences, ZRC specimens are herein identified as *Tricentrus acuticornis*, with difference in suprahumeral length interpreted as intraspecific variation (suprahumeral length is known to vary intraspecifically within other congeners, for example, see remarks for *T. brunneus*).

Yuan and Chou (2002) were the first to record the male morphology of this species, noting its peculiar lack of suprahumerals compared to the females. No males thus far have been collected from Singapore.

**Material examined (digitally). *Holotype***. Philippines – Visayas • 1♀; “Panay, Culasi” [Antique, Culasi]; 27 May 1918; McGregor R.C. leg.; USNMhttp://n2t.net/ark:/65665/38ad49951-08d7-4b32-bc5c-2406ad24a04b. ***Non-type specimens***. Malaysia – Peninsular Malaysia • 3♀♀; Kuala Lumpur; 24 Mar. 1926; Pendlebury H.M. leg.; on Bauhinia sp.; NHMUK 013387596 to 013387597, 013387599 • 1♀; Kuala Lumpur; 29 Mar. 1926; Pendlebury H.M. leg.; NHMUK 013387598• 1♀; “Malaya”; 1929; Corbett G.H. leg.; NHMUK 013387600.

**Material examined (physically). *Non-type specimens***. Singapore • 1♀; “Tyersall Istana” [Near Singapore Botanic Gardens, Istana Woodneuk]; 12 Sep. 1978; Murphy D.H. leg.; On melastomom; ZRC ENT00066527 • 1♀; Seletar; 08 Feb. 1989; Lea Y.S. leg.; ZRC ENT00066528 • 1♀; “SBG, Coach Park nr Tyersall Gate” [Singapore Botanic Gardens]; 08 Mar. 2021; Yap E.H. leg.; Dendrolobium umbellatum; ZRC ENT00062959.

##### *Tricentrus albipes* Funkhouser, 1927

**Type locality**. Fort de Kock, Sumatra, Indonesia (Funkhouser 1927a).

**Recorded distribution**. Indonesia, Singapore (Goding 1939).

**Host plant(s)**. Unknown.

**Ant attendant(s)**. Unknown.

**Remarks**. Male unknown. Described based on a single specimen, Funkhouser (1927a) noted this species to be near *Tricentrus brunneus* but smaller, with suprahumerals at a different angle, and with tibiae and tarsi white. These differences, while notable, are difficult to interpret without examination of physical specimens.

##### *Tricentrus albomaculatus* Distant, 1908

**Type locality**. Myitta, Tenasserim, Myanmar (Broomfield 1971).

**Recorded distribution**. Bangladesh, Brazil, China, Formosa, Hawaii, India, Indonesia, Malaysia, Myanmar, Pakistan, Singapore (Funkhouser 1918b).

**Host plant(s)**. *Cananga odorata* (Zimmerman 1948) (Annonaceae). *Xanthium strumarium* (Ahmad and Yasmeen 1978) (Asteraceae). *Cajanus cajan*, *Senna bicapsularis*, *Sesbania* sp. (Zimmerman 1948), *Dalbergia sissoo* (Ahmad and Yasmeen 1978) (Fabaceae). *Eucalyptus robusta* (Yuan and Chou 2002), *Eucalyptus* sp. (Kershaw 1913; Zimmerman 1948) (Myrtaceae). *Datura metel* (Ananthasubramanian 1996), *Withania somnifera* (Ahmad and Yasmeen 1978) (Solanaceae). *Phyla nodiflora* (Ahmad and Yasmeen 1978) (Verbenaceae).

**Ant attendant(s)**. *Pheidole megacephala* (Kershaw 1913).

**Remarks**. Ahmad and Yasmeen (1978) reported the lectotype of *Tricentrus albomaculatus* to be male, while Ananthasubramanian (1996) noted the lectotype to be female. Notably, Distant (1908) and (Ahmad and Yasmeen 1978) note the metapleuron of *T. albomaculatus* to possess a white tomentose patch visible through the forewing while the pro- and mesopleuron remains black. Ananthasubramanian (1996) and Yuan and Chou (2002) make no such distinction, referring to the entire pleuron as white tomentose. A re-assessment of type(s) is needed to clarify these ambiguities.

Swezey (1912) reported *T. albomaculatus* to be an invasive species of O’ahu, Hawaii, with Kershaw (1913) dating the first record of its occurrence to approximately 1902. The presence of this species in Hawaii was subsequently corroborated by Funkhouser (1938) who identified specimens of *T. albomaculatus* from China as identical to Hawaiian material.

##### *Tricentrus assamensis* Distant, 1908

**Type locality**. Margherita, India (Distant 1908).

**Recorded distribution**. China, India, Indonesia, Malaysia, Singapore (Funkhouser 1918b).

**Host plant(s)**. *Cinnamomum iners* (Lauraceae). *Melastoma malabathricum* (Melastomataceae). *Ficus* sp. (Moraceae). *Santalum album* (Funkhouser 1933; Ananthasubramanian 1996) (Santalaceae).

**Ant attendant(s)**. *Oecophylla smaragdina*.

**Material examined (physically). *Non-type specimens***. Singapore • 1♀, 2♂♂; Fort Canning; 09 Mar. 1978; Murphy D.H. leg.; ZRC ENT00066263, ENT00066272, ENT00066274 • 2♀♀; “Botanic Gardens” [Singapore Botanic Gardens]; 14 Mar. 1978; Murphy D.H. leg.; ZRC ENT00066259 to ENT00066260 • 5♀♀; Fort Canning; 16 Mar. 1978; Murphy D.H. leg.; Ficus retusa; ZRC ENT00066267 to ENT00066271 • 4♀♀, 2♂♂; “Univ. Campus” [National University of Singapore]; 20 Aug. 1978; Murphy D.H. leg.; “Cinnamomum imo4s” [Cinnamomum iners], Oecophylla sp.; ZRC ENT00066282 to ENT00066287 • 1♀; “Tyersall Istana” [Near Singapore Botanic Gardens, Istana Woodneuk]; 12 Sep. 1978; Murphy D.H. leg.; On melastomom; ZRC ENT00066248 • 1♀, 1♂; Kent Ridge; 23 Apr. 1979; Murphy D.H. leg.; ZRC ENT00066261 to ENT00066262 • 2♀♀; “BUKIT TIMAH CAMPUS” [Bukit Timah]; 03 Feb. 1981; Koo Y.H. leg.; ZRC ENT00066275 to ENT00066276 • 1♀; Kent Ridge; 03 Dec. 1991; Guha B. leg.; ZRC ENT00066277 • 1 Unknown sex; “View Rd” [View Road]; 02 Nov. 2013; Ho Y.K. leg.; ZRC ENT00066278.

##### *Tricentrus banguensis* Funkhouser, 1914

**Type locality**. Banggi Island, Malaysia (Funkhouser 1914a).

**Recorded distribution**. India, Indonesia, Malaysia, Singapore (new record).

**Host plant(s)**. *Melastoma malabathricum* (Melastomataceae). *Bridelia tomentosa* (Phyllanthaceae).

**Ant attendant(s)**. Unknown.

**Material examined (digitally). *Holotype***. Malaysia – East Malaysia • 1♀; “Banguey Island” [Banggi Island]; USNMhttp://n2t.net/ark:/65665/338bb2b68-cb17-4170-9a5f-e1b6f9febc47.

**Material examined (physically). *Non-type specimens***. Singapore • 1♀; “Namly Ave. kpg “ [Namly Avenue Kampung]; 02 Mar. 1974; Murphy D.H. leg.; Legum tree foliage; ZRC ENT00066548 • 1♀; 22 Sep. 1974; Murphy D.H. leg.; Bridelia tomentosa; ZRC ENT00066273 • 1♀; “Linden Drive Kampong” [Linden Drive]; 27 Mar. 1975; Murphy D.H. leg.; ZRC ENT00066549 • 1♀; “Bukit Timah Vill. ware l__nd” [Bukit Timah, village wasteland ]; 21 Mar. 1976; Murphy D.H. leg.; ZRC ENT00066550 • 1♀; “Univ. Campus” [National University of Singapore]; 24 Dec. 1976; Murphy D.H. leg.; ZRC ENT00066547 • 4♀♀; “Toh Tuck Wasteland” [Toh Tuck wasteland]; 10 Mar. 1978; Murphy D.H. leg.; Melastoma; ZRC ENT00066545 to ENT00066546, ENT00066551 to ENT00066552 • 1♀; “Tyersall Ave” [Singapore Botanic Gardens]; 09 Aug. 1978; Murphy D.H. leg.; Melastoma; ZRC ENT00066306 • 3♀♀, 3♂♂, 1 Nymph; “Tyersall Istana” [Near Singapore Botanic Gardens, Istana Woodneuk]; 12 Sep. 1978; Murphy D.H. leg.; Melastoma; ZRC ENT00066249 to ENT00066255 • 3♀♀; “Tyersall Istana” [Near Singapore Botanic Gardens, Istana Woodneuk]; 15 Sep. 1978; Murphy D.H. leg.; ZRC ENT00066256 to ENT00066258.

##### *Tricentrus basalis* (Walker, 1851)

*Centrotus basalis* Walker, 1851

**Type locality**. Hong Kong, China (Walker 1851).

**Recorded distribution**. Brunei, China, Formosa, Hong Kong, India, Indonesia, Malaysia, Singapore (Matsumura 1912), Sri Lanka.

**Host plant(s)**. *Humulus scandens* (Yuan and Chou 2002) (Cannabaceae).

**Ant attendant(s)**. Unknown.

**Remarks**. Available descriptions of *Tricentrus basalis* (Walker 1851; Yuan and Chou 2002) are generally concordant, but the characters reported are commonplace within the genus and cannot be considered diagnostic. Funkhouser (1938) noted this species as challenging to determine due to males possessing very short and depressed suprahumerals compared to the females which possessed suprahumerals which were more erect, longer, and variable in length. Yuan and Chou (2002) similarly observed shorter suprahumerals in males. Examination of type(s) will be a useful first step towards resolving the identity of this species.

##### *Tricentrus brunneus* Funkhouser, 1918

**Type locality**. Singapore (Funkhouser 1918b).

**Recorded distribution**. China, Indonesia, Malaysia, Singapore, Vietnam.

**Host plant(s)**. *Elaeagnus pungens* (Yuan and Chou 2002) (Elaeagnaceae). *Robinia pseudoacacia* (Yuan and Chou 2002) (Fabaceae). *Broussonetia papyrifera* (Yuan and Chou 2002) (Moraceae).

**Ant attendant(s)**. Unknown.

**Remarks**. *Tricentrus brunneus* as described by Yuan and Chou (2002) possesses white tomentosity on the pleuron, but this is absent in the original species description by Funkhouser (1918b), and examination of the holotype indicates that the pleuron is instead clothed in long golden hairs. Funkhouser (1920) noted a tendency in this species to vary in suprahumeral length.

**Material examined (digitally). *Holotype***. Singapore • 1♀; 1917; Baker C.F. leg.; USNMhttp://n2t.net/ark:/65665/3e73da3f3-079e-40ca-82f2-49d069f6e0da.

##### *Tricentrus caliginosus* (Walker, 1857)

*Centrotus caliginosus* Walker, 1857

**Type locality**. Malacca, Malaysia (Walker 1857).

**Recorded distribution**. Indonesia, Malaysia, Singapore (Distant 1916b), Thailand.

**Host plant(s)**. Unknown.

**Ant attendant(s)**. Unknown.

**Remarks**. The sole description of *Tricentrus caliginosus* (Walker 1857) is brief and provides little basis for distinguishing it from its congeners. An examination of type(s) will be useful in clarifying its identity.

##### *Tricentrus congestus* (Walker, 1858)

*Centrotus congestus* Walker, 1858a

*Otaris congestus*: Goding 1934

**Type locality**. India (Walker 1858a).

**Recorded distribution**. China, India, Indonesia, Malaysia, Myanmar, Philippines, Singapore (Funkhouser 1920).

**Host plant(s)**. *Cyanthillium cinereum* (Ananthasubramanian 1996) (Asteraceae).

**Ant attendant(s)**. Unknown.

**Remarks**. Jacobi (1944) noted the best distinguishing feature of *Tricentrus congestus* to be a dark-brown stripe at the transition of the costal margin to the apical margin, a trait acknowledged by Walker (1858a) but omitted by Distant (1908). Curiously, Ananthasubramanian (1996) did not mention this feature despite examining type material of *T. congestus*. Distant (1908) and Goding (1934) further noted a tendency of this species to exhibit additional transverse veins on the forewing, but Distant (1908) remarked that this trait was neither constant nor uniform. Other characters reported for this species are of little use in distinguishing it from its congeners.

Males of this species are darker with humeral angles less prominent and posterior process straight instead of slightly arched (Ananthasubramanian 1996).

##### *Tricentrus fulgidus* Funkhouser, 1919

**Type locality**. Pontianak, Kalimantan, Indonesia (Funkhouser 1929).

**Recorded distribution**. China, Indonesia, Malaysia, Singapore (new record), Vietnam.

**Host plant(s)**. *Ficus benjamina*, *Ficus hispida*, *Ficus lacor*, *Ficus religiosa* (Yuan and Chou 2002), *Ficus* sp. (Moraceae).

**Ant attendant(s)**. Unknown.

**Remarks**. Yuan et al. (1986) noted the eyes of this species to vary in colouration ranging from different hues of red, brown, and black. All ZRC specimens were only observed to possess red eyes. This species seems to display a high degree of host specificity to *Ficus* spp., with records from China (Yuan and Chou 2002) and Singapore consistently indicating the genus as an exclusive host.

**Material examined (digitally). *Holotype***. Indonesia – Borneo • 1♀; Pontianak; Muir F. leg.; USNMhttp://n2t.net/ark:/65665/3001083f7-393b-4949-9aca-a866dc50b311.

**Material examined (physically). *Non-type specimens***. Malaysia – Peninsular Malaysia • 1♂; “K. Kanyan, Pahang” [Pahang]; Gary R. leg.; ZRC ENT00066326. Singapore • 1♀; “Univ Campus” [National University of Singapore]; 03 Feb. 1974; Murphy D.H. leg.; Ficus sp.; ZRC ENT00066245 • 1♀; “Univ Campus bot. garden” [Singapore Botanic Gardens]; 22 Mar. 1975; Murphy D.H. leg.; ZRC ENT00066246 • 1♀, 2 Unknown sex; “Univ Campus” [National University of Singapore]; 22 Jun. 1976; Murphy D.H. leg.; Ficus benjamina; ZRC ENT00066247, ENT00066319 to ENT00066320 • 1♀; “Univ Campus” [National University of Singapore]; 30 Dec. 1976; Murphy D.H. leg.; ZRC ENT00066321 • 1♀; “Univ Campus” [National University of Singapore]; 29 Aug. 1978; Murphy D.H. leg.; Ficus retusa; ZRC ENT00066322 • 1♂; East Coast Park; 1.3008°N, 103.9122°E; 11 Oct. 2014; Ng S.Y. leg.; ZRC ENT00066323 • 1♀; Mandai Lake Road; 1.4093°N, 103.7801°E; 04 Sep. 2016; Rayner P.E. leg.; ZRC ENT00066324 • 1♀; Clementi Woods; 1.2978°N, 103.7681°E; 11 Sep. 2017; Liew H.X. leg.; ZRC ENT00066325.

##### *Tricentrus gibbosulus* (Walker, 1858)

*Centrotus gibbosulus* Walker, 1858a

**Type locality**. India (Walker 1858a).

**Recorded distribution**. Bangladesh, China, India, Indonesia, Malaysia, Pakistan, Singapore (Funkhouser 1918b).

**Host plant(s)**. *Amaranthus viridis* (Ahmad and Yasmeen 1978) (Amaranthaceae). *Mikania scandens*, *Xanthium strumarium* (Ahmad and Yasmeen 1978), *Xanthium* sp. (Ananthasubramanian 1996) (Asteraceae). *Santalum album* (Dover 1932) (Santalaceae). *Withania somnifera* (Ahmad and Yasmeen 1978) (Solanaceae). *Phyla nodiflora* (Ahmad and Yasmeen 1978) (Verbenaceae).

**Ant attendant(s)**. Unknown.

**Remarks**. Ahmad and Yasmeen (1978) reports this species to possess a white tomentosity on the metapleuron visible underneath the forewing, while females possess a darkening of the head and anterior portion of the metopidium relative to the rest of the pronotum. These traits are absent from the species description provided by Ananthasubramanian (1996). As Ahmad and Yasmeen (1978) and Ananthasubramanian (1996) both examined type material, these discrepancies warrant further investigation.

Ananthasubramanian (1996) noted males to possess shorter suprahumerals with posterior process not reaching 3ac.

##### *Tricentrus intermedius* (Schmidt, 1911)

*Otaris intermedius* Schmidt, 1911

**Type locality**. Sabah, Malaysia (Schmidt 1911).

**Recorded distribution**. Malaysia, Singapore (new record).

**Host plant(s)**. *Melastoma malabathricum* (Melastomataceae).

**Ant attendant(s)**. Unknown.

**Material examined (physically). *Non-type specimens***. Singapore • 1♀; “Bukit Timah Forest” [Bukit Timah Nature Reserve]; 27 Jun. 1976; Murphy D.H. leg.; ZRC ENT00066280 • 1♀; “Singapore Botanic Gardens nr. National Orchid Garden” [Singapore Botanic Gardens]; 09 Jul. 2021; Yap E.H. leg.; ZRC BDP0373609 • 1♂; “SBG, Gn Hop Extension” [Singapore Botanic Gardens, Gallop Extension]; 06 Jul. 2023; Yap E.H. leg.; on Melastoma malabathricum; ZRC BDP0372408.

##### *Tricentrus resectus* Distant, 1916

**Type locality**. Maymyo, Myanmar (Distant 1916a).

**Recorded distribution**. India, Indonesia, Malaysia, Myanmar, Singapore (Funkhouser 1918b).

**Host plant(s)**. Unknown.

**Ant attendant(s)**. Unknown.

**Remarks**. The sole description of *Tricentrus resectus* (Distant 1916a) is brief and insufficient to separate this species from its congeners. An examination of type(s) will be useful in clarifying its identity.

##### *Tricentrus spinicornis* Funkhouser, 1918

**Type locality**. Singapore (Funkhouser 1918b).

**Recorded distribution**. China, Indonesia, Malaysia, Singapore.

**Host plant(s)**. *Ficus heteromorpha* (Yuan and Chou 2002) (Moraceae).

**Ant attendant(s)**. Unknown

**Material examined (digitally). *Holotype***. Singapore • 1♀; 1917; Baker C.F. leg.; USNM 01113090 http://n2t.net/ark:/65665/37c0c9537-fe42-46e9-9bc9-9b66c894e352. ***Non-type specimens***. Malaysia – Peninsular Malaysia • 1 Unknown sex; “Cameron Highlands” [Pahang, Cameron Highlands]; 4800ft; 18 May 1931; Pendlebury H.M. leg.; at light; NHMUK 013387641 • 1 Unknown sex; “Mt. Dulit” [Sarawak, Mount Dulit]; 16 Oct. 1932; Hobby B.M. leg.; native collected, moss forest, 4000ft; NHMUK 013387638 • 1 Unknown sex; “Mt. Dulit” [Sarawak, Mount Dulit]; 2500ft; 19 Oct. 1932; Koyan R. leg.; light trap; NHMUK 013387639.

##### *Tricentrus spinis* Funkhouser, 1935

**Type locality**. Kuala Lumpur, Peninsular Malaysia (Funkhouser 1935b).

**Recorded distribution**. Malaysia, Singapore (Funkhouser (1935b).

**Host plant(s)**. *Hibiscus tiliaceus* (Malvaceae). *Melastoma malabathricum* (Melastomataceae).

**Ant attendant(s)**. Unknown.

**Remarks**. This species was first noted to occur in Singapore by Funkhouser (1935b), but this record was overlooked by subsequent catalogues and databases (Metcalf and Wade 1965; McKamey 1998; Dmitriev et al. 2022).

**Material examined (digitally). *Holotype***. Malaysia – Peninsular Malaysia • 1♀; Kuala Lumpur; Feb. 1933; Funkhouser W.D. leg.; USNM 01113094 http://n2t.net/ark:/65665/34c020d40-1d73-4cc9-9121-58f8ae006e87.

**Material examined (physically). *Non-type specimens***. Malaysia – Peninsular Malaysia • 1♀; “Maxwell Hill, Taiping, Perak T.VIII 64” [Perak, Maxwell Hill]; Murphy D.H. leg.; ZRC ENT00066541. Singapore • 1♀; “TOH TUCK EST. (BT. TIMAH)” [Toh Tuck]; 18 Apr. 1976; Murphy D.H. leg.; Wasteland with orchard and cattleshed; ZRC ENT00066536 • 3♀♀, 1♂; “Toh Tuck Wasteland” [Toh Tuck wasteland]; 10 Mar. 1978; Murphy D.H. leg.; On melastomom; ZRC ENT00066530 to ENT00066533 • 1♀, 1♂; Pulau Salu; 09 Sep. 1978; Murphy D.H. leg.; On Hib. tiliaceus; ZRC ENT00066534 to ENT00066535 • 3♂♂; Nee Soon Swamp Forest; 17 Jun. 1992; Yeo K.L. leg.; H.R. Light trap 1930–2130 hrs; ZRC ENT00066538 to ENT00066540 • 1♀; Kranji Nature Trail; 15 Mar. 2008; SBWR volunteers leg.; Grp 2 Sample 35; ZRC ENT00066537 • 1♂; “NUS Behind S2” [National University of Singapore]; 1.2965°N, 103.7779°E; 16 Sep. 2015; Lee W.Y. leg.; ZRC ENT00066542 • 1♂; Kent Ridge; 1.2960°N, 103.7761°E; 05 Oct. 2015; Phang Q.Y. leg.; ZRC ENT00066543 • 1♀; Kent Ridge Park; 1.2835°N, 103.7912°E; 20 Oct. 2015; Mhd Adom N.A. leg.; ZRC ENT00066544.

##### *Tricentrus truncaticornis* Funkhouser, 1918

*Otaris truncaticornis*: Goding 1934

**Type locality**. Singapore (Funkhouser 1918b).

**Recorded distribution**. Indonesia, Malaysia, Singapore.

**Host plant(s)**. Unknown.

**Ant attendant(s)**. Unknown.

**Material examined (digitally). *Holotype***. Singapore • 1♀; 1917; Baker C.F. leg.; USNM 01113137 http://n2t.net/ark:/65665/32c5ae023-4b60-4a11-a776-ca802bff8923. ***Non-type specimens***. Malaysia – East Malaysia • 1 Unknown sex; “foot of Mt. Dulit, Junction of rivers Tinjar and Lejok” [Sarawak, Foot of Mt. Dulit, Junction of rivers Tinjar and Lejok]; 11 May 1932; Hobby B.M., Moore A.W. leg.; clearing; NHMUK 013387636 • 1 Unknown sex; “foot of Mt. Dulit, Junction of rivers Tinjar and Lejok” [Sarawak, Foot of Mt. Dulit, Junction of rivers Tinjar and Lejok]; 12 Sep. 1932; Hobby B.M., Moore A.W. leg.; Light trap; NHMUK 013387637.

**Material examined (physically). *Non-type specimens***. Singapore • 1 Unknown sex; “Univ Campus” [National University of Singapore]; 08 Jun. 1979; Murphy D.H. leg.; Eugenia tomentosa; ZRC ENT00066281.

##### *Tricentrus cf. albomaculatus* Distant, 1906

**Host plant(s)**. *Mikania* sp. (Asteraceae). *Acacia* sp., *Parkia* sp. (Fabaceae).

**Ant attendant(s)**. Unknown.

**Remarks**. Male unknown. This morphospecies differs from *Tricentrus albomaculatus* by the presence of white tomentosity at the base of the scutellum and the tip of the posterior process which is not distinctly upturned.

**Material examined (physically). *Non-type specimens***. Malaysia – Peninsular Malaysia • 1♀; “JOHORE, 8km off KULAI, TOWARDS KOTA TINGGI” [Johor, near Kulai]; 29 Dec. 1988; Koo Y.H. leg.; ZRC ENT00066565 • 1♀, 1 Unknown sex; “Sedili Besar Johor, West Malaysia” [Johor, Sedili Besar]; 25 Nov. 1991; Keong Y.S. leg.; ZRC ENT00066564, ENT00066566. Singapore • 1♀; “Jurong. Spore.” [Jurong]; Sep. 1965; Murphy D.H. leg.; ZRC ENT00066556 • 1♀; “Yeo Chukang Rd.” [Yio Chu Kang]; 1967; Murphy D.H. leg.; ZRC ENT00066558 • 1♀; Kent Ridge; 23 Mar. 1975; Murphy D.H. leg.; ZRC ENT00066554 • 1♀; Pasir Ris; 01 May 1975; Murphy D.H. leg.; ZRC ENT00066553 • 1♀; “Clementi Dump “ [Clementi]; 17 Sep. 1977; Murphy D.H. leg.; Parkia (leg); ZRC ENT00066560 • 1♀; Clementi; 16 Oct. 1977; Murphy D.H. leg.; Acacia shoots, scrub; ZRC ENT00066557 • 2♀♀; “Bukit Timah campus” [Bukit Timah]; 28 Feb. 1980; Murphy D.H. leg.; Mikania; ZRC ENT00066561 to ENT00066562 • 1♀; Ulu Pandan; 02 Mar. 1983; Murphy D.H. leg.; ZRC ENT00066559 • 1♀; Commonwealth; 18 Nov. 1991; Lee P.Y. leg.; ZRC ENT00066563 • 1♀; “Nee Soon Forest” [Nee Soon Swamp Forest]; 27 Mar. 2005; Wang L.K. leg.; ZRC ENT00066570 • 1♀; “1.3599, 103.7458” [Bukit Batok]; 1.3599°N, 103.7458°E; 22 Oct. 2013; R. L. leg.; ZRC ENT00066567 • 1♀; “Dairy Farm Estate” [Dairy Farm Road]; 23 Aug. 2014; Ong C.N.Y. leg.; ZRC ENT00066568 • 1♀; “Green corridor” [Rail Corridor]; 1.3135°N, 103.7890°E; 17 Oct. 2014; Lim M.S. leg.; ZRC ENT00066569 • 1♀; “Choa Chu Kang, 1.3832, 103.7573” [Choa Chu Kang]; 1.3832°N, 103.7573°E; 10 Nov. 2014; Yue R.B. leg.; ZRC ENT00066575 • 1♀; “NUS University Hall” [National University of Singapore]; 1.2973°N, 103.7768°E; 10 Sep. 2015; Eschenbrenner J. leg.; ZRC ENT00066573 • 1♀; Kent Ridge; 1.2952°N, 103.7770°E; 11 Sep. 2015; Poh T.S.M. leg.; ZRC ENT00066574 • 1♀; Kent Ridge; 1.2953°N, 103.7757°E; 16 Sep. 2015; Khoo M.D.Y. leg.; ZRC ENT00066571 • 1♀; 1 Lim Chu Kang Lane 3A; 1.4310°N, 103.7078°E; 20 Sep. 2015; Tan S.T.M. leg.; ZRC ENT00066579 • 1♀; Bukit Timah Railway Station; 1.3340°N, 103.7812°E; 22 Sep. 2015; Lum B. leg.; ZRC ENT00066572 • 1♀; Palawan Beach Sentosa; 1.2477°N, 103.6241°E; 15 Oct. 2016; Tok E. leg.; ZRC ENT00066578 • 2♀♀; Holland Woods; 1.3338°N, 103.7813°E; 18 Oct. 2016; Ang Y.T. leg.; ZRC ENT00066576 to ENT00066577 • 1♀; Lorong Chencharu; 1.4154°N, 103.8313°E; 08 Oct. 2017; Ng S.T. leg.; ZRC ENT00066580.

#### Leptobelini Yuan, 2002

***Leptobelus* Stål, 1866**

##### *Leptobelus aff. elevatus* Funkhouser, 1929

**Host plant(s)**. Unknown.

**Ant attendant(s)**. Unknown.

**Remarks**. Male unknown. This morphospecies differs from *Leptobelus elevatus* by the white tomentosity on the scutellum being formed as two distinct patches rather than a single, continuous band.

**Material examined (physically). *Non-type specimens***. Singapore • 1♀; “1.3542, 103.8264” [Windsor Nature Park]; 1.3542°N, 103.8264°E; 06 Nov. 2013; L.R. leg.; ZRC ENT00066604 • 1♀; Murphy D.H. leg.; ZRC ENT00062958.

#### Leptocentrini Distant, 1908

***Leptocentrus* Stål, 1866**

##### *Leptocentrus leucaspis* (Walker, 1858)

*Centrotus taurus*: Walker 1851 (in part)

*Centrotus leucaspis* Walker, 1858b

**Type locality**. Unspecified (Walker 1851; Walker 1858b).

**Recorded distribution**. Bangladesh, Brunei, China, India, Indonesia, Malaysia, Myanmar, Pakistan, Philippines, Singapore (Funkhouser 1918b), Sri Lanka.

**Host plant(s)**. *Terminalia catappa* (Ananthasubramanian 1996) (Combretaceae). *Bauhinia tomentosa*, *Pongamia pinnata* (Ananthasubramanian 1996) (Fabaceae).

**Ant attendant(s)**. *Solenopsis geminata* (Ananthasubramanian 1996).

**Remarks**. It is challenging to distinguish *Leptocentrus leucaspis* from its congeners based on available descriptions (Walker 1858b; Buckton 1903; Distant 1908; Funkhouser 1915; Ananthasubramanian 1996). Curiously, Buckton (1903) reported the species to possess two white tomentose spots between the suprahumerals and the posterior process, a trait not observed by other workers.

Funkhouser (1915) noted the males of this species to be smaller and darker than females. Ananthasubramanian (1996), while agreeing that the males were smaller, noted them to be of a lighter colouration instead. This may suggest sexual multichromatism in the species, but further investigation is required to confirm this.

##### *Leptocentrus longispinus* Distant, 1908

**Type locality**. Myanmar (Distant 1908).

**Recorded distribution**. China, India, Indonesia, Malaysia, Myanmar, Singapore (Funkhouser 1918b).

**Host plant(s)**. *Magnolia champaca* (Ananthasubramanian 1996) (Magnoliaceae).

**Ant attendant(s)**. Unknown.

**Remarks**. Distant (1908) remarked *Leptocentrus longispinus* as a species “to be recognised” by the length of its suprahumerals. However, Ananthasubramanian (1996), based on examination of the holotype, reported that *L. longispinus* shared similar suprahumeral proportions to *L. leucaspis*, weakening the value of suprahumeral length as a diagnostic character. Notably, Ananthasubramanian’s treatment of *L. leucaspis* was not based on type(s). Ananthasubramanian (1996) further distinguished *L. longispinus* and *L. leucaspis* by features of the frontoclypeus, ocelli, and tegmina, but these differences were not clearly articulated. Subsequently, Yuan and Chou (2002) provided a description of *L. longispinus* differing in several respects from previous accounts. Most notably, they described its suprahumerals as being produced nearly horizontally in frontal view rather than obliquely as described by Distant (1908) and Ananthasubramanian (1996). It is unlikely that Yuan and Chou (2002) examined type material. The identity of this species warrants re-examination through comparison of Type specimens.

##### *Leptocentrus obortus* Distant, 1916

**Type locality**. Mawlamyine, Myanmar (Distant 1916a).

**Recorded distribution**. India, Indonesia, Malaysia, Myanmar, Singapore (Funkhouser 1918b).

**Host plant(s)**. Unknown.

**Ant attendant(s)**. Unknown.

**Remarks**. Based on examination of non-types (2 females and 1 male), Ananthasubramanian (1996) noted that males of this species bore a white tomentose patch on the frontoclypeus, a trait absent in the females. In the original description of *L. obortus*, Distant (1916a) similarly described a large, rounded white tomentose spot on the “face of head”. Although Distant did not state the sex of the type examined, it is likely to have been a male (Broomfield 1971). Regrettably, Broomfield (1971) notes the head of Distant’s type to be missing. A white tomentose patch on the frontoclypeus, exclusive to males, is an unusual feature and may prove useful as a diagnostic character. Examination of physical specimens would help clarify the constancy, size, and shape of this trait.

##### *Leptocentrus taurus* (Fabricius, 1775)

*Membracis taurus* Fabricius, 1775

*Membracis rupicapra* Fabricius, 1798

*Centrotus rupicapra*: Fabricius 1803

*Centrotus scutellaris* Fabricius, 1803

*Centrotus taurus*: Fabricius 1803

*Membracis tricornis* Hardwicke, 1829

*Centrotus tricornis*: Dohrn 1859

*Leptobelus scutellaris*: Atkinson 1885

*Leptocentrus gazella* Buckton, 1903

*Aetalion scutellaris*: Sherborn 1930

**Type locality**. “India orientali” [Southeast Asia] (Fabricius 1775).

**Recorded distribution**. Afghanistan, Bangladesh, Cambodia, China, East Timor, Guam, India, Indonesia, Japan, Malaysia, Mariana Islands, Myanmar, Pakistan, Philippines, Singapore (Distant 1916a), Sri Lanka, Suriname, Thailand, Vietnam.

**Host plant(s)**. *Eranthemum* sp. (Ananthasubramanian 1996) (Acanthaceae). *Anacardium occidentale*, *Mangifera indica* (Ananthasubramanian 1996) (Anacardiaceae). *Artabotrys* sp., *Monoon longifolium* (Ananthasubramanian 1996), *Cananga odorata* (Yuan and Chou 2002) (Annonaceae). *Kalopanax septemlobus* (Yuan and Chou 2002) (Araliaceae). *Capparis sepiaria*, *Crateva religiosa* (Ananthasubramanian 1996) (Capparaceae). *Casuarina equisetifolia* (Ananthasubramanian 1996) (Casuarinaceae). *Ipomoea pes-caprae* (Ananthasubramanian 1996) (Convolvulaceae). *Acaciella glauca*, *Intsia bijuga* (Swezey 1942), *Abrus precatorius*, *Albizia lebbeck*, *Arachis hypogaea*, *Butea monosperma*, *Caesalpinia pulcherrima*, *Canavalia ensiformis*, *Cassia fistula*, *Clitoria ternatea*, *Crotalaria juncea*, *Crotalaria verrucosa*, *Cyamopsis tetragonoloba*, *Delonix regia*, *Lablab purpureus*, *Lathyrus aphaca*, *Lathyrus odoratus*, *Libidibia coriaria*, Macrotyloma biflorum var. biflorum, *Mimosa rubicaulis*, *Neltuma juliflora*, *Parkia biglandulosa*, *Parkinsonia aculeata*, *Prosopis cineraria*, *Senna auriculata*, *Sesbania sesban*, *Vachellia nilotica*, *Vigna mungo* (Ananthasubramanian 1996), *Erythrina variegata* (Swezey 1942; Ananthasubramanian 1996), *Tamarindus indica* (Ananthasubramanian 1996; Yuan and Chou 2002) (Fabaceae). *Hernandia* sp. (Swezey 1942) (Hernandiaceae). *Cinnamomum verum* (Ananthasubramanian 1996) (Lauraceae). *Hibiscus tiliaceus*, *Malachra capitata*, *Thespesia populnea* (Swezey 1942), *Gossypium arboreum*, *Gossypium hirsutum* (Khan 1949), *Gossypium herbaceum*, *Hibiscus rosa-sinensis*, *Sida cordifolia* (Ananthasubramanian 1996), *Hibiscus syriacus* (Yuan and Chou 2002) (Malvaceae). *Melastoma malabathricum* (Melastomataceae). *Psidium guajava* (Ananthasubramanian 1996) (Myrtaceae). *Adinandra dumosa* (Pentaphylacaceae). *Glochidion marianum* (Swezey 1942) (Phyllanthaceae). *Ziziphus oenopolia* (Ananthasubramanian 1996), *Ziziphus jujuba* (Dover 1932; Ananthasubramanian 1996; Yuan and Chou 2002) (Rhamnaceae). *Coffea arabica* (Yuan and Chou 2002), *Morinda* sp. (Ananthasubramanian 1996) (Rubiaceae). *Bergera koenigii*, *Limonia acidissima* (Ananthasubramanian 1996) (Rutaceae). *Santalum album* (Dover 1932) (Santalaceae). *Datura metel*, *Solanum lycopersicum*, *Solanum nigrum*, *Solanum torvum*, *Solanum trilobatum* (Ananthasubramanian 1996), *Solanum melongena* (Ananthasubramanian 1996; Yuan and Chou 2002) (Solanaceae). *Pipturus argenteus* (Swezey 1942) (Urticaceae). *Lantana* sp. (Dover 1932) (Verbenaceae). *Cissus quadrangularis* (Ananthasubramanian 1996) (Vitaceae).

**Ant attendant(s)**. *Camponotus compressus*, *Oecophylla smaragdina* (Ananthasubramanian 1996).

**Remarks**. Earlier descriptions of *Leptocentrus taurus* (Fabricius 1775, 1798, 1803; Hardwicke 1829; Fairmaire 1846b; Atkinson 1885; Buckton 1903) are vague and hinder unequivocal interpretation of the species. Distant (1908) provided a first somewhat clear delineation, although it is unlikely that he examined Fabricius’ original specimen(s) (Distant 1908). Nonetheless, Funkhouser (1915) regarded Distant’s figure as “typical” of *L. taurus*, and subsequent descriptions of the species (Ananthasubramanian 1996; Yuan and Chou 2002) are generally in agreement with Distant (1908).

Ananthasubramanian (1996) considered *L. terminalis* (Walker, 1851) and *L. vicarius* (Walker 1851) as synonyms of *L. taurus*. Contrastingly, Goding (1939) and Yuan and Chou (2002) considered *L. terminalis* as synonymous with *L. taurus*, but not *L. vicarius*. Notably, the major catalogues of the Membracidae (Funkhouser 1927b; Metcalf and Wade 1965; McKamey 1998) unanimously treat *L. taurus*, *L. terminalis*, and *L. vicarius* as separate species. Funkhouser (1915) noted variation in size, colouration, and position of suprahumerals upon examination of a large series *L. taurus*, and this may have been a source of confusion amongst authors. Indeed, descriptions of all three species are scarcely differentiable, and it is likely that *L. taurus* is the senior synonym for all of them. Nonetheless, this can only be confirmed via a future revision referring to types.

This species is exceptionally polyphagous with a total of 72 recorded host plant species spread across 23 plant families.

**Material examined (physically). *Non-type specimens***. Malaysia • 1♀; “Kota Tinggi” [Johor, Kota Tinggi]; 28 Oct. 1988; ZRC ENT00066437 • 1♀; Pahang, Roman Sungei Kinchin Base Camp; 15 Jun. 1989; Koo Y.H. leg.; ZRC ENT00066436 • 1♀; Johor, Sedili Besar; 25 Nov. 1991; Yong W.H. leg.; ZRC ENT00066435. Singapore • 3♀♀; “M. 7aber” [Mount Faber]; Oct. 1962; Murphy D.H. leg.; On melastomom, Melastomom; ZRC ENT00066422 to ENT00066424 • 1♀; “Nee Soon” [Yishun]; 07 Jan. 1968; Murphy D.H. leg.; ZRC ENT00066425 • 1♂; Jalan Kayu; 19 Mar. 1975; Murphy D.H. leg.; ZRC ENT00066428 • 1♀; “Mt. Faber” [Mount Faber]; 21 Mar. 1978; Murphy D.H. leg.; On melastomom; ZRC ENT00066426 • 5♀♀, 2 Nymph; “Rifle range Rd” [Rifle Range Road]; 17 Apr. 1978; Murphy D.H. leg.; On melastomom; ZRC ENT00066312 to ENT00066318 • 1♂; “Tyersall Ave” [Singapore Botanic Gardens]; 09 Aug. 1978; Murphy D.H. leg.; On melastomom; ZRC ENT00066427 • 1♀; “Rifle range Rd” [Rifle Range Road]; 01 Oct. 1986; J.L.Y.Y. leg.; shrub; ZRC ENT00066431 • 1♀; “P. UBIN “ [Pulau Ubin]; 07 Nov. 1988; Gan V.N. leg.; “BELUKAR”; ZRC ENT00066429 • 1♀; “Kent Ridge secondary forest group” [Secondary forest at Kent Ridge]; 08 Nov. 1988; ZRC ENT00066430 • 1♀; “Lim Chu Kang Mangrove Fringe” [Fringe of mangrove at Lim Chu Kang]; 08 Apr. 1989; Lee S.C. leg.; ZRC ENT00066432 • 1♀; “Tampines bike trail “ [Tampines Bike Trail]; 14 Oct. 2012; Unadirekkul P. leg.; ZRC ENT00066433 • 1♂; St. John’s Island; 1.2218°N, 103.8479°E; 08 Sep. 2014; Ascher J.S. leg.; ZRC ENT00066434.

##### *Leptocentrus* sp.

**Host plant(s)**. *Melastoma malabathricum* (Melastomataceae).

**Ant attendant(s)**. Unknown.

**Material examined (physically)**. Malaysia – Peninsular Malaysia • 1♂; Penang Hill; 25 Oct. 1960; 1500; ZRC ENT00066292 • 1♀; “Johor, Forest nr Sungei Emas” [Johor, Forest near Sungei Emas]; 11 Nov. 1974; Murphy D.H. leg.; ZRC ENT00066291 • 1♂; “Selangor, Batu Cave” [Selangor, Batu Caves]; Feb. 1996; Chen L. leg.; ZRC ENT00066293. Singapore • 1♀; Pulau Ubin, Ubin Living Lab; 1.4070°N, 103.9545°E; 09 Sep. 2017; Lim N. leg.; ZRC ENT00066290 • 1♀; Sentosa, Fort Imbiah; 1.2570°N, 103.8150°E; 21 Feb. 2022; Feng L. leg.; caught sweep netting grasses and shrubs; ZRC ENT00066294.

#### *Nilautama* Distant, 1908

##### *Nilautama minutispina* Funkhouser, 1918

**Type locality**. Penang, Peninsular Malaysia (Funkhouser 1918b).

**Recorded distribution**. Indonesia, Malaysia, Singapore (new record).

**Host plant(s)**. *Annona muricata* (Annonaceae). *Calophyllum inophyllum* (Calophyllaceae). *Terminalia catappa*, *Terminalia* sp. (Combretaceae). *Dillenia suffruticosa* (Dilleniaceae). *Vitex* sp. (Lamiaceae). *Melastoma malabathricum* (Melastomataceae). *Ficus* sp., *Streblus elongatus* (Moraceae). *Syzygium grande* (Myrtaceae).

**Ant attendant(s)**. *Oecophylla smaragdina*.

**Material examined (digitally). *Holotype***. Malaysia – Peninsular Malaysia • 1♀; 1917; Baker C.F. leg.; From leaf of a small shrub; USNMhttp://n2t.net/ark:/65665/31bea3927-3eb5-4a38-9d30-c8f92d3cdee1.

**Material examined (physically). *Non-type specimens***. Malaysia • 2♀♀; 26 Nov. 1991; Kok T.Q. leg.; ZRC ENT00066027 to ENT00066028. Singapore • 2♀♀; “Lim Chu Kang Citrus Pltn.” [Lim Chu Kang citrus plantation]; 08 Jun. 1976; Murphy D.H. leg.; On soursop fruit, Oecophylla sp.; ZRC ENT00066002 to ENT00066003 • 1♀; Fort Canning; 09 Mar. 1978; Murphy D.H. leg.; On vitex; ZRC ENT00066004 • 1♀, 1 Nymph; “Botanic Gdns” [Singapore Botanic Gardens]; 14 Mar. 1978; Murphy D.H. leg.; ZRC ENT00066020 to ENT00066021 • 5♀♀, 1♂, 9 Nymph; Fort Canning; 16 Mar. 1978; Murphy D.H. leg.; On Ficus retusa; ZRC ENT00066005 to ENT00066019 • 4♀♀; “Tanglin Gardens” [Singapore Botanic Gardens]; 25 Apr. 1979; Murphy D.H. leg.; On termimalia; ZRC ENT00065998 to ENT00066001 • 1 Unknown sex; “KENT RIDGE” [Kent Ridge]; 14 Jan. 1982; Koo Y.H. leg.; ZRC ENT00066023 • 1♀; Kent Ridge; 01 Dec. 1991; Tan G.S. leg.; ZRC ENT00066024 • 1♂; “Science Dr.4 “ [National University of Singapore, Science Drive 4]; 07 May 2012; Teo L.Y. leg.; ZRC ENT00066699 • 1♀; St. John’s Island; 1.2218°N, 103.8479°E; 08 Sep. 2014; Ascher J.S. leg.; ZRC ENT00066025 • 1♀; Holland Woods; 1.3275°N, 103.7815°E; 04 Nov. 2015; Tjong F.A. leg.; Net collected; ZRC ENT00066026 • 2♀♀; St. John’s Island; 1.2195°N, 103.8484°E; 18 Sep. 2016; Chang Y.W.K., Chui S.X., Tan Y.G., Yip Y.X.C. leg.; ZRC ENT00066700, ENT00066702 • 1 Unknown sex; Murphy D.H. leg.; ZRC ENT00066022 • 1♀; Bukit Batok Hillside Nature Park; 1.3540°N, 103.7400°E; Zeng J. leg.; ZRC ENT00065997.

#### Maarbarini Wallace & Deitz, 2004

***Telingana* Distant, 1908**

##### *Telingana recurvata* Distant, 1916

**Type locality**. Sarawak, Malaysia (Distant 1916c).

**Recorded distribution**. Indonesia, Malaysia, Singapore (new record).

**Host plant(s)**. *Antidesma bunius* (Phyllanthaceae). *Uncaria* sp. (Rubiaceae). *Poikilospermum suaveolens* (Urticaceae).

**Ant attendant(s)**. Unknown.

**Material examined (physically). *Non-type specimens***. Singapore • 1♂; “Bukit Timah Forest” [Bukit Timah Nature Reserve]; 23 Jan. 1974; Murphy D.H. leg.; On Uncaria; ZRC ENT00066703 • 1♂; “Bukit Timah Forest” [Bukit Timah Nature Reserve]; 27 Jan. 1974; Murphy D.H. leg.; Tree trunk; ZRC ENT00066704 • 1♀; “Hindhede Dr.” [Hindhede Drive]; 19 Nov. 1989; Lua H.K. leg.; LHK82; ZRC ENT00066705 • 1♀; Near MacRitchie Reservoir; 25 May 2024; Tan V. leg.; ZRC ENT00062986.

#### Micreunini Distant, 1908

***Micreune* Walker, 1857**

##### *Micreune formidanda* Walker, 1857

*Micreune quadrilinea* Walker, nom. nud. (Distant 1915)

**Type locality**. Singapore (Walker 1857).

**Recorded distribution**. India, Malaysia, Singapore.

**Host plant(s)**. Unknown.

**Ant attendant(s)**. Unknown.

**Material examined (digitally). *Lectotype***. Singapore • 1♀; Wallace A.R. leg.; “81116”; NHMUK 015028869.

#### Terentiini Haupt, 1929

***Ceraon* Buckton, 1903**

##### *Ceraon orientale* (Funkhouser, 1918)

*Xiphistes orientalis* Funkhouser, 1918

*Sinenodus orientalis*: Goding 1950

**Type locality**. Singapore (Funkhouser 1918b).

**Recorded distribution**. Malaysia, Singapore.

**Host plant(s)**. Unknown.

**Ant attendant(s)**. Unknown.

**Remarks**. Examination of ZRC specimens suggest that this species may not belong to tribe Terentiini as it posseses ab- and adlateral cucullate setae on the mesothoracic femur. Further investigations are required to ascertain a more accurate tribal and genus placement for this species.

**Material examined (physically). *Non-type specimens***. Singapore • 1♀; “Bukit Timah village wasteland” [Bukit Timah]; 21 Mar. 1976; Murphy D.H. leg.; ZRC ENT00062961 • 1♂; Cluny Road; 21 Jun. 1976; Murphy D.H. leg.; ZRC ENT00062962 • 1♀; Singapore Botanic Gardens, The Rainforest; 11 Jan. 2021; Yap E.H. leg.; ZRC ENT00062960.

#### *Eufrenchia* Goding, 1903

##### *Eufrenchia laminifer* (Buckton, 1903)

*Ibiceps laminifer* Buckton, 1903

*Centrotypus lamifer*: Goding 1926 (sic)

**Type locality**. Unspecified (Buckton 1903: 240).

**Recorded distribution**. Australia, Singapore.

**Singapore record**: Buckton (1903).

**Host plant(s)**. Unknown.

**Ant attendant(s)**. Unknown.

**Remarks**. Buckton (1903) described this species based on a specimen marked with habitat “Singapore”, but also with an accompanying label titled “Australia”. All known congeners to *Eufrenchia laminifer* are restricted to the Australasian region (Dmitriev et al. 2022), hence it is strange that this lone species occurs in Singapore. In this regard, the type locality of this species remains ambiguous and requires further clarification.

### Key to species of treehoppers from Singapore

**Table:** 

1	Distal end of mesothoracic femur without ab- and adlateral cucullate setae (a)	**2**
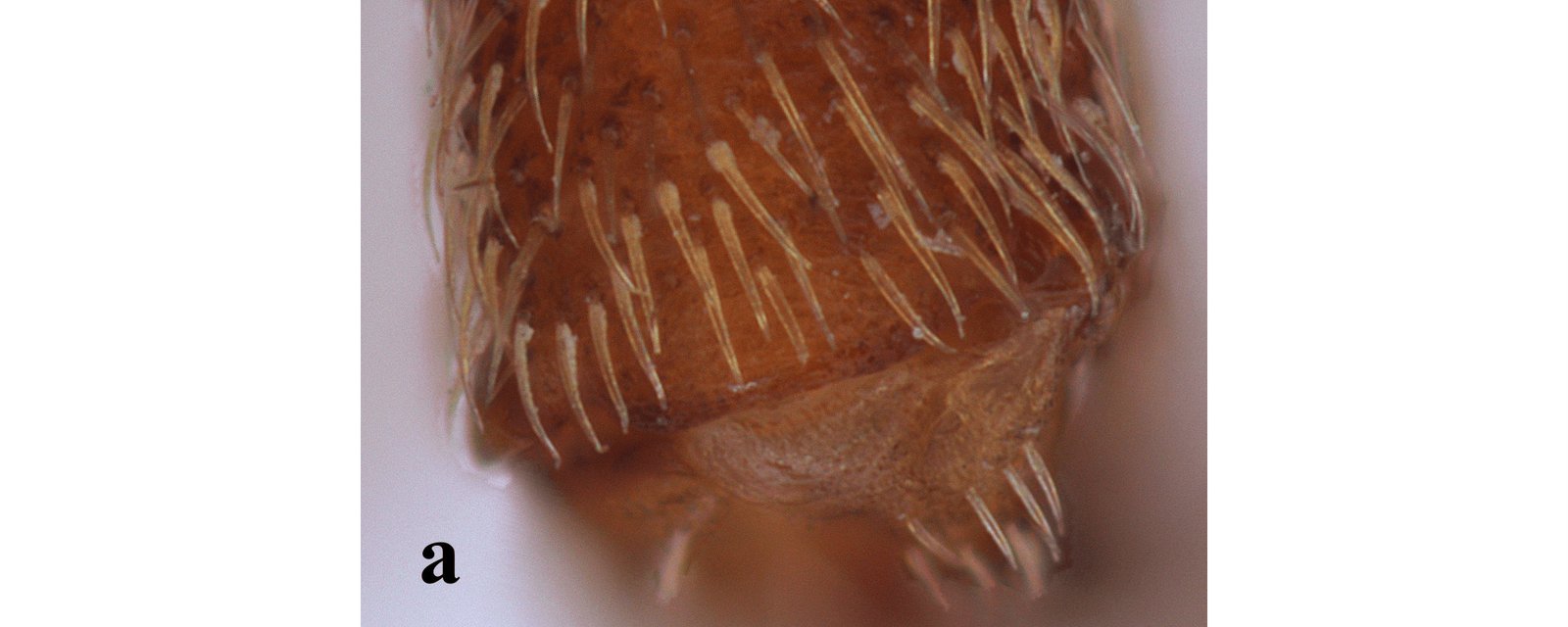		
–	Distal end of mesothoracic femur with ab- and adlateral cucullate setae (b)	**3**
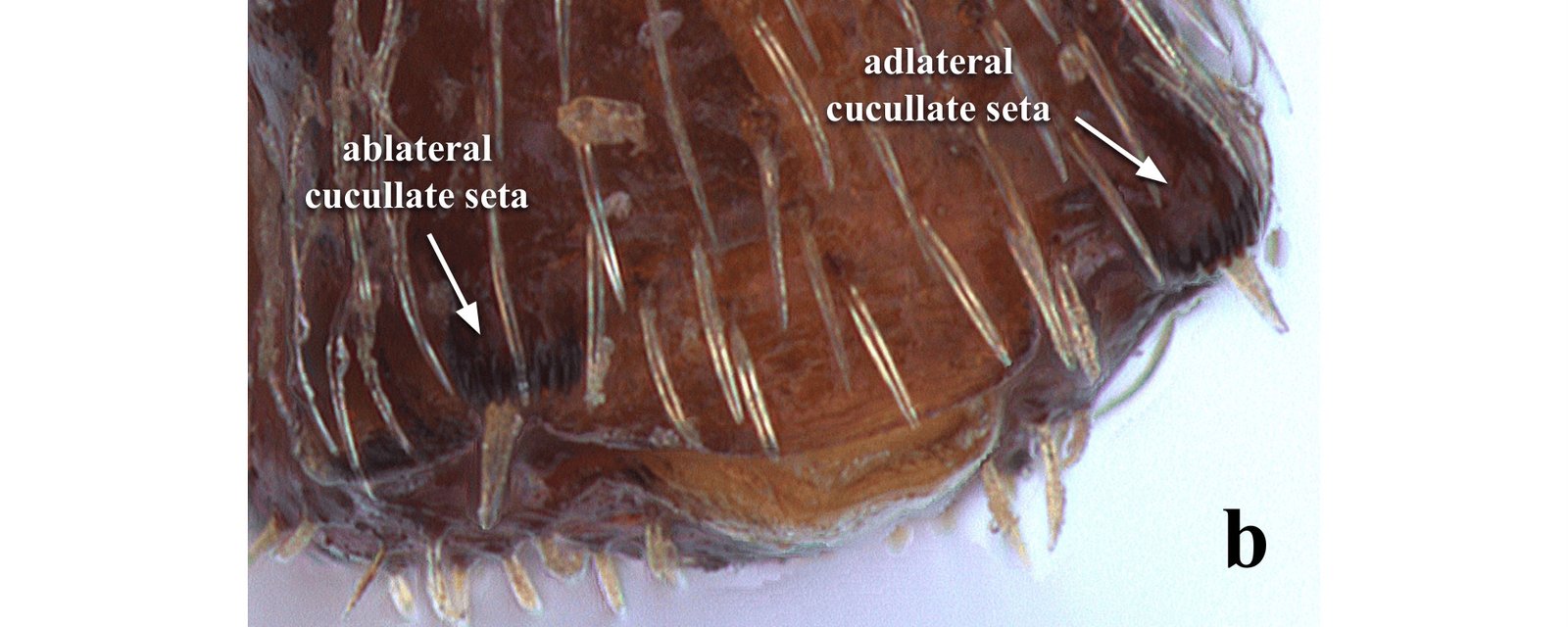		
2	Posterior process generally straight (a)	***Eufrenchia laminifer* (Buckton, 1903)**
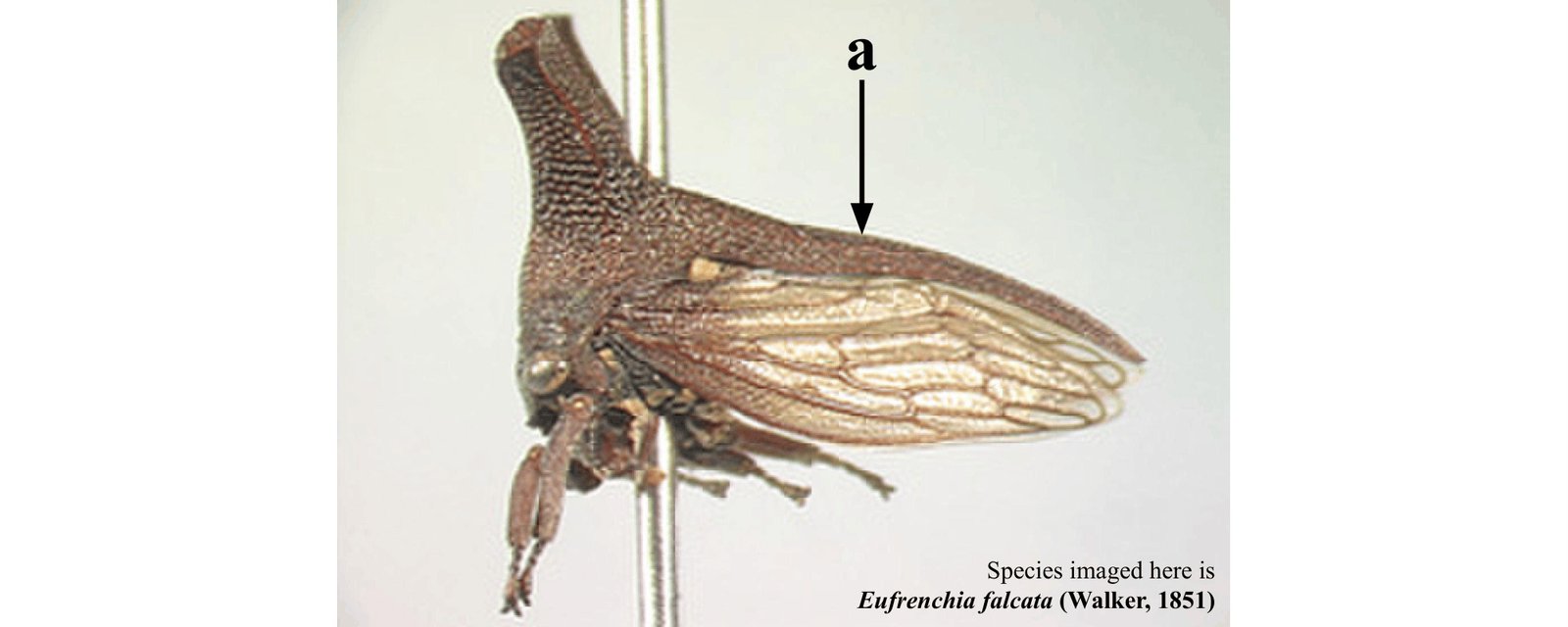		
–	Posterior process distinctly sinuate (b)	**4**
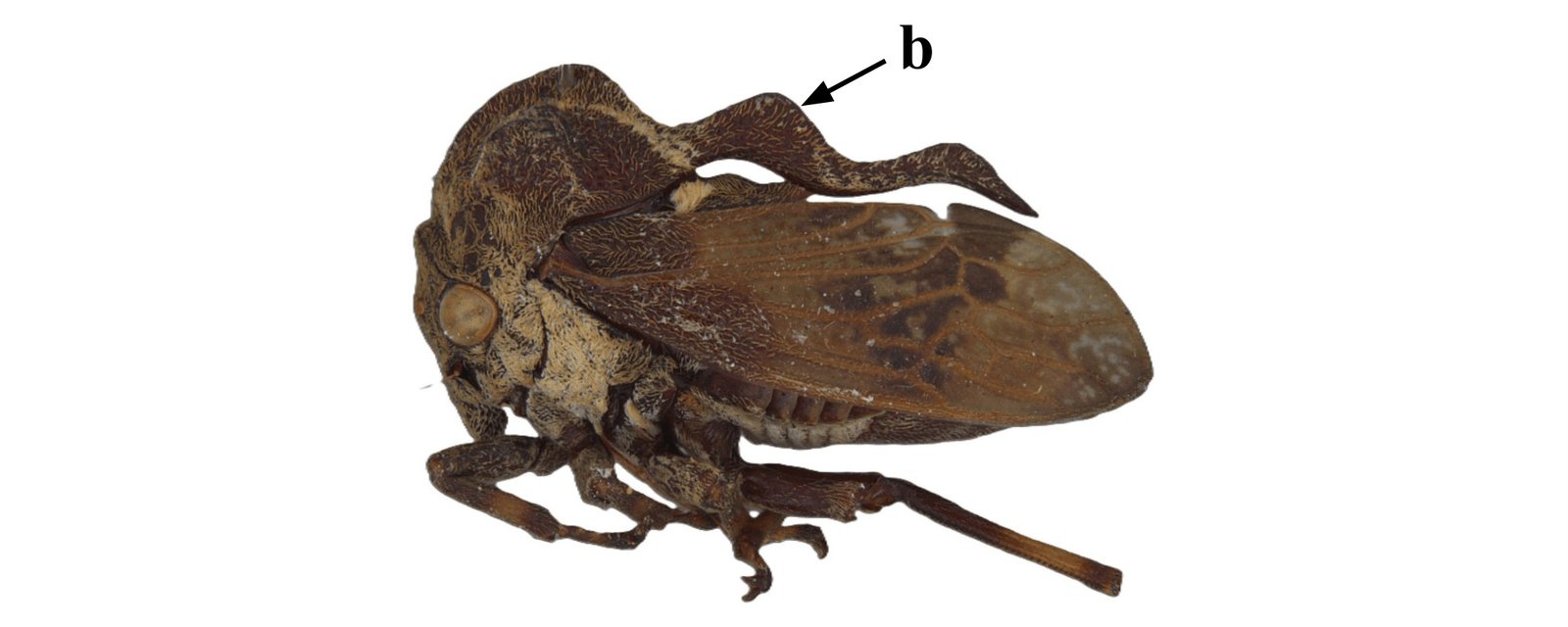		
3	Posterior process well raised over the scutellum (a); scutellum entirely exposed from lateral view (b)	**5**
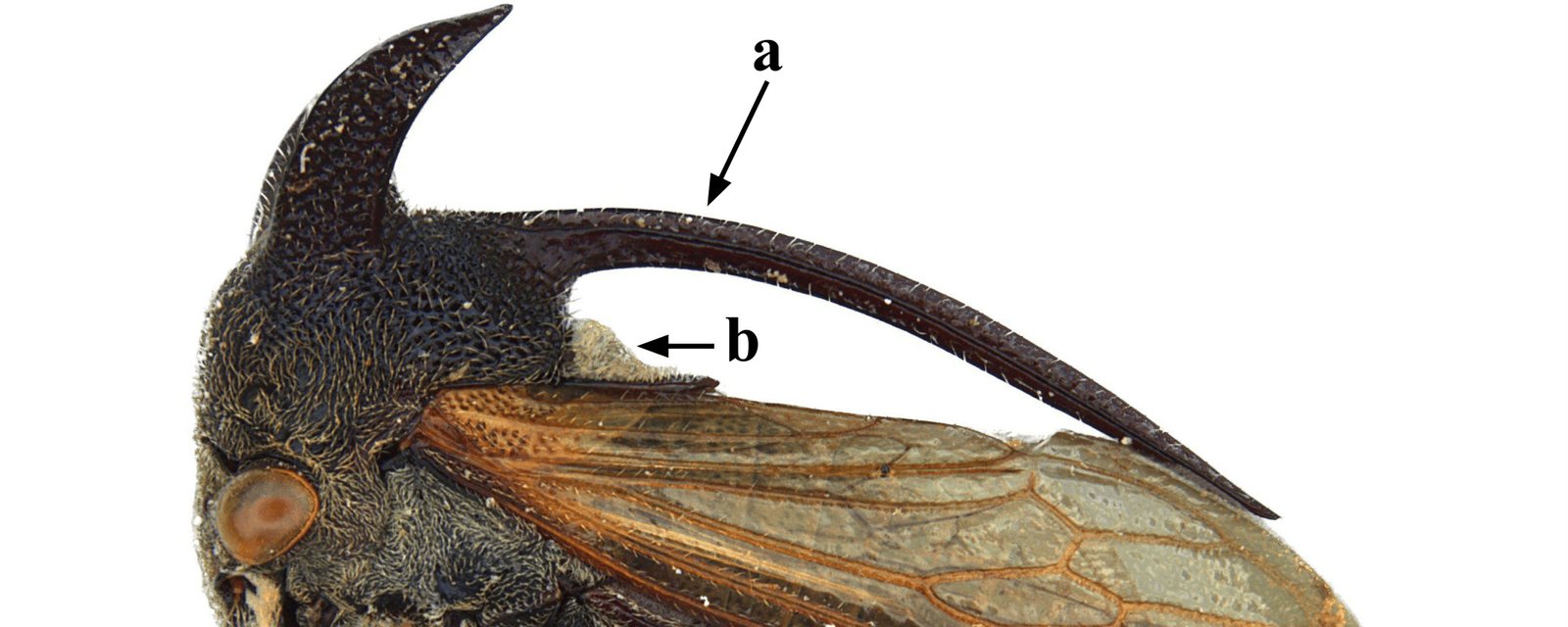		
–	Posterior process only slightly raised over scutellum (c), or posterior process appressed against scutellum with scutellum partially visible from lateral view (d), or posterior process entirely obscuring scutellum from lateral view (e)	**6**
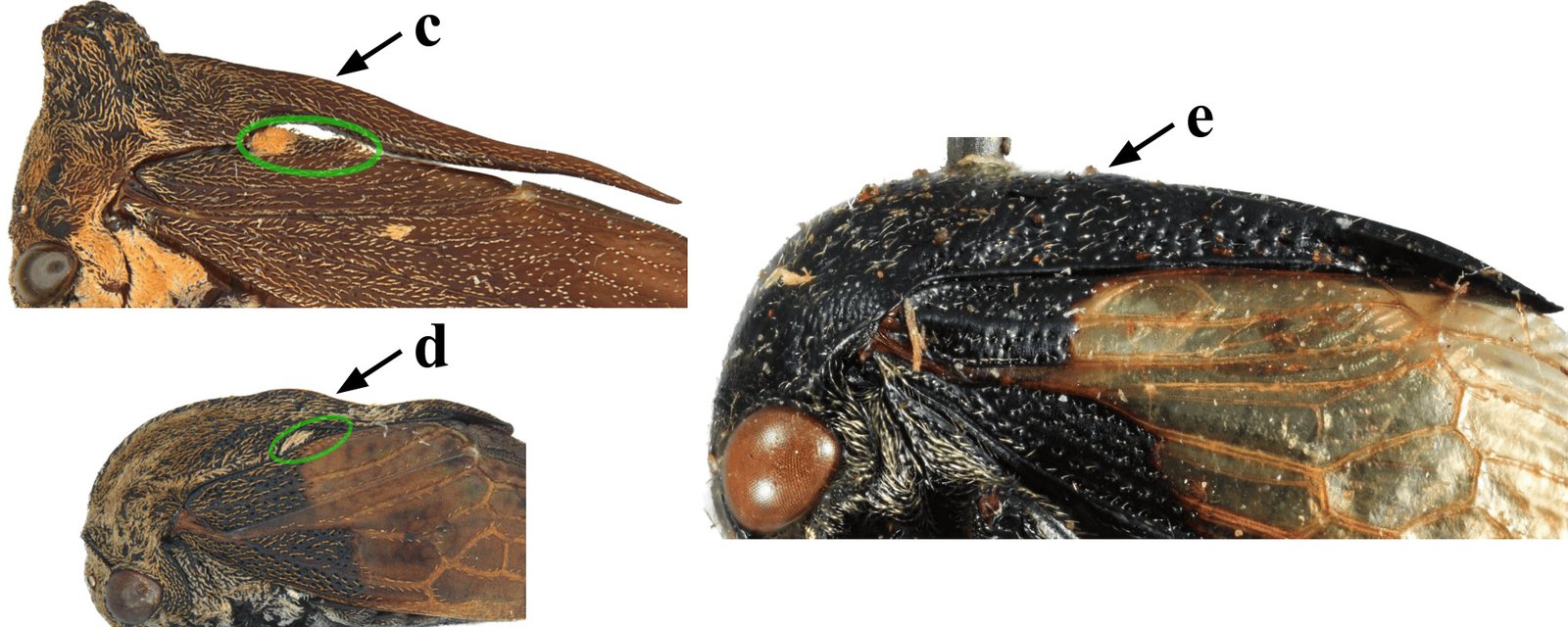		
4	Tibial surface nodulate (a)	***Centrochares ridleyana* Distant, 1915**
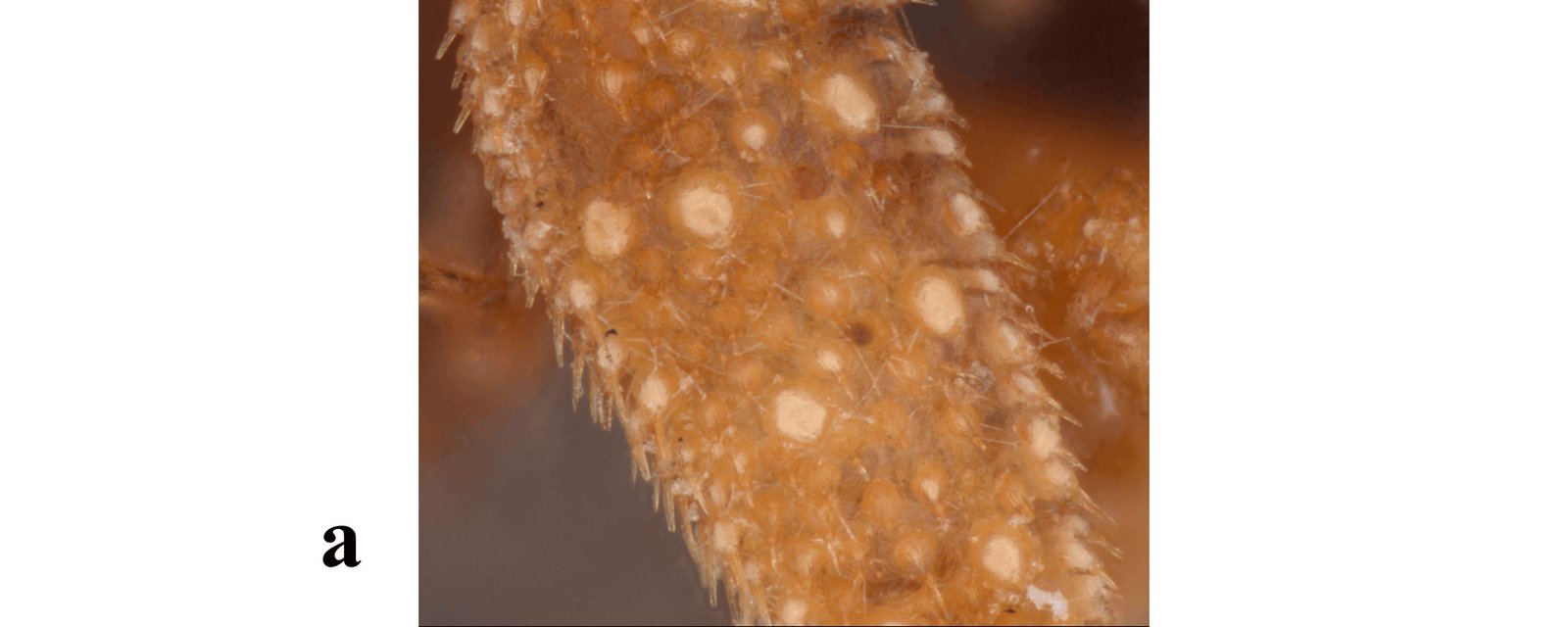		
–	Tibial surface smooth without nodules (b)	**7**
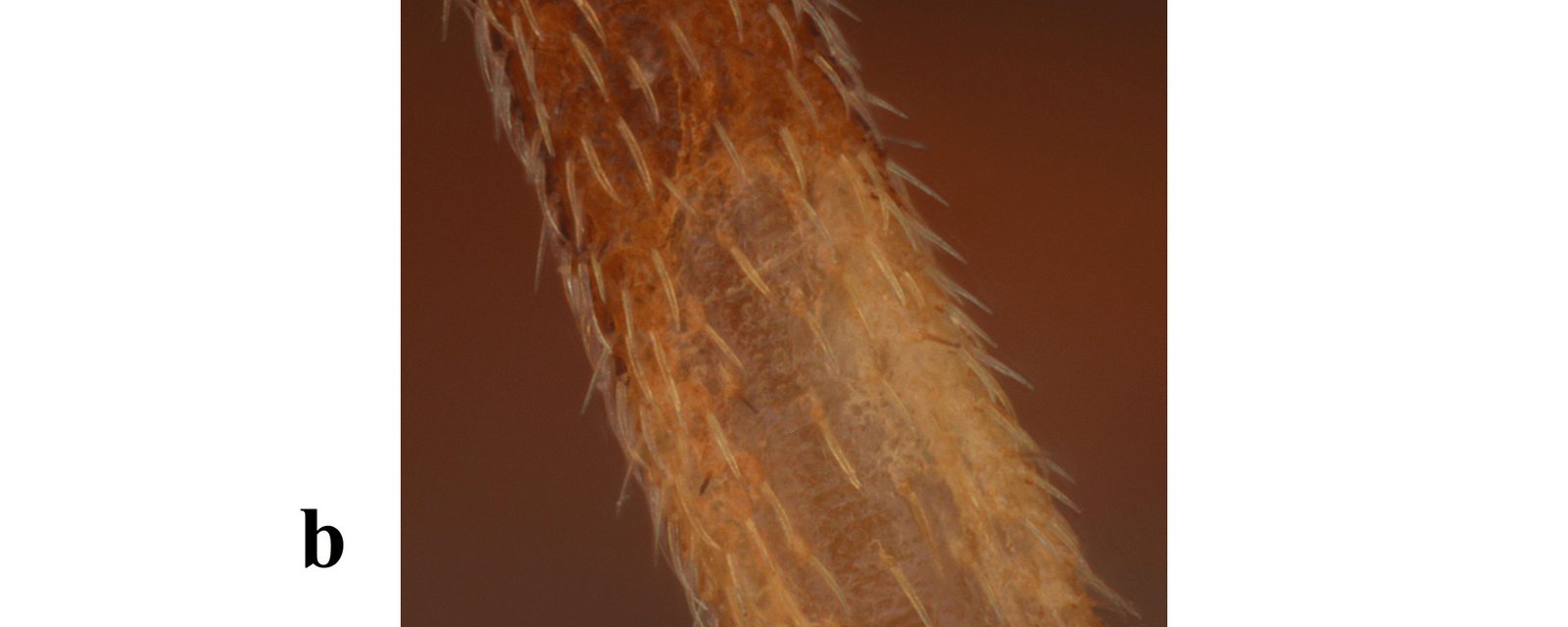		
5	Suprahumerals produced atop a thin and distinct columnar outgrowth (a)	**8**
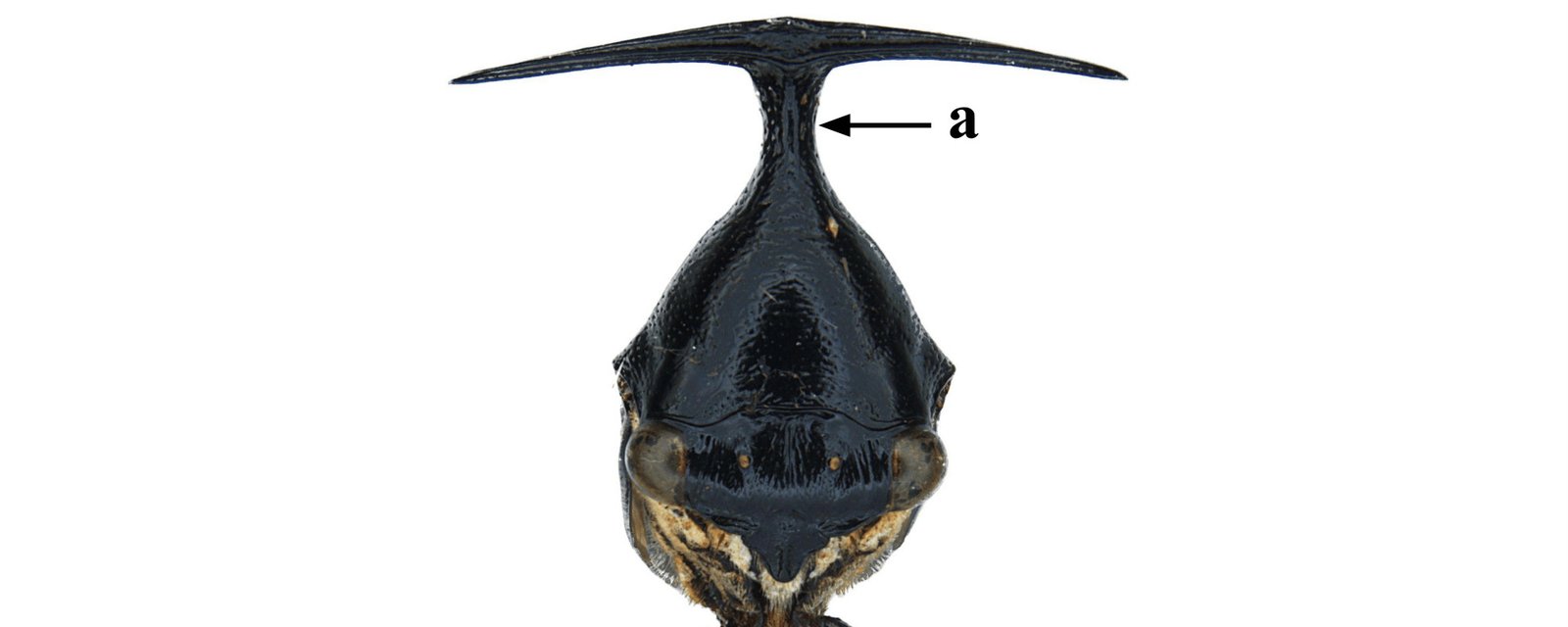		
–	Thin and distinct columnar outgrowth absent from pronotum (b)	**9**
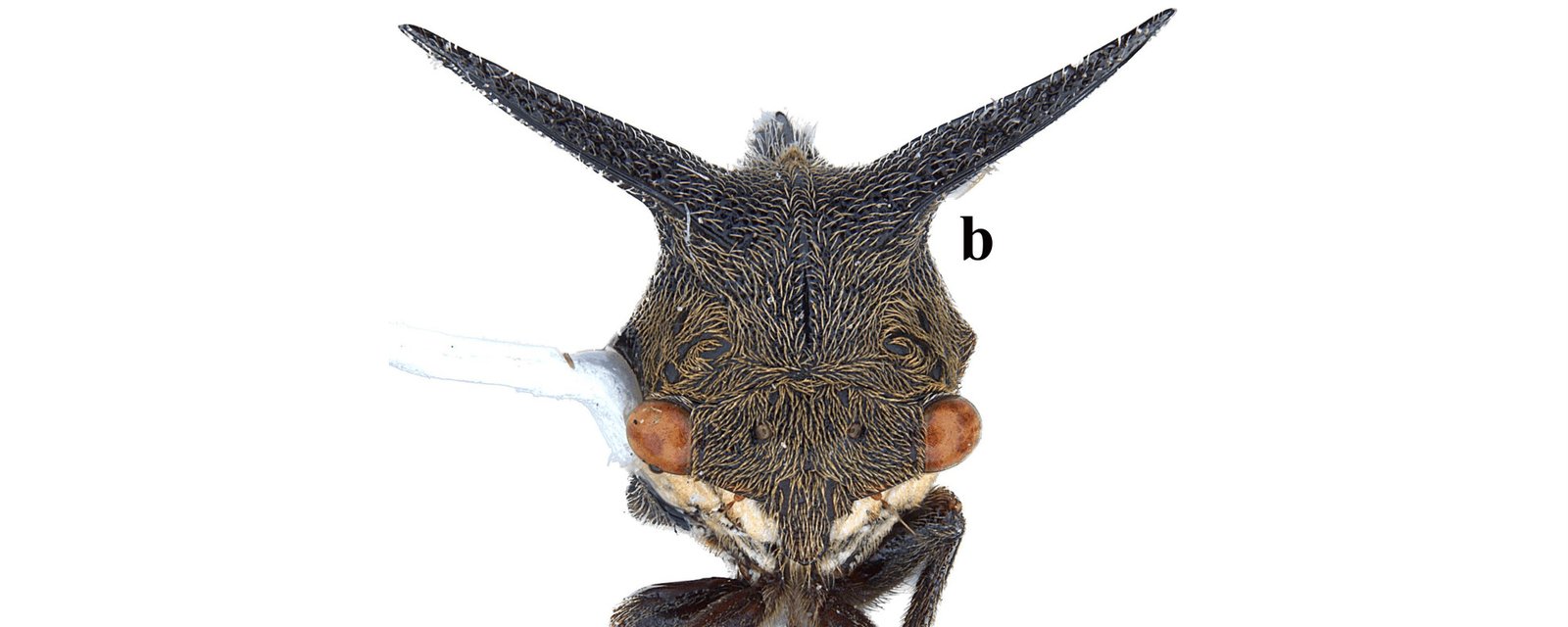		
6	Frontoclypeal margins expanding steeply towards apex (a)	**10**
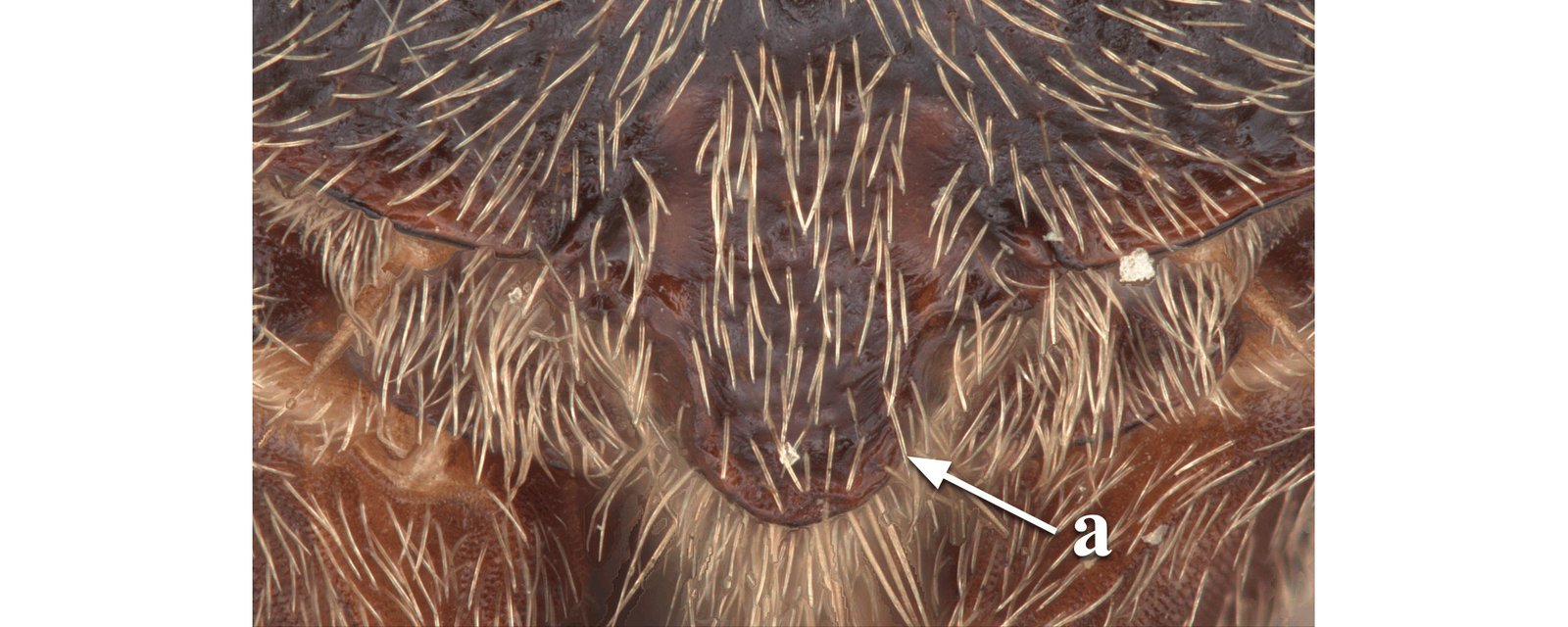		
–	Frontoclypeal margins converging towards apex (b)	**11**
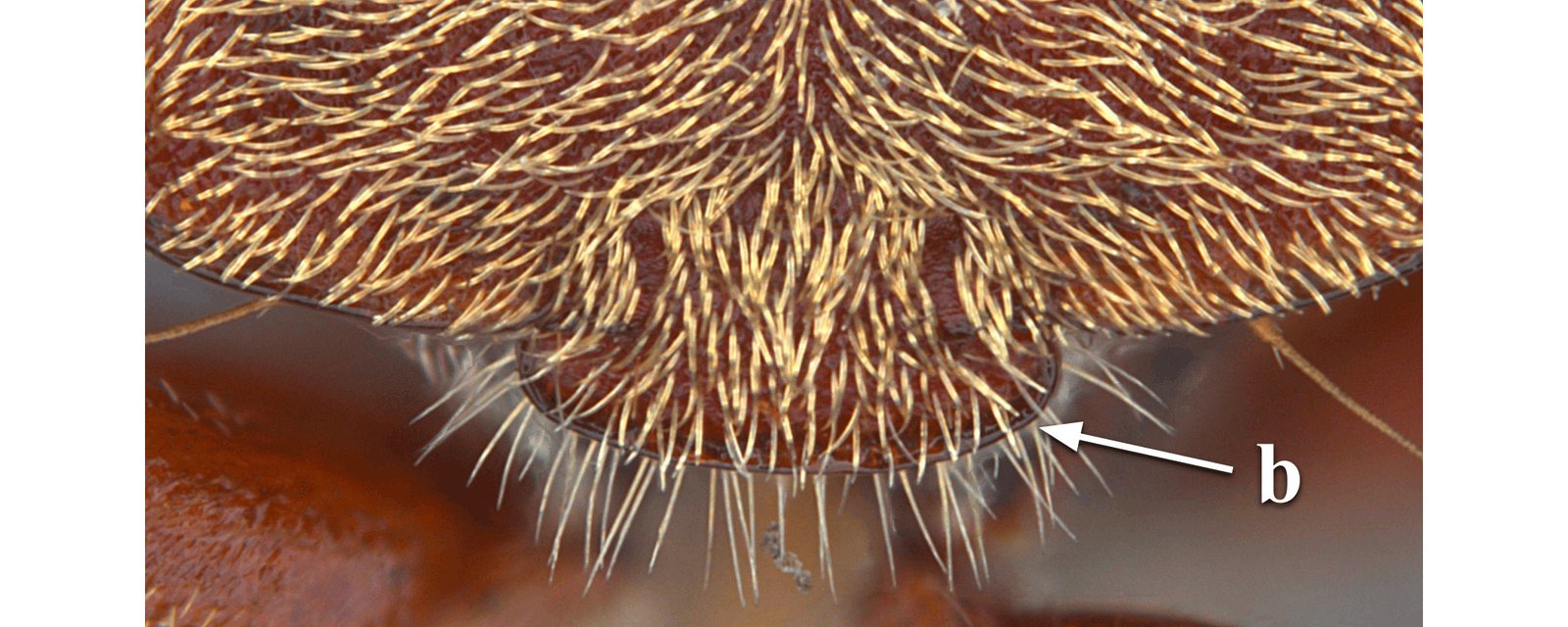		
7	Suprahumerals absent (a); hind wings with 1–3ac (3 apical cells) (b)	***Ebhul varius* (Walker, 1858)**
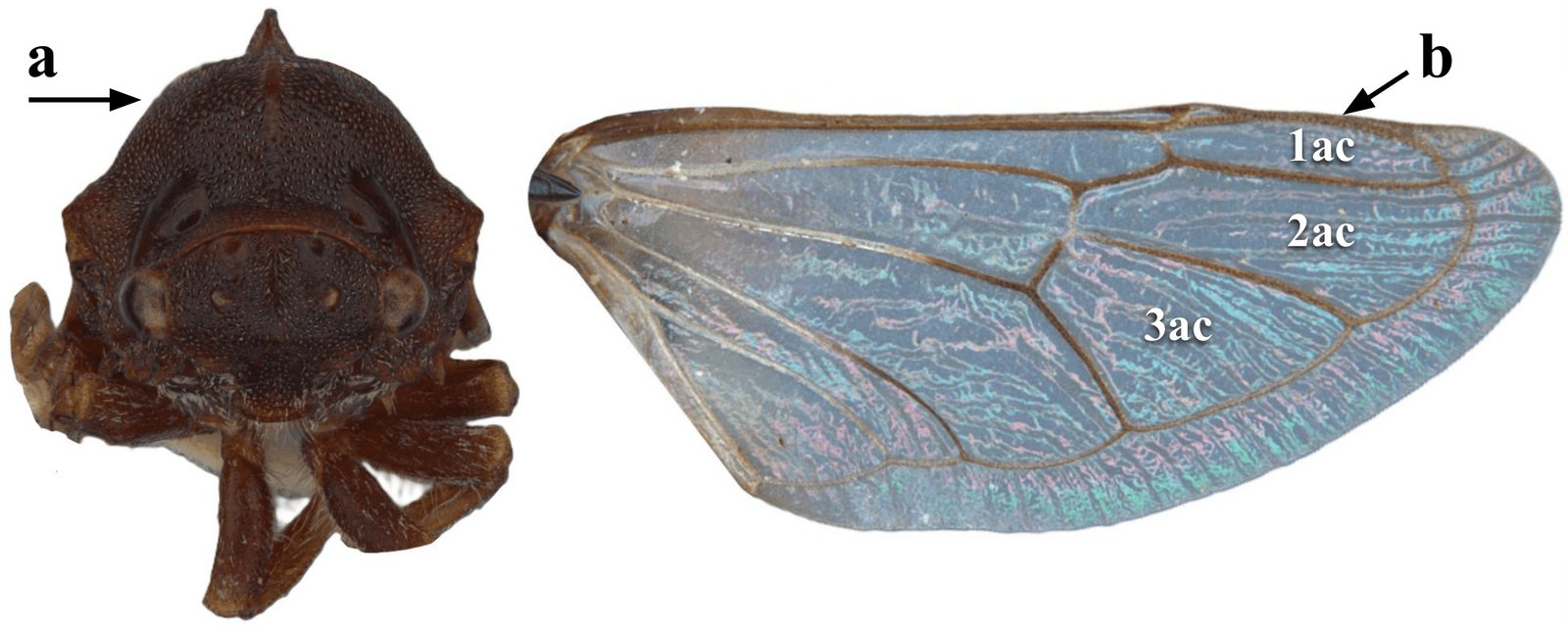		
–	Suprahumerals present (c); hind wings with 1–4ac (4 apical cells) (d)	**12 (*Evanchon* Goding, 1930)**
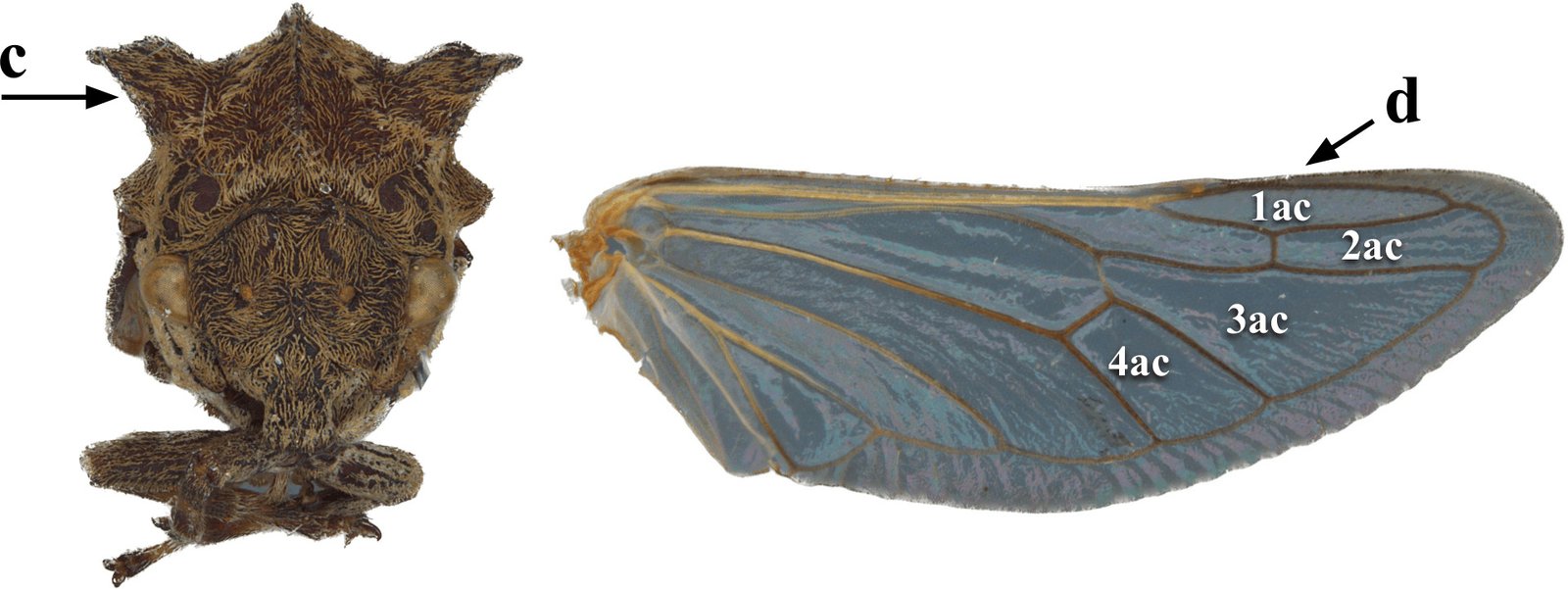		
8	Suprahumerals somewhat crescentiform from front view (a), point of origin of posterior process directly behind suprahumerals (b)	***Leptobelus aff. elevatus* Funkhouser, 1929** ♀
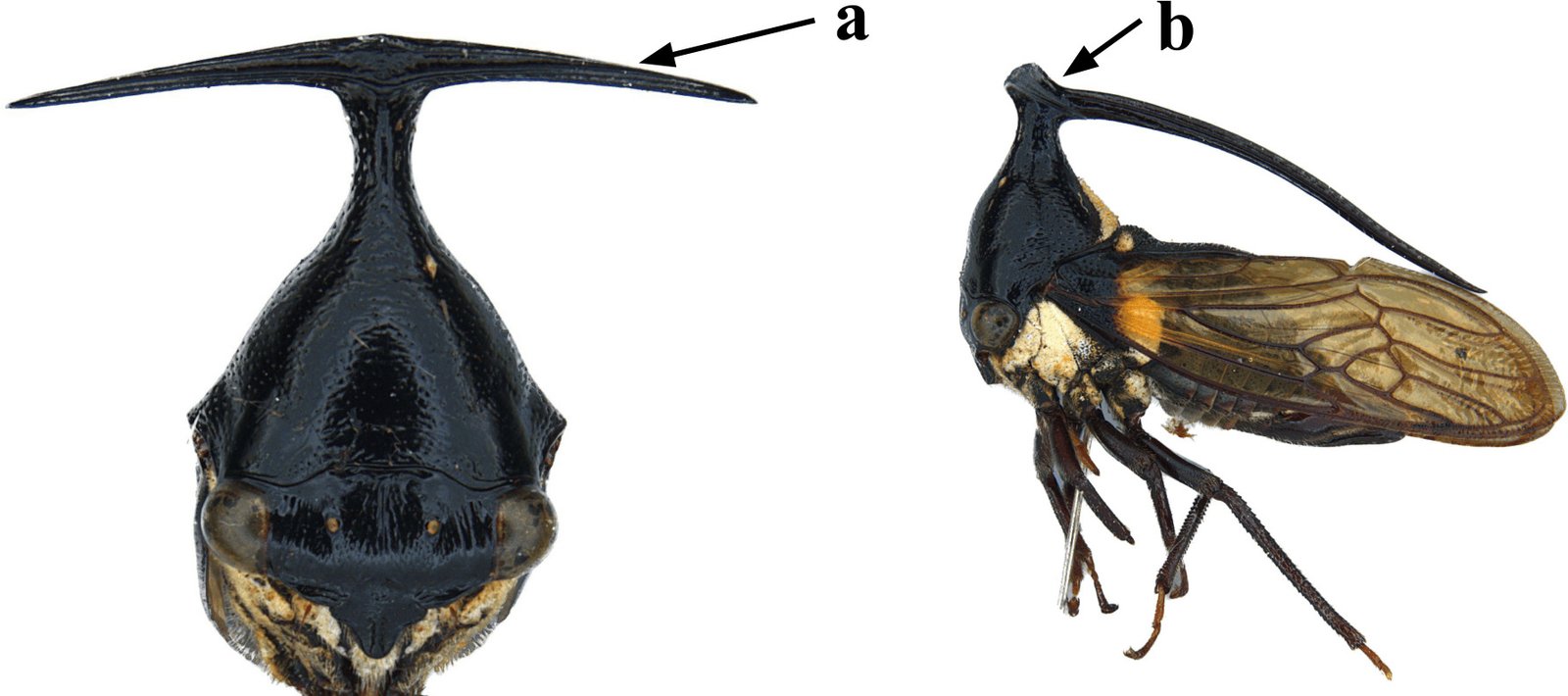		
–	Suprahumerals deeply forked from front view (c), point of origin of posterior process near the scutellum (d)	***Micreune formidanda* Walker, 1857**
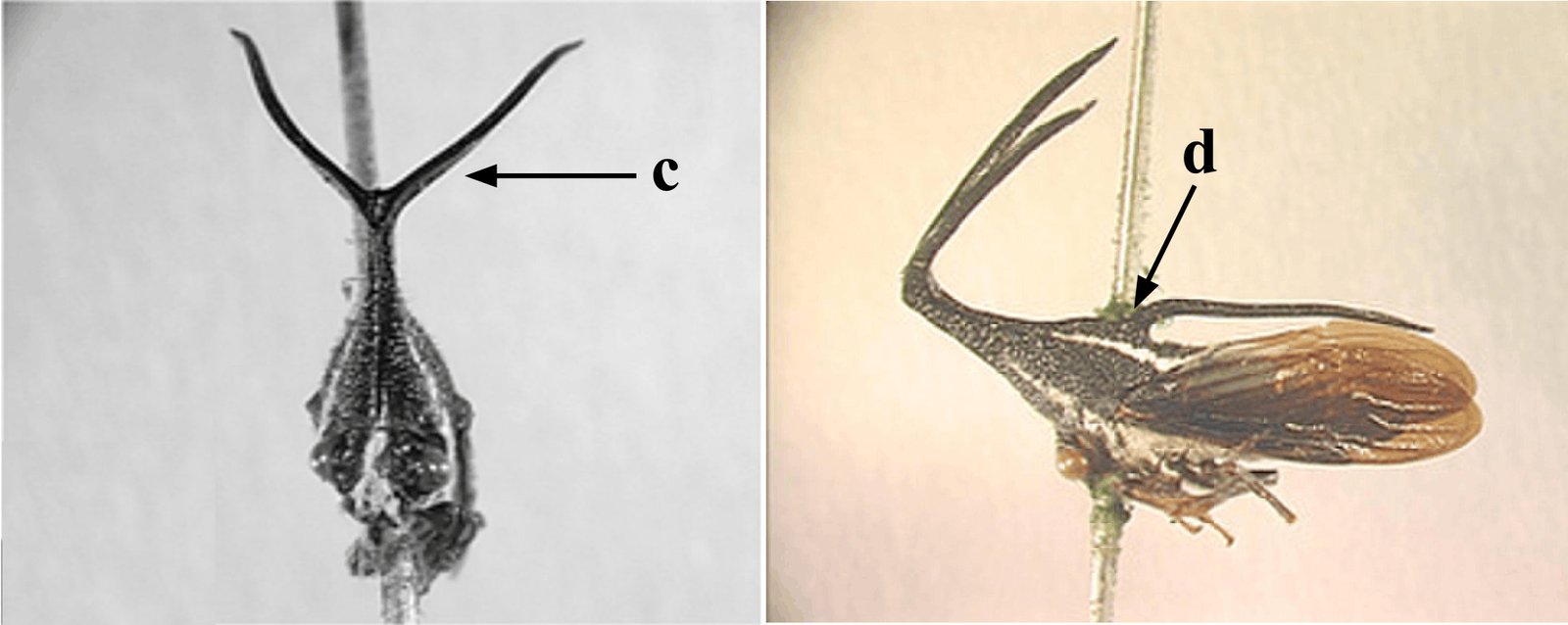		
9	Scutellum longer than wide (a); anterior tergal borders lined with distinct humps (b)	***Telingana recurvata* Distant, 1916**
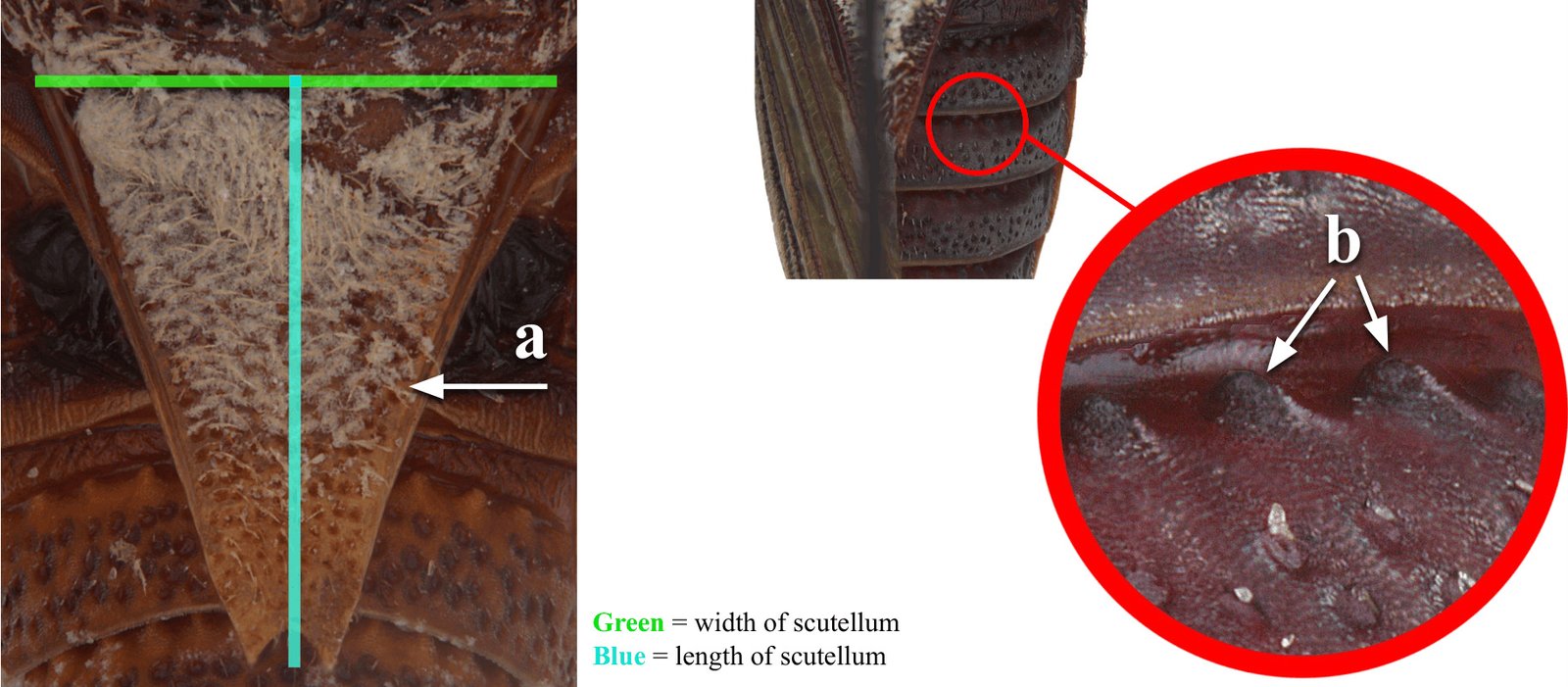		
–	Scutellum roughly as long as wide (c); anterior tergal borders generally straight without distinct protrusions (d)	**13**
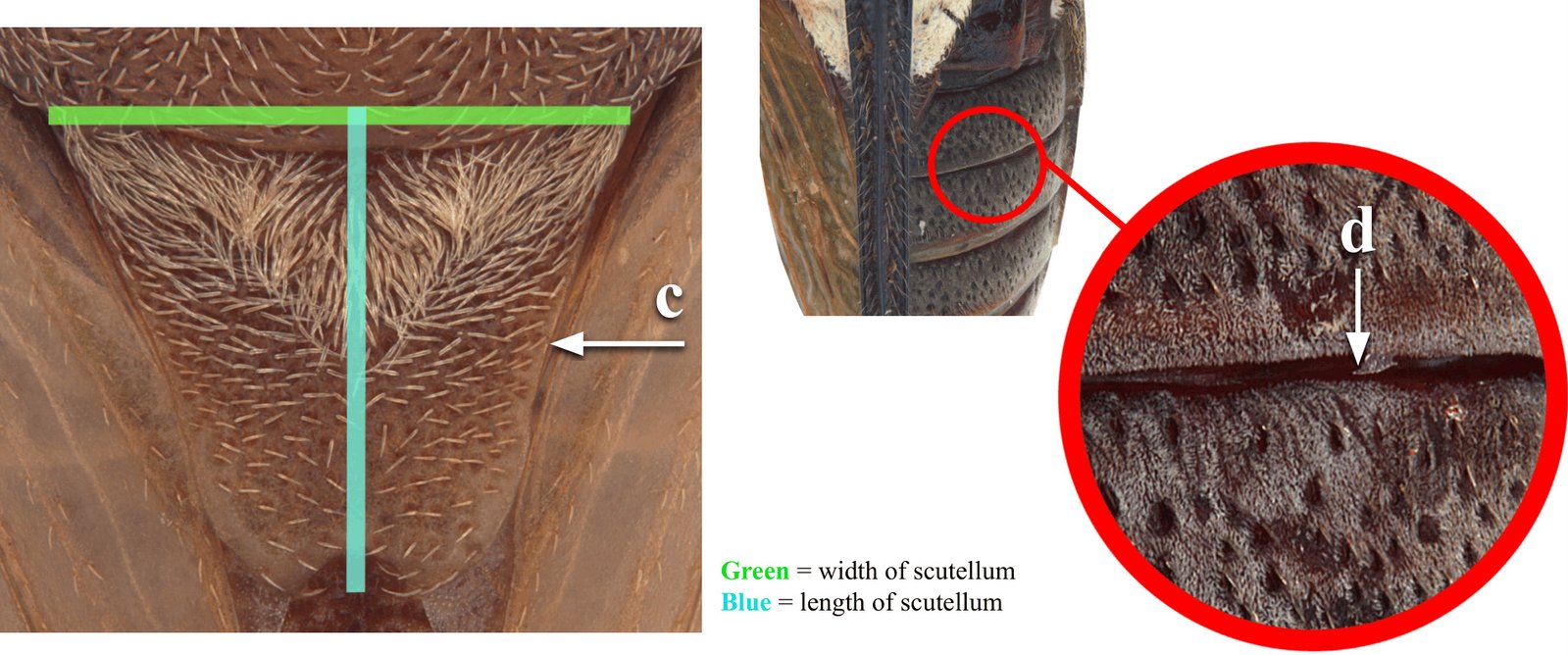		
10	Forewing opaque (a); pronotum densely covered with thick hairs (b)	***Ceraon orientale* Funkhouser, 1918**
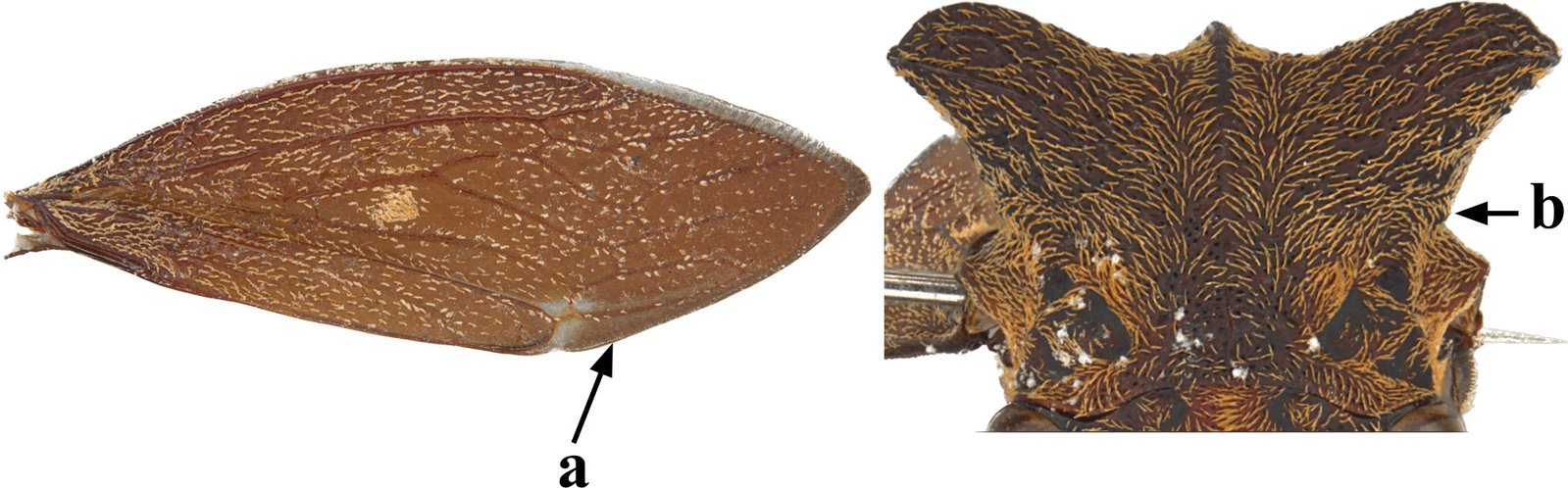		
–	Forewing translucent (c); pronotum sparsely haired (d)	**14 (*Centrotypus* Stål, 1866)**
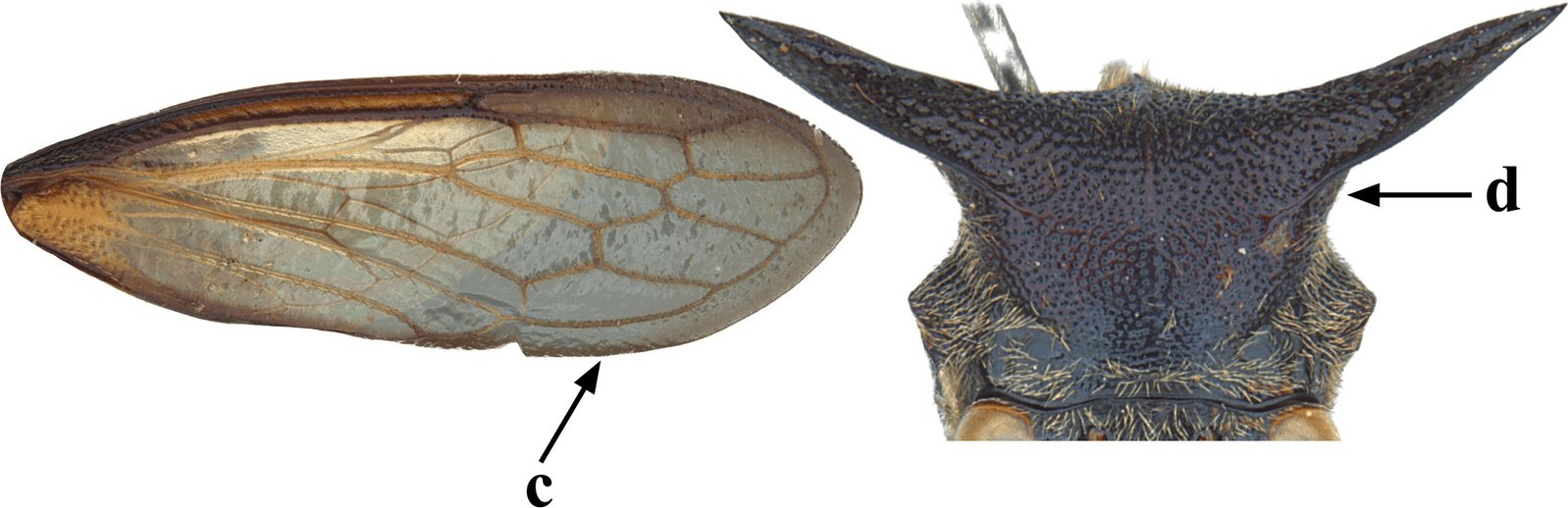		
11	Metathoracic trochanters unarmed (a)	**15**
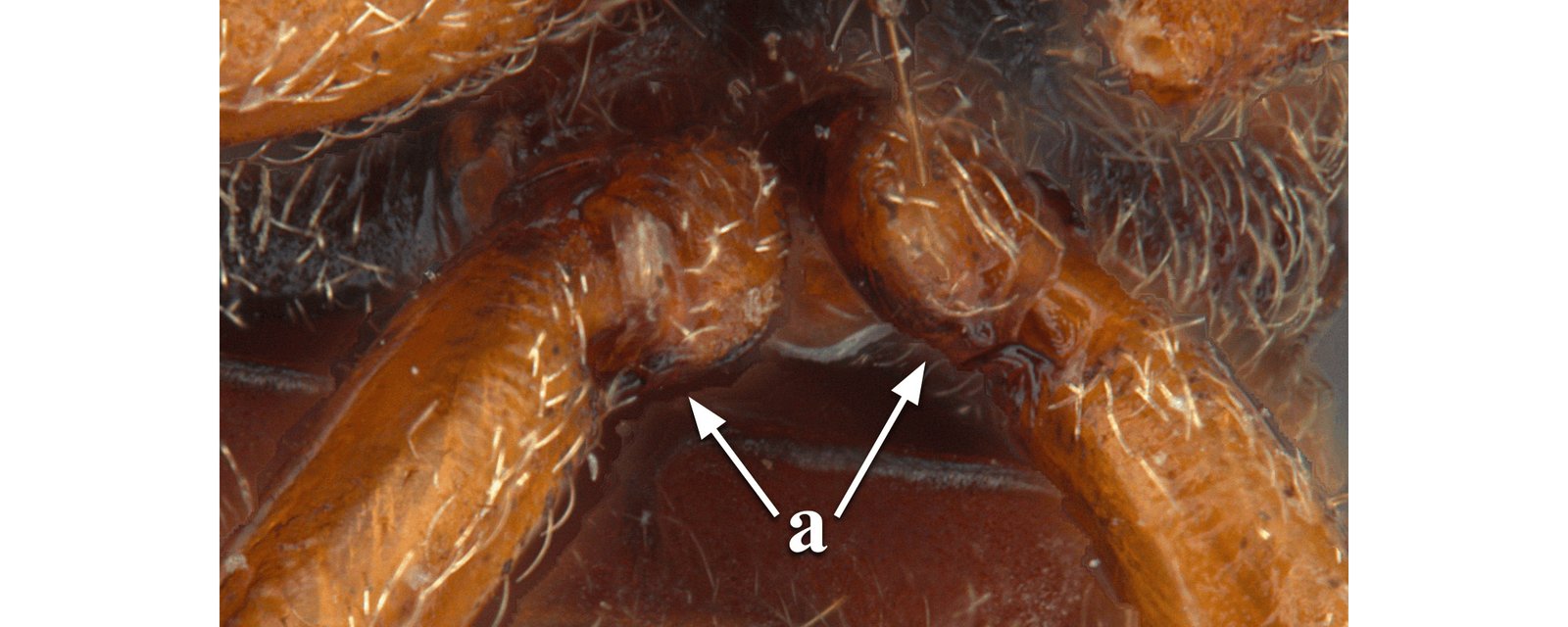		
–	Metathoracic trochanters armed (b)	**16**
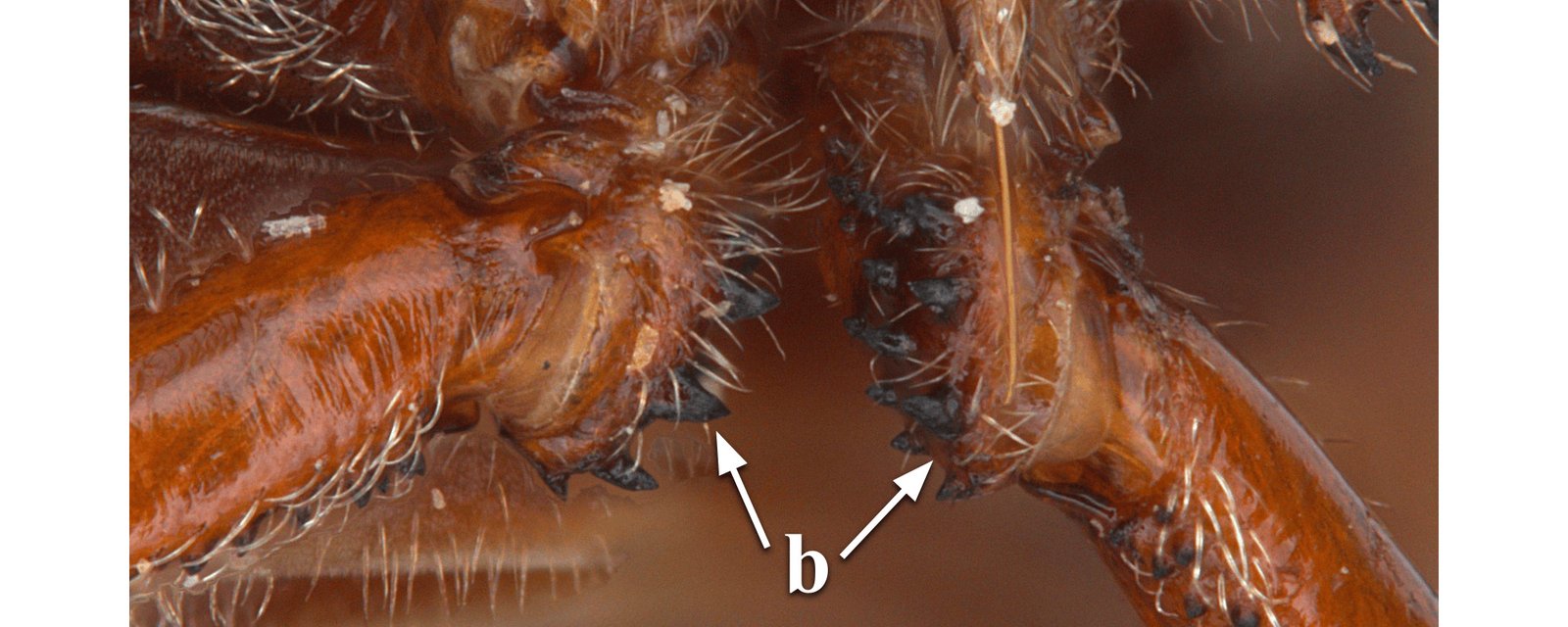		
12	Forewing with 1ac squarish (a), veins lined with small but prominent nodules (b)	***Evanchon variegatum* (Funkhouser, 1918)**
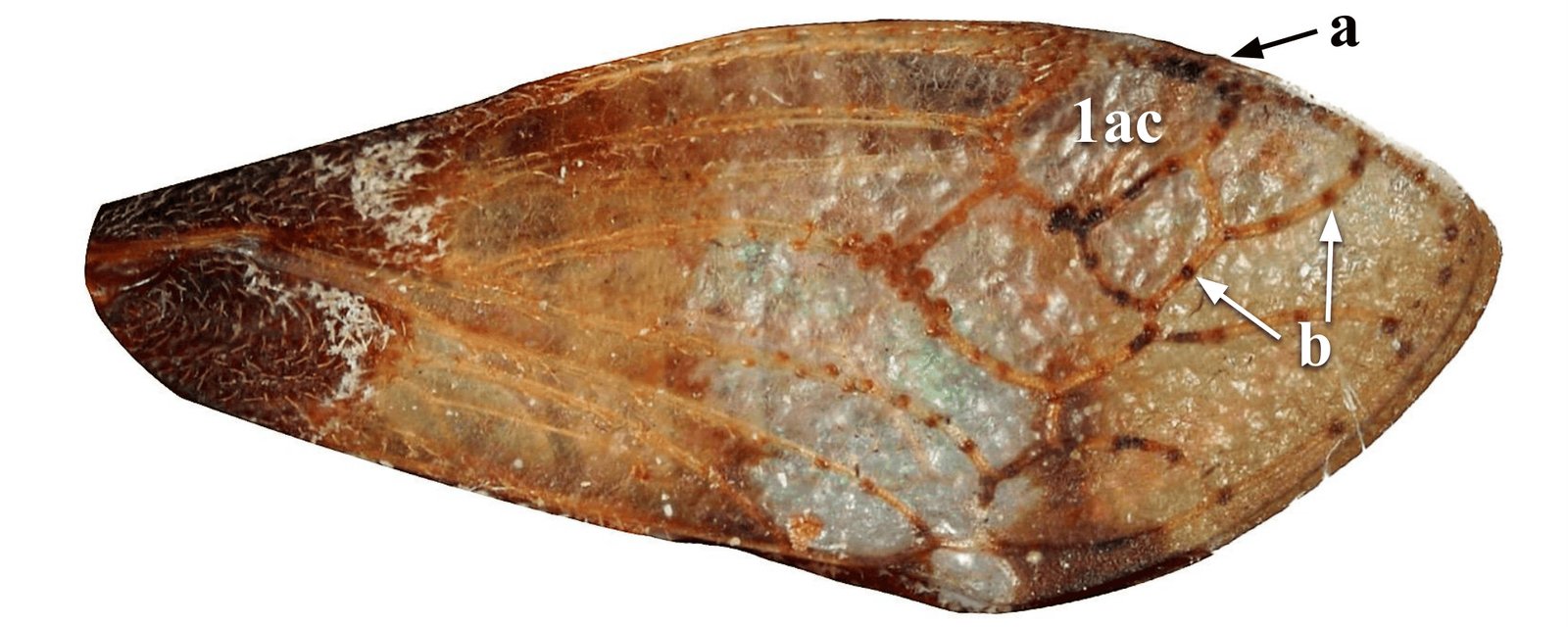		
–	Forewing with 1ac elongated (c), veins without prominent nodules (d)	***Evanchon maculatum* Funkhouser, 1937** ♀
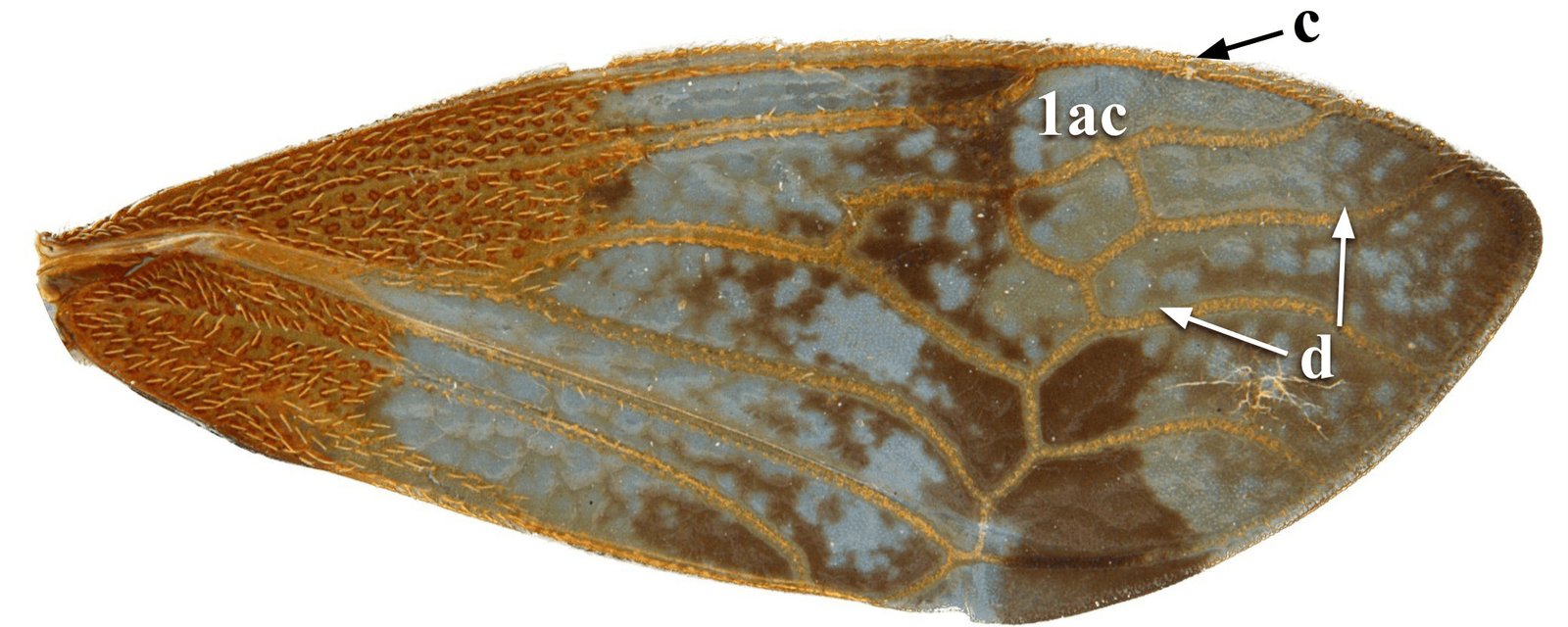		
13	Tip of posterior process not reaching 5ac of forewing (a)	***Nilautama minutispina* Funkhouser, 1918**
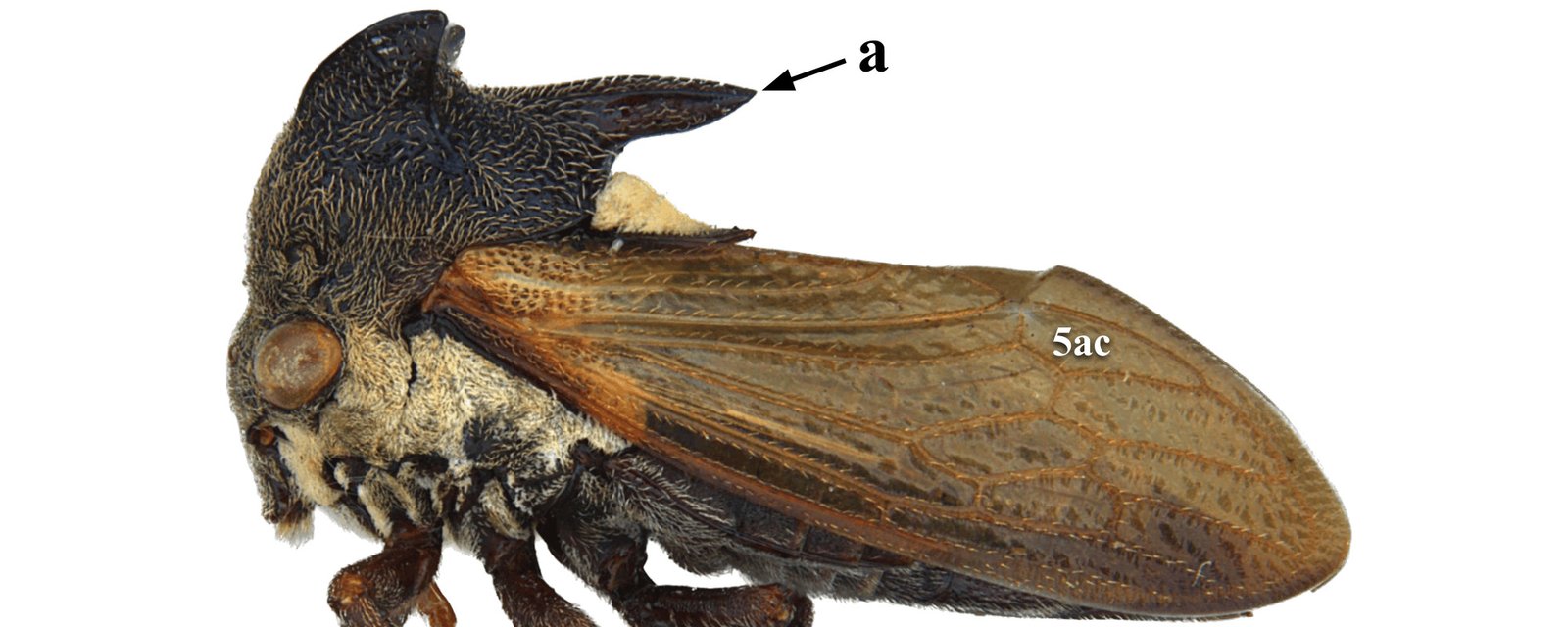		
–	Tip of posterior process reaching or surpassing 5ac of forewing (b)	**17 (*Leptocentrus* Stål, 1866)**
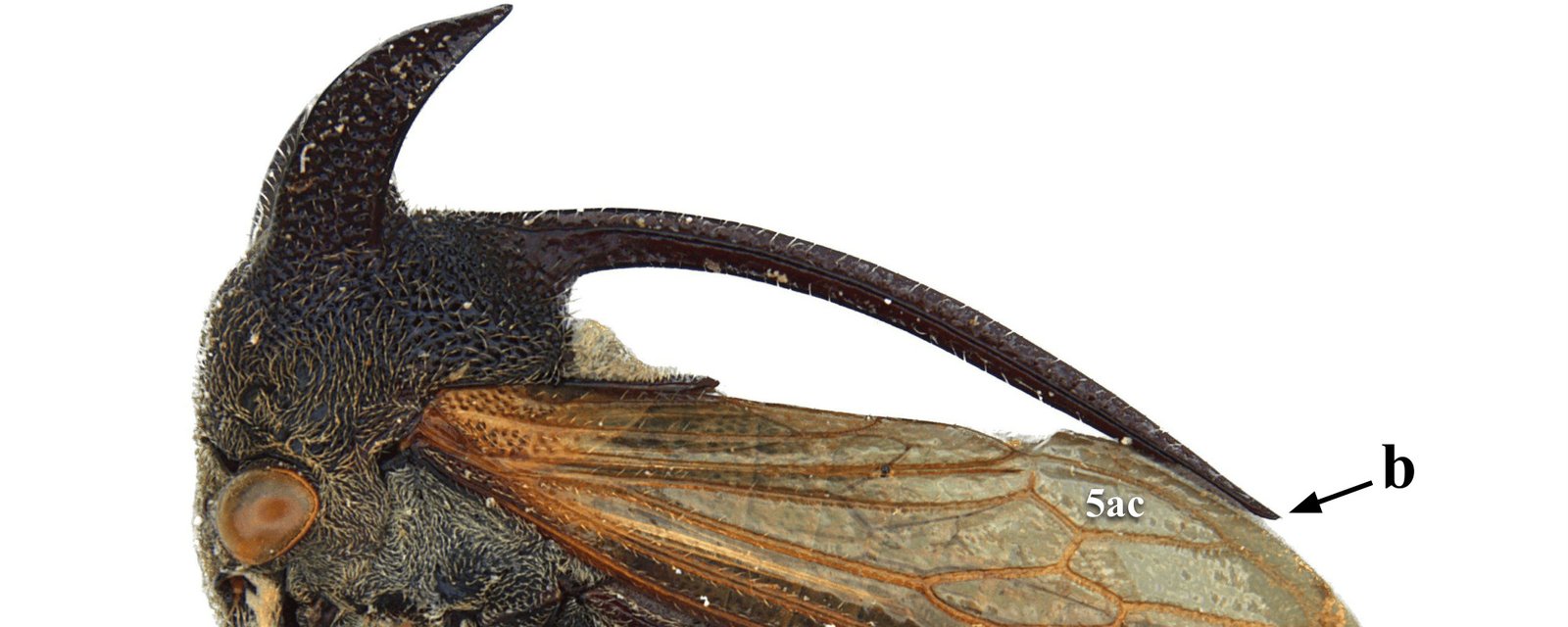		
14	Forewing with 1dc distinctly darkened (a)	**18**
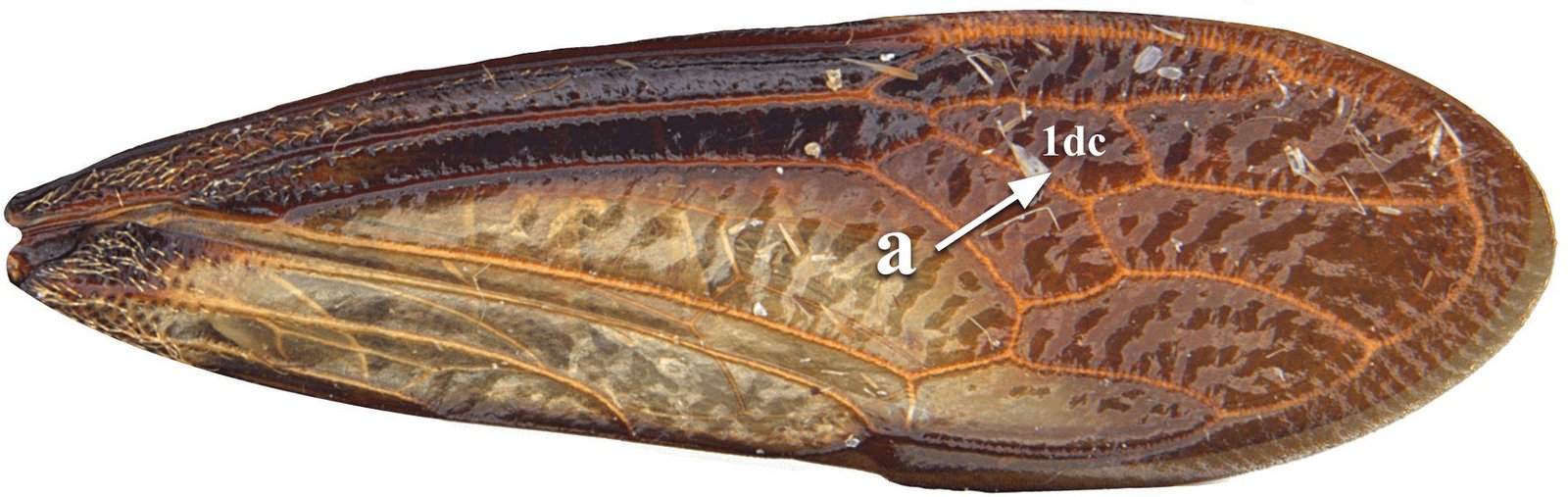		
–	Forewing with 1dc not darkened (b)	**19**
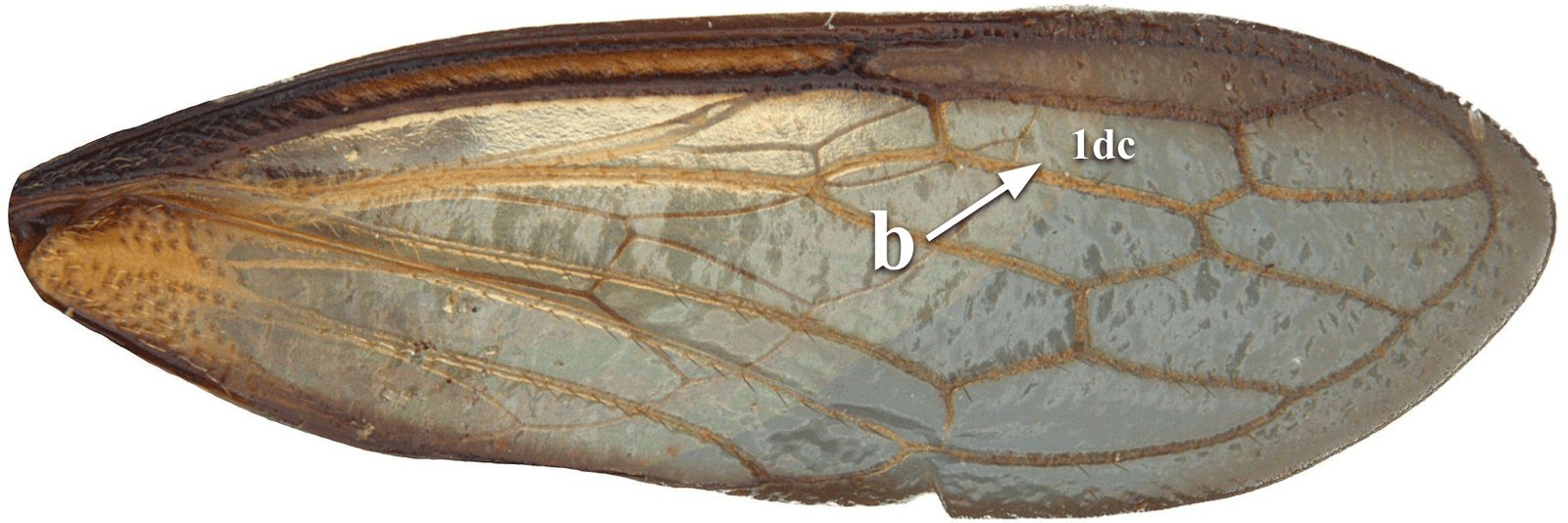		
15	Suprahumerals present (a)	**20**
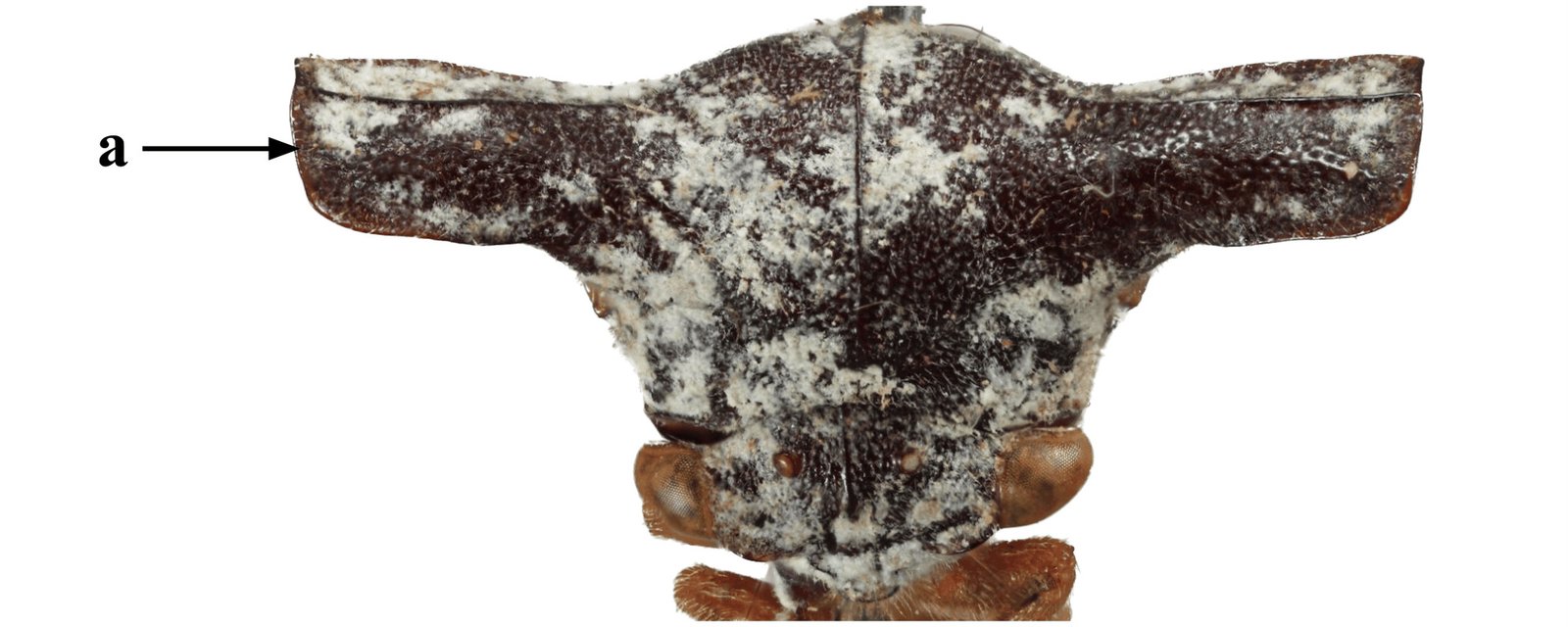		
–	Suprahumerals absent (b)	**21**
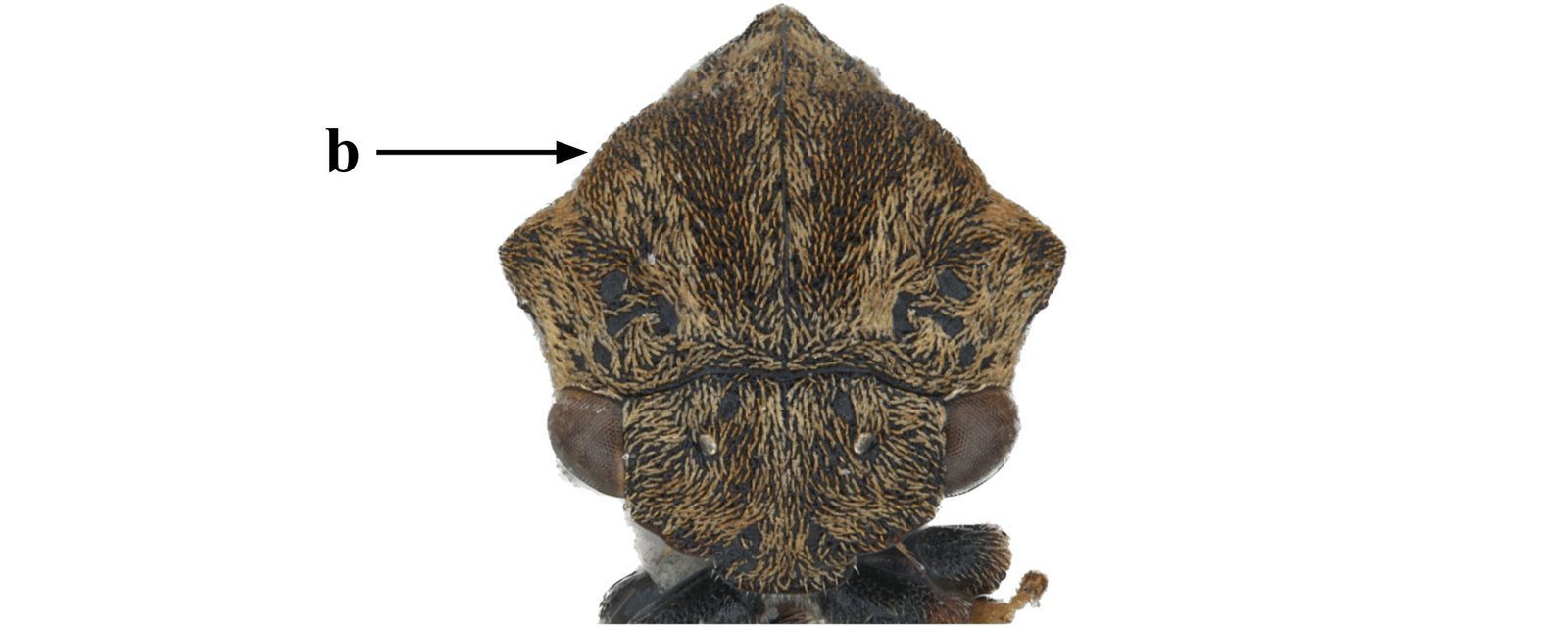		
16	Humeral angles auriculate (a)	**22**
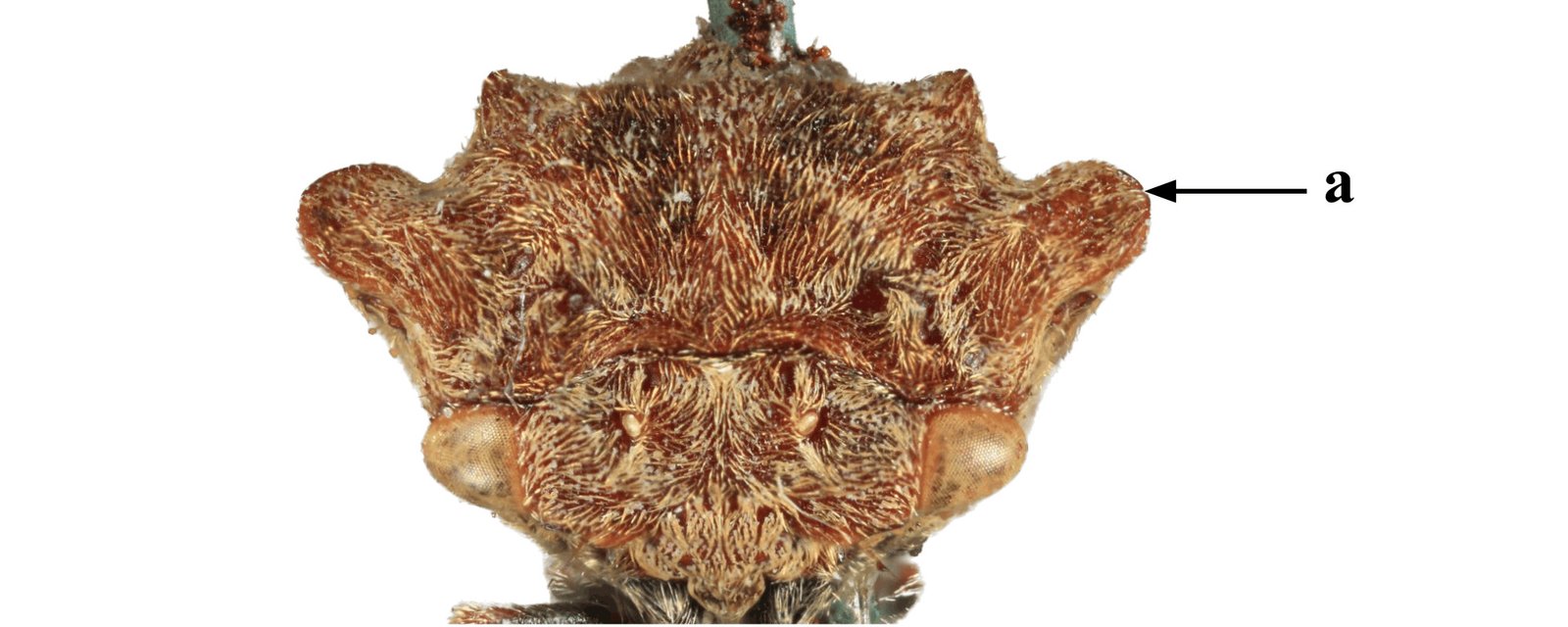		
–	Humeral angles acuminate (b) or inconspicuous (c)	**23 (*Tricentrus* Stål, 1866)**
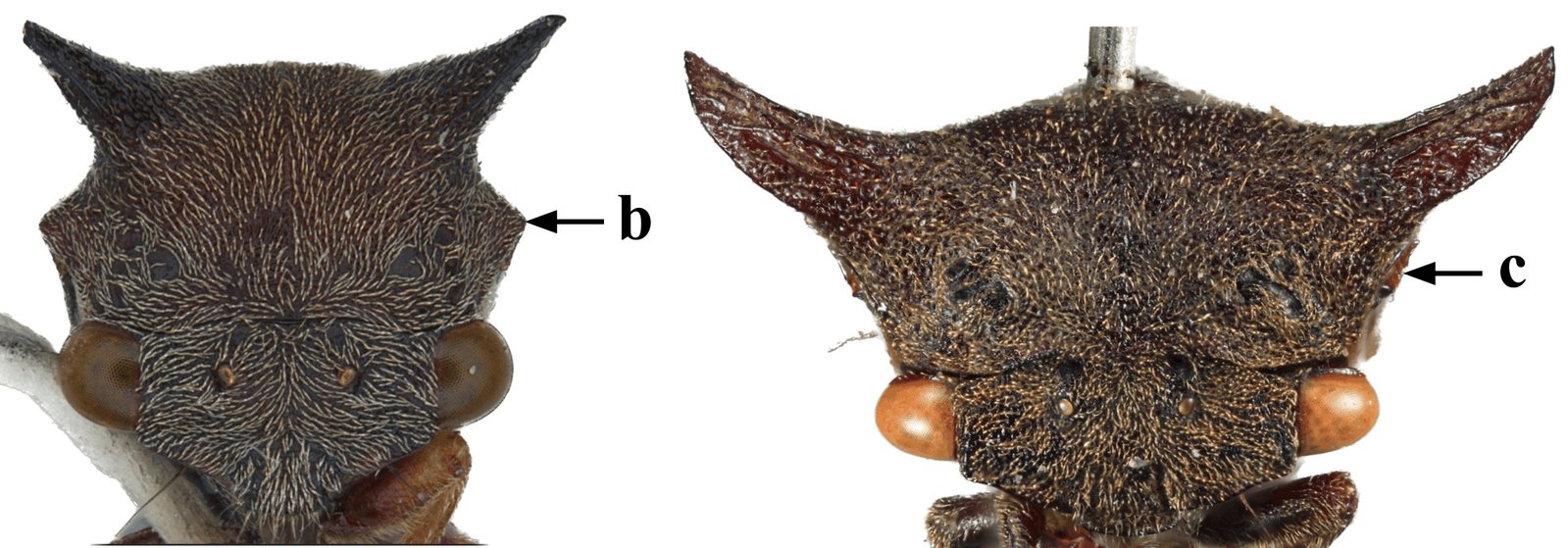		
17	Punctate base of forewing darkened (a); suprahumerals produced sub-horizontally from front view (b); eyes dull ochraceous (c)	***Leptocentrus taurus* (Fabricius, 1775)**
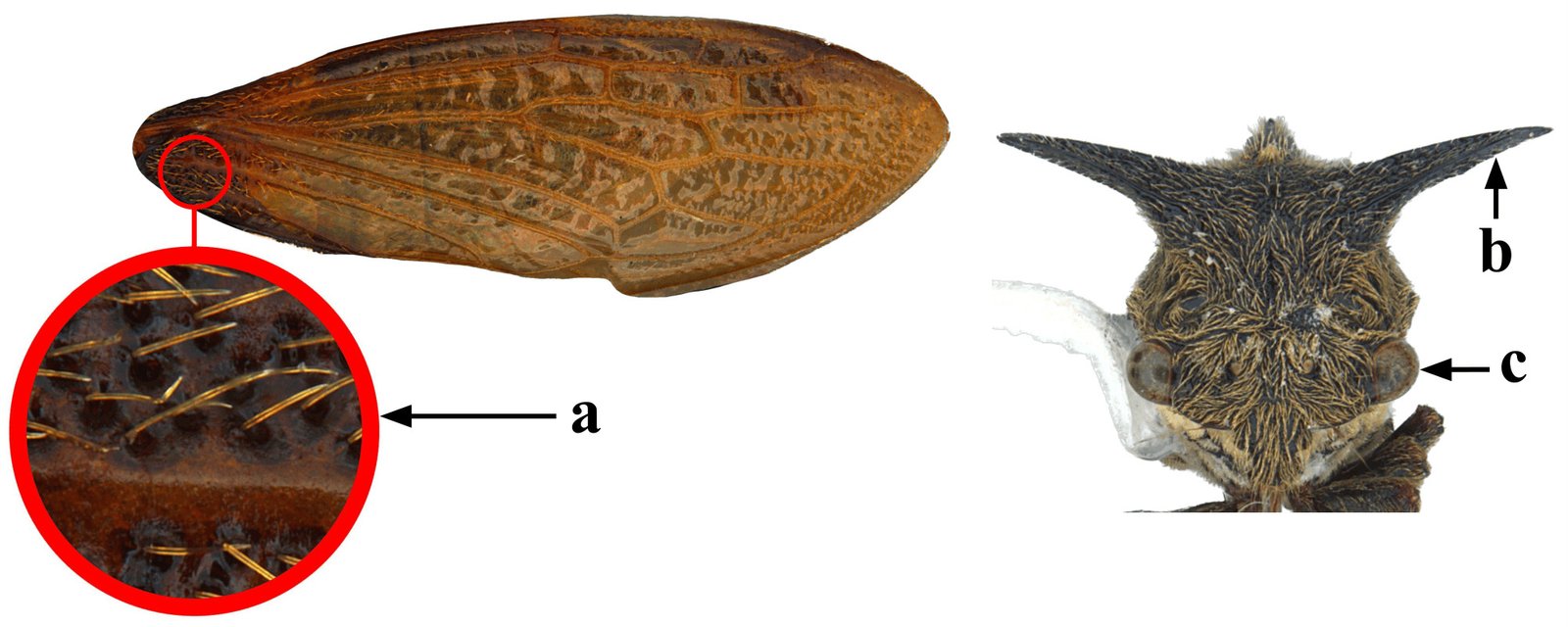		
–	Punctate base of forewing pale (d); suprahumerals produced obliquely from front view (e); eyes orange (f)	***Leptocentrus* sp**. ♀
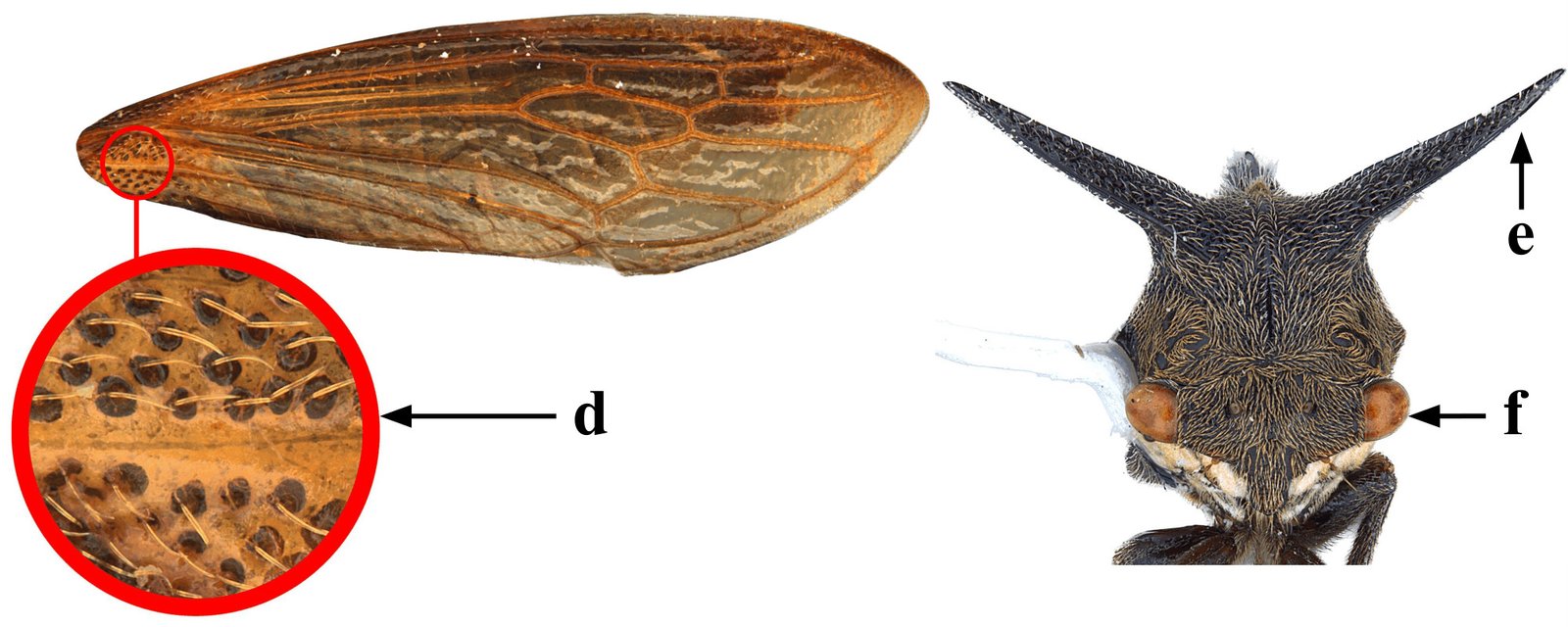		
18	Frontoclypeal lobes present, prominent (a)	***Centrotypus belus* (Buckton, 1903)**
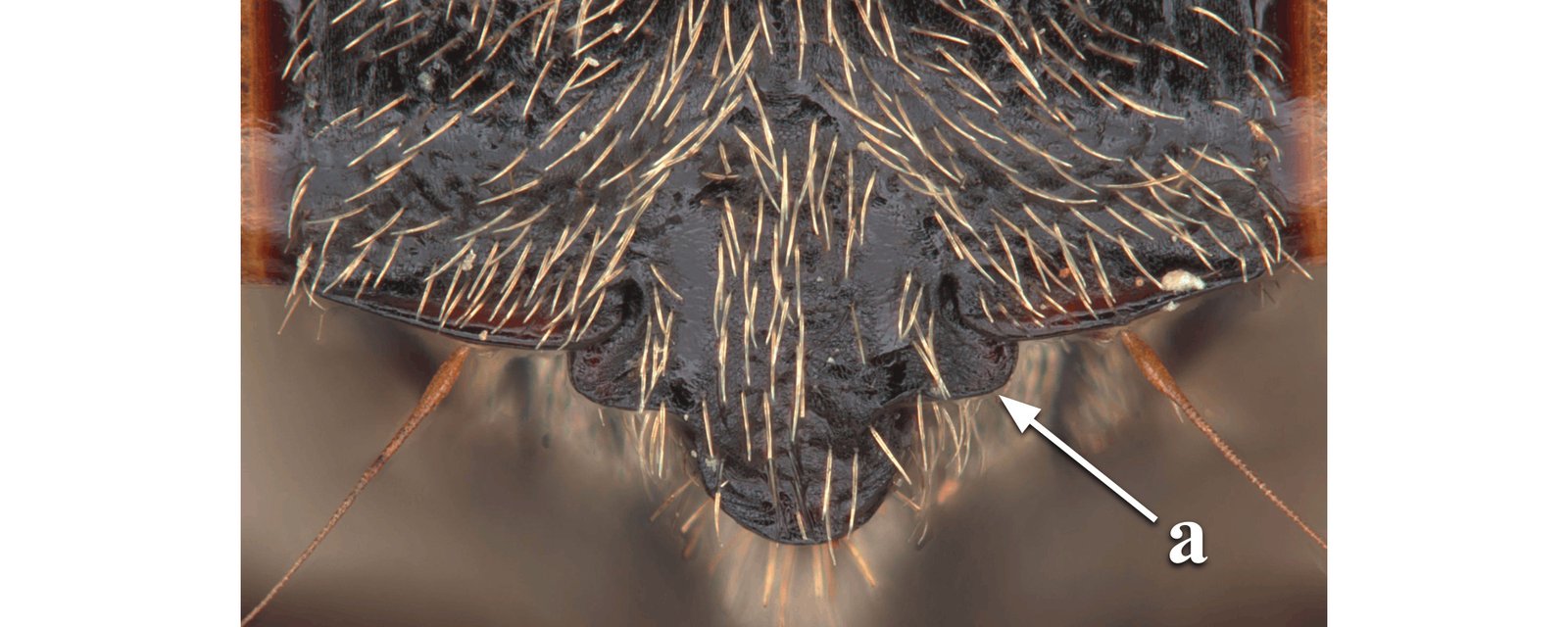		
–	Frontoclypeal lobes absent (b)	***Centrotypus* sp. 2** ♀
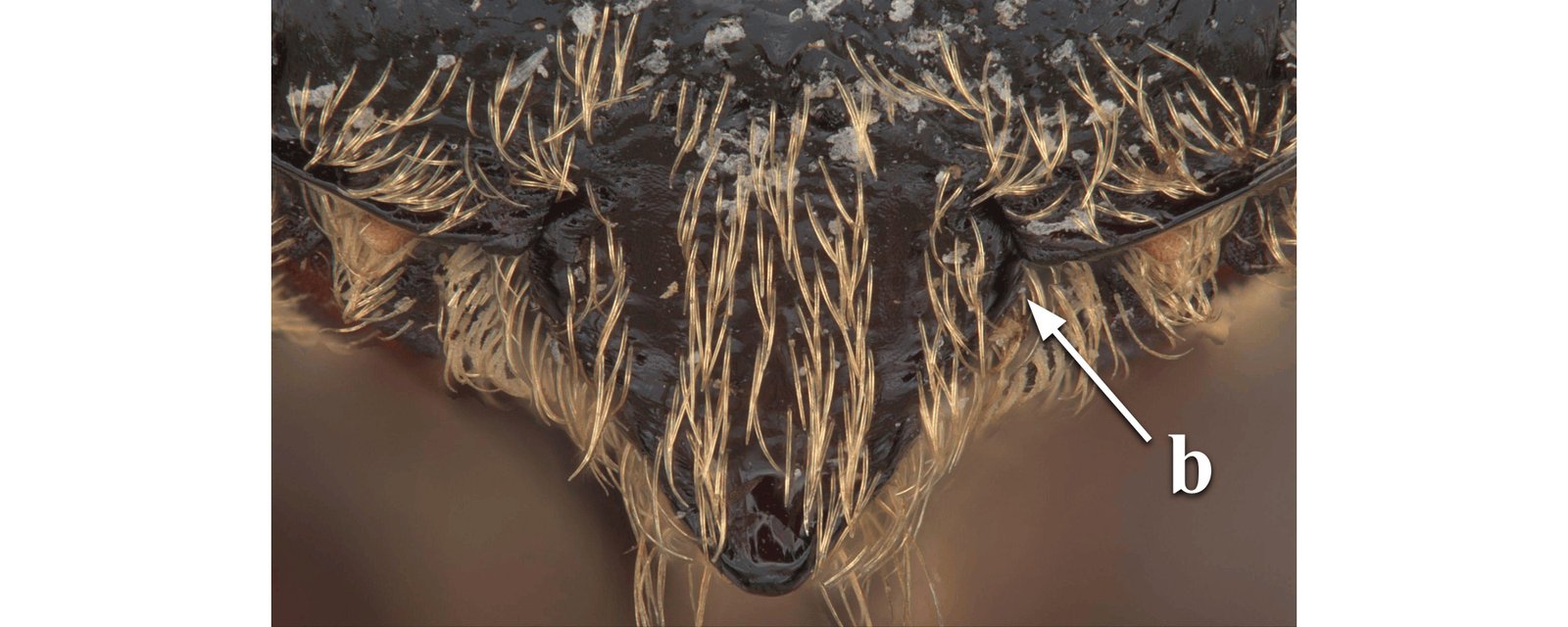		
19	R and R1 without any distinct darkening (a), forewing lurid (b)	***Centrotypus laminifer* (Walker, 1857)** ♀
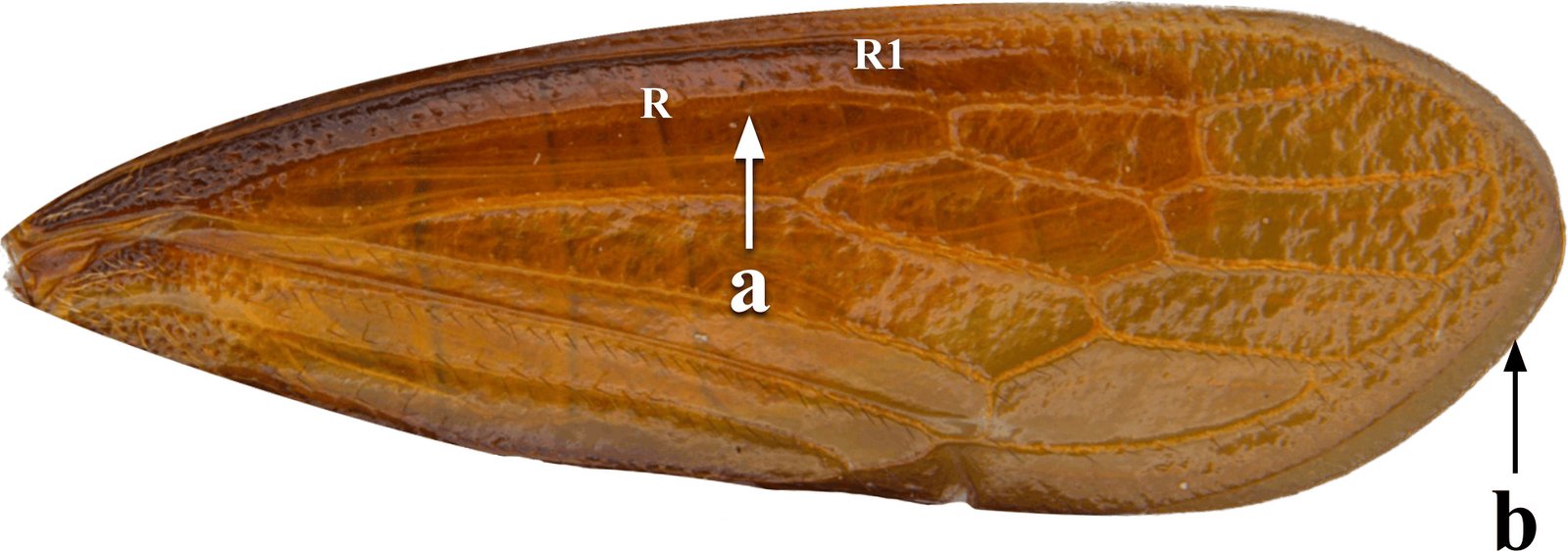		
–	R and R1 distinctly darkened (c), forewing clear hyaline (d)	***Centrotypus* sp. 1** ♀
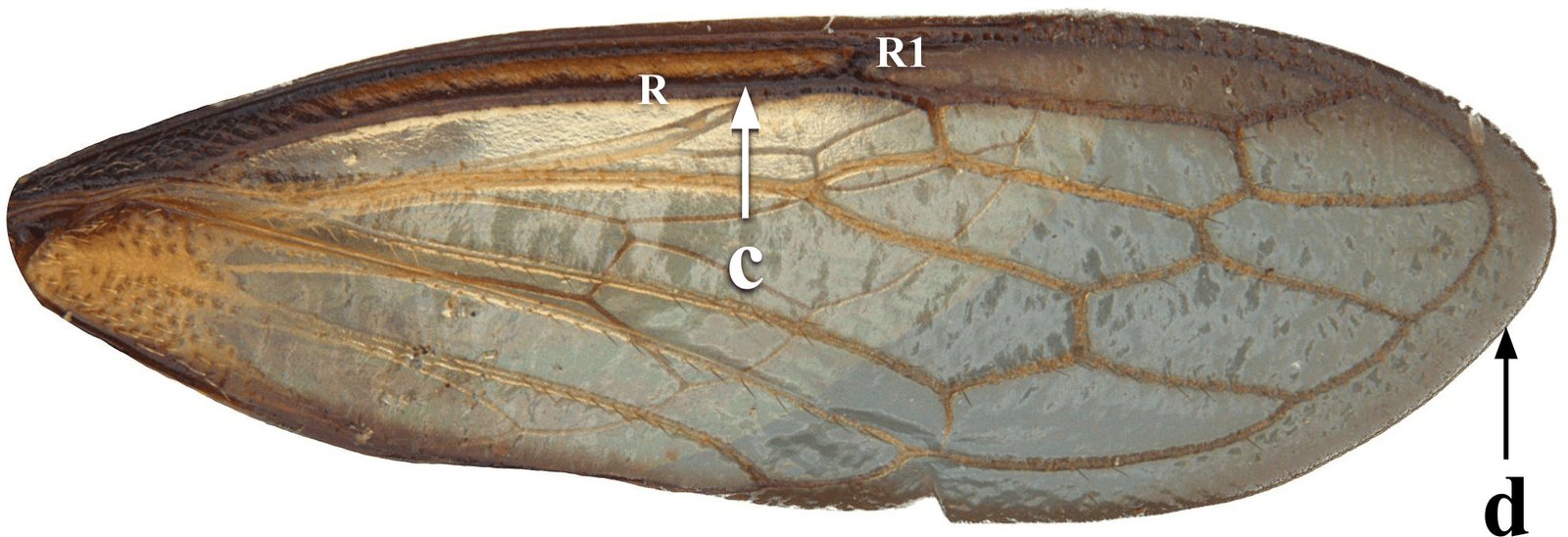		
20	Suprahumerals truncate from front view (a), humeral angles inconspicuous (b), posterior process generally straight with tip not reaching 5ac of forewing (c)	***Centruchus laticornis* Funkouser, 1918** ♀
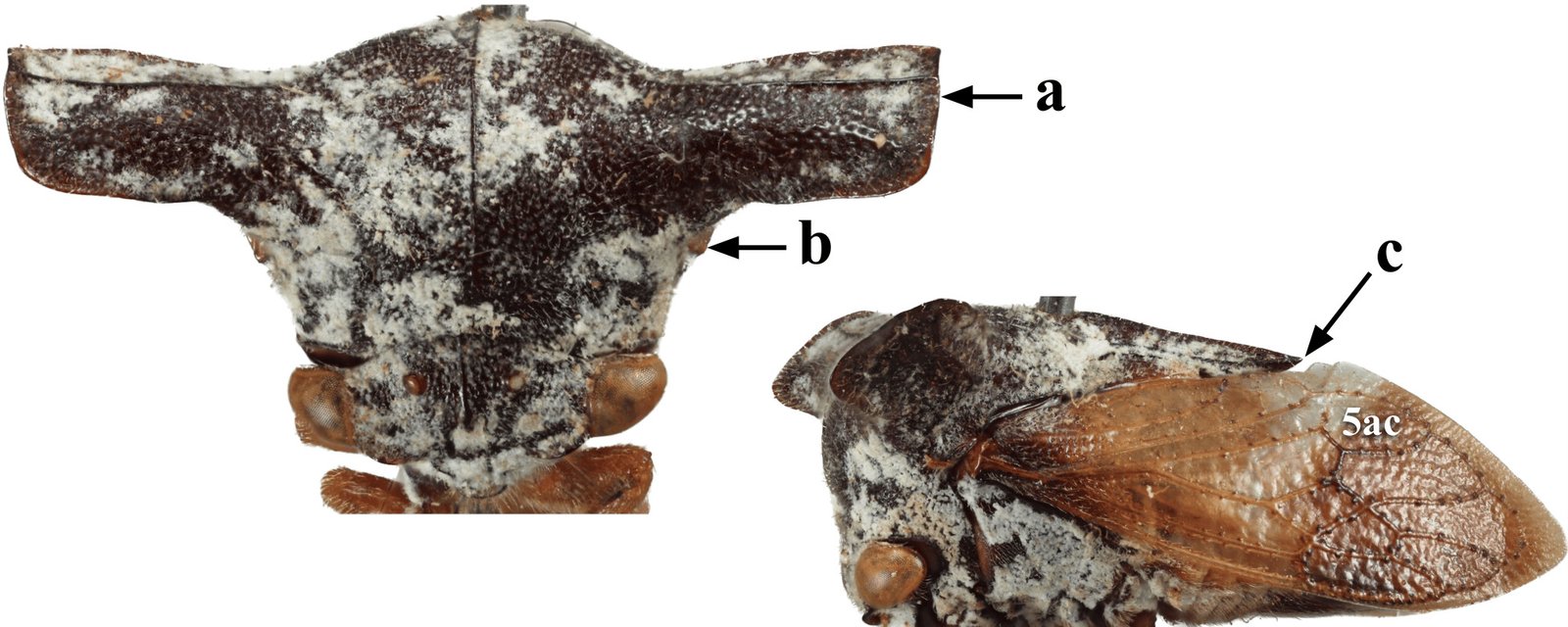		
–	Suprahumerals dentate and acuminate from front view (d), humeral angles prominent and acuminate (e), posterior process possessing distinct arcuate dorsal node (f) with tip reaching 5ac of forewing (g)	***Pantaleon bufo* (Kato, 1928)** ♀
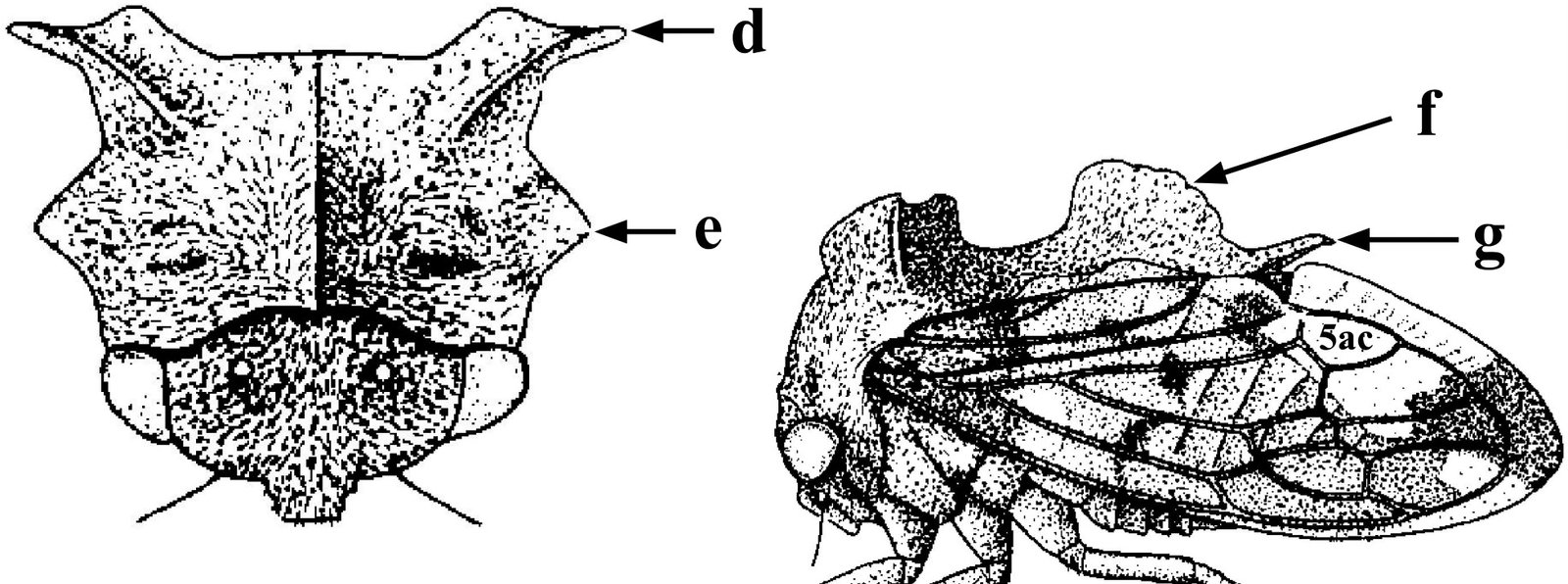		
21	Scutellum entirely concealed by posterior process and not visible from lateral view (a)	***Cryptaspidia piceola* (Melichar, 1903)**
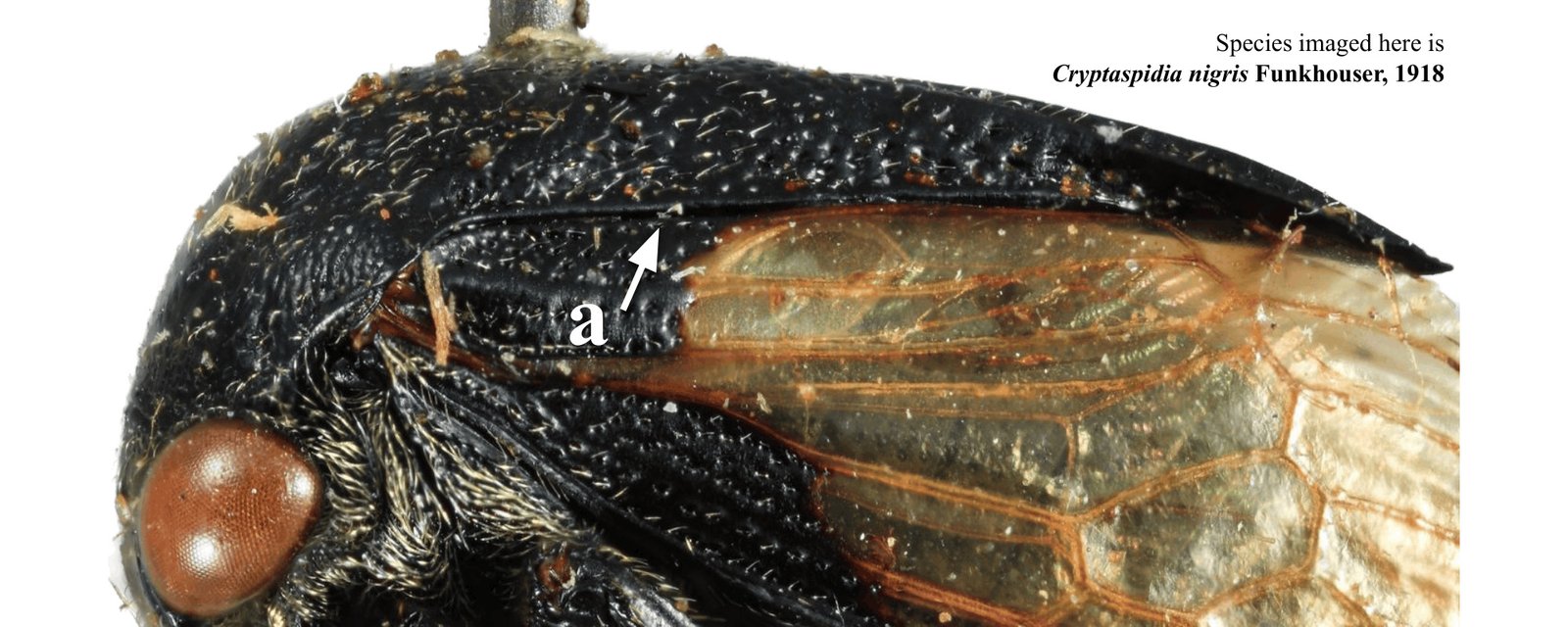		
–	Scutellum partially concealed by posterior process and visible from lateral view (b)	**24 (*Gargara* Amyot & Serville, 1843)**
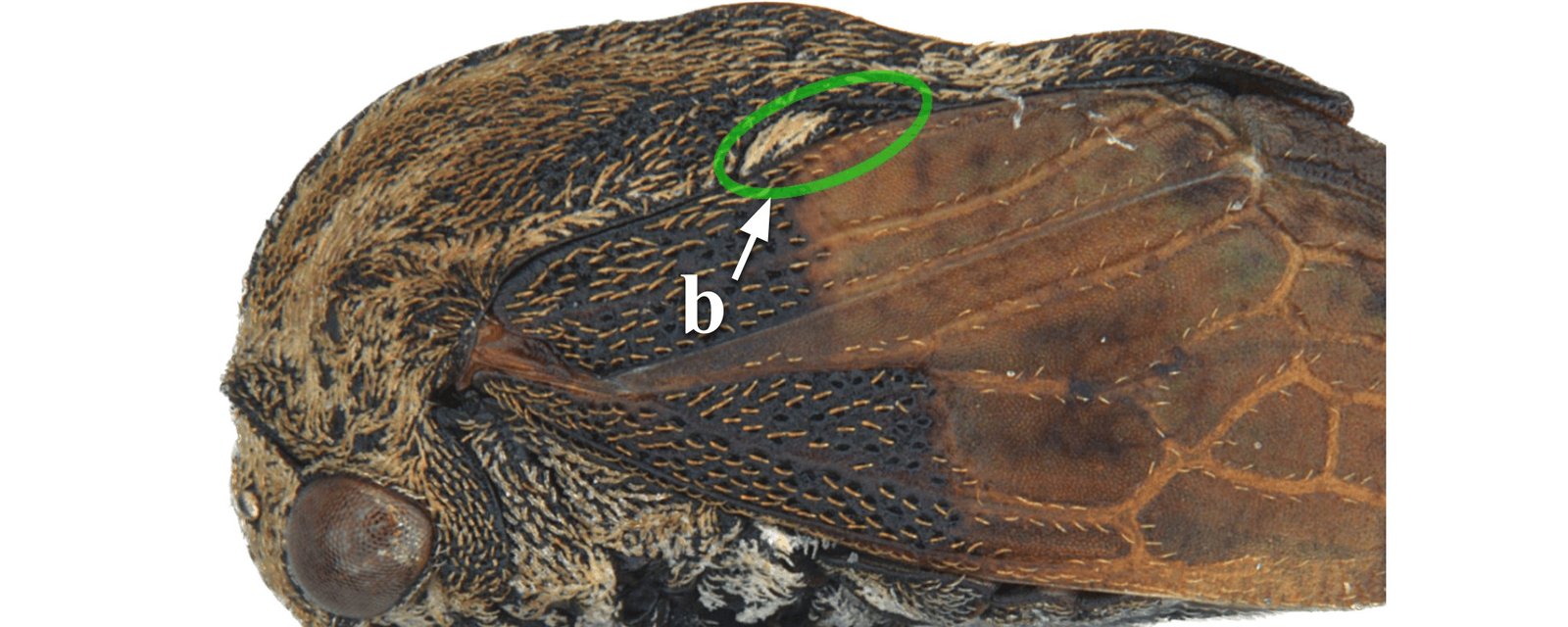		
22	Two prominent tubercles present between humeral angles (a)	***Sipylus dilatatus* (Walker, 1851)**
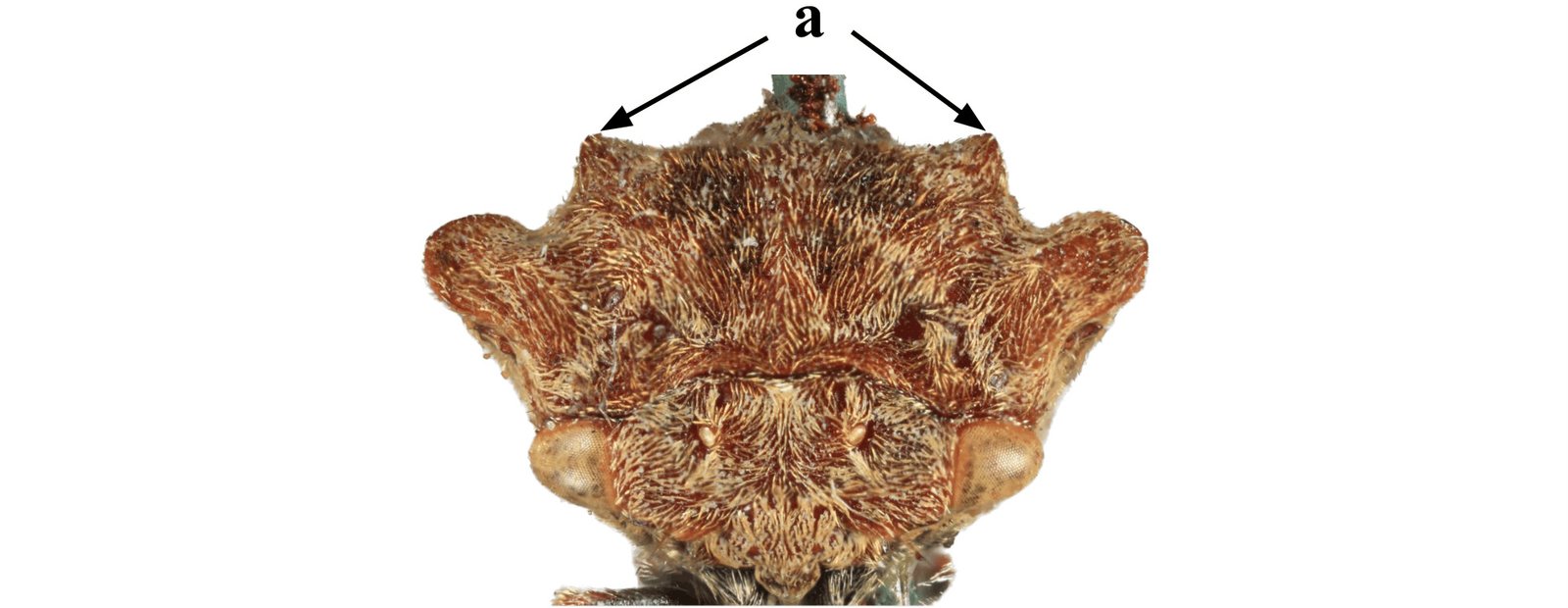		
–	No tubercles present between humeral angles (b)	***Sipylus* sp**.
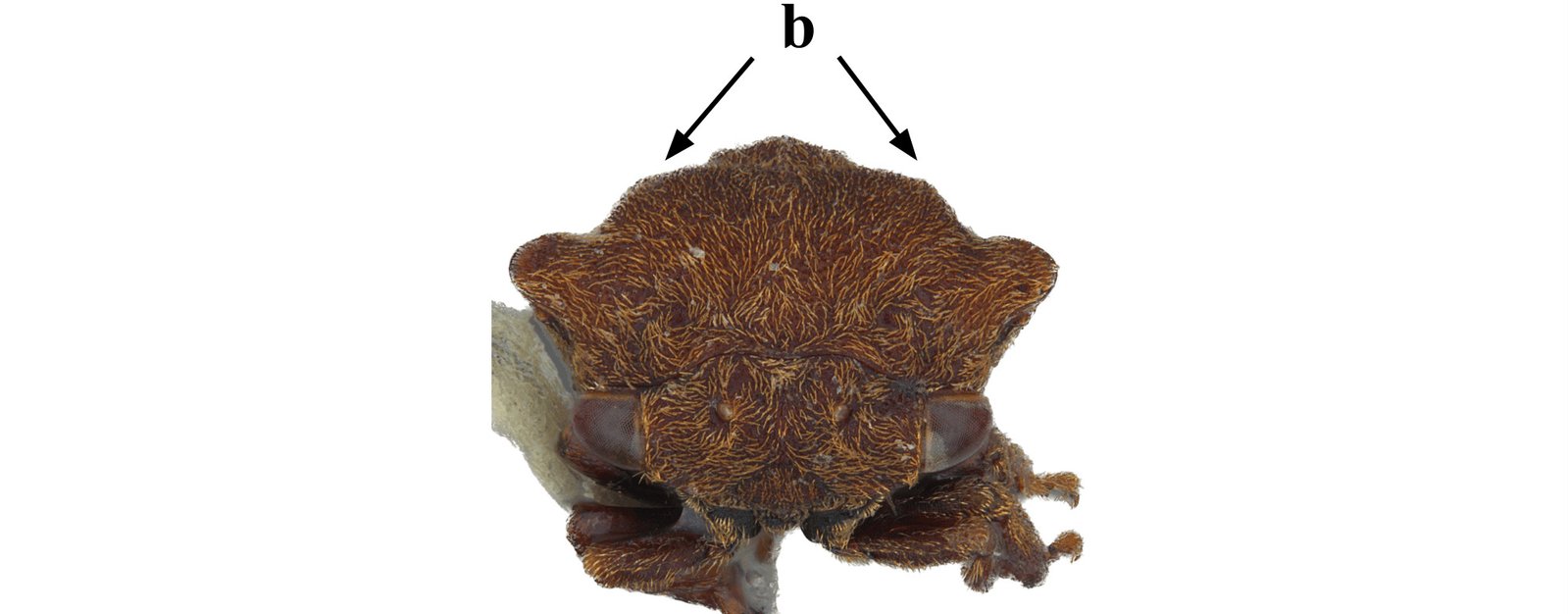		
23	Suprahumerals absent (a)	***Tricentrus acuticornis* Funkhouser, 1919** ♂
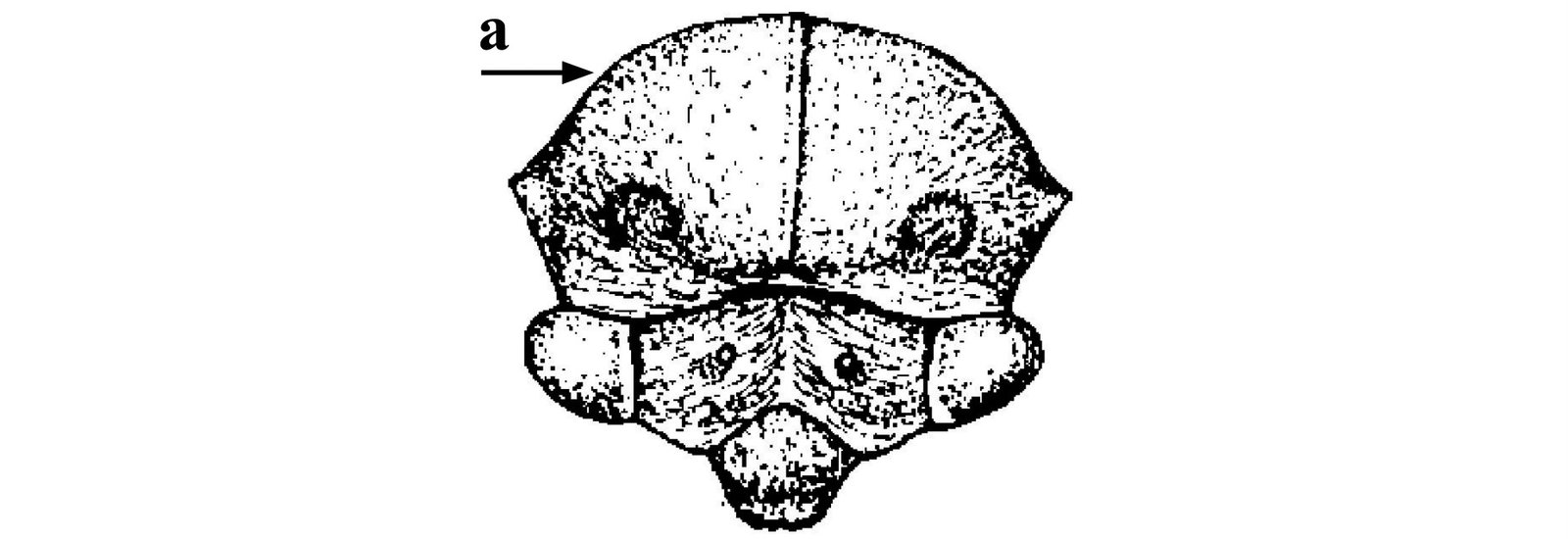		
–	Suprahumerals present (b)	**25**
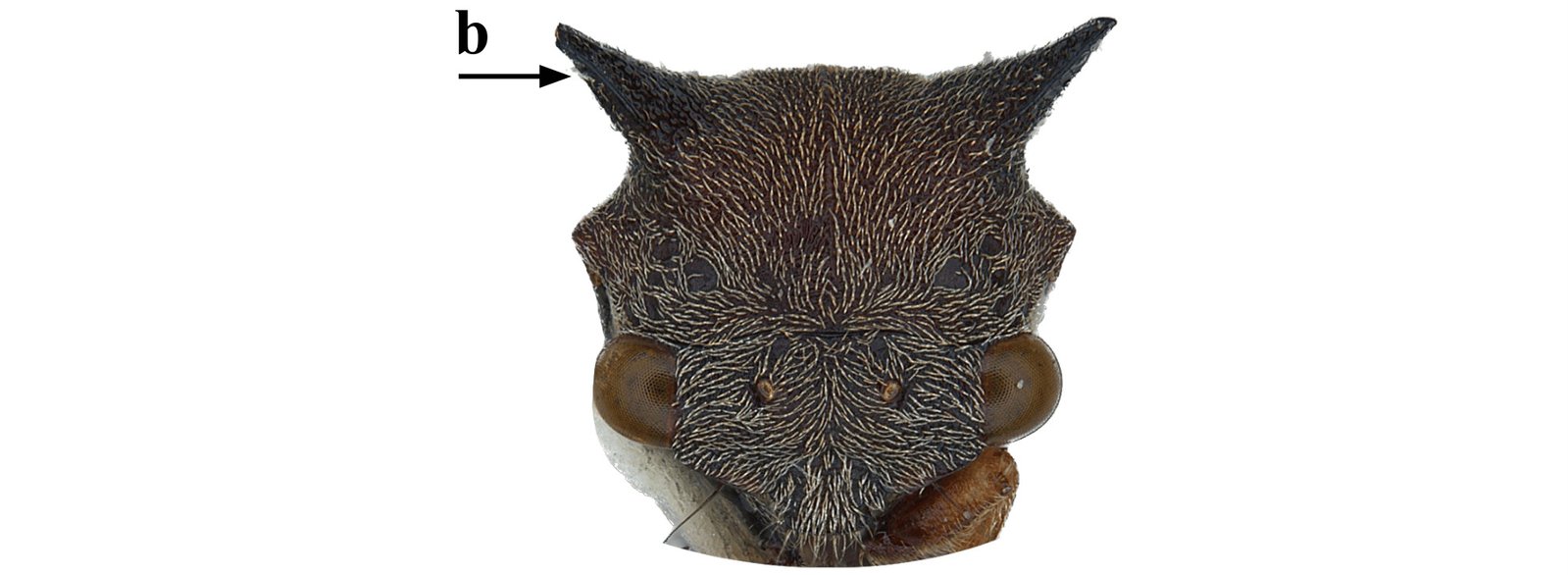		
24	Forewing with base sparsely punctate and somewhat translucent (a)	***Gargara projecta* Funkhouser, 1918**
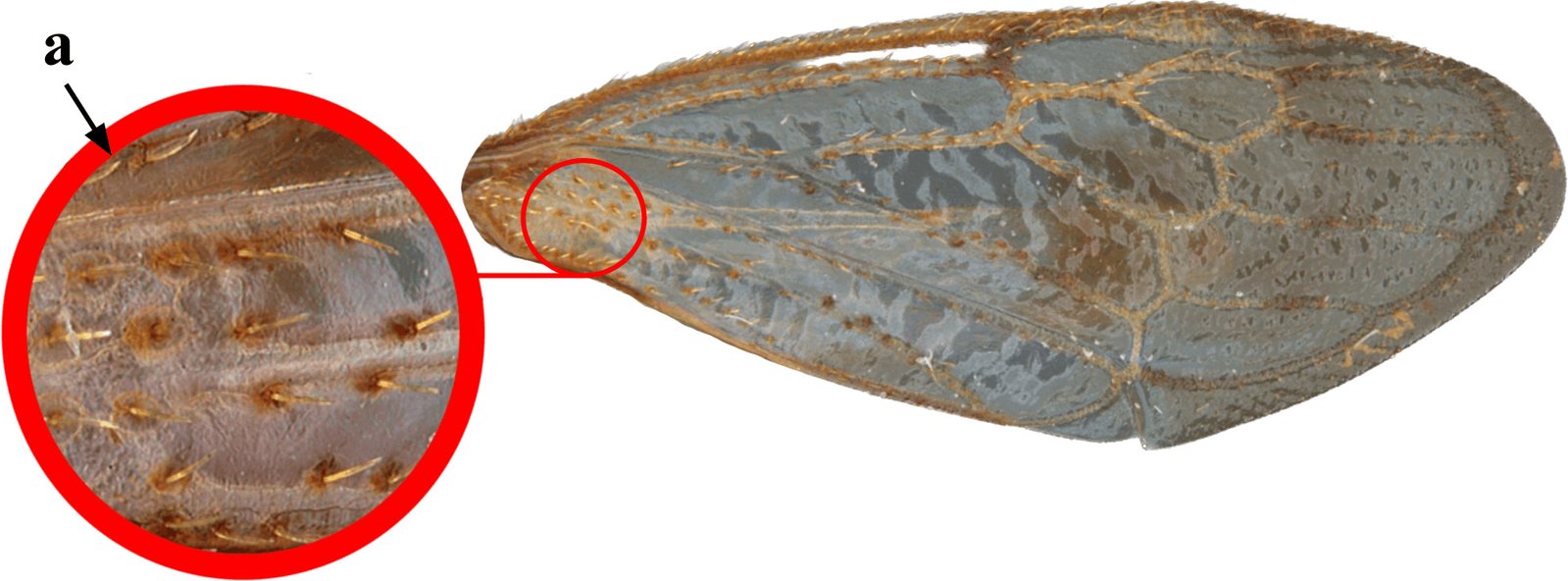		
–	Forewing with base distinctly punctate and opaque (b)	**26**
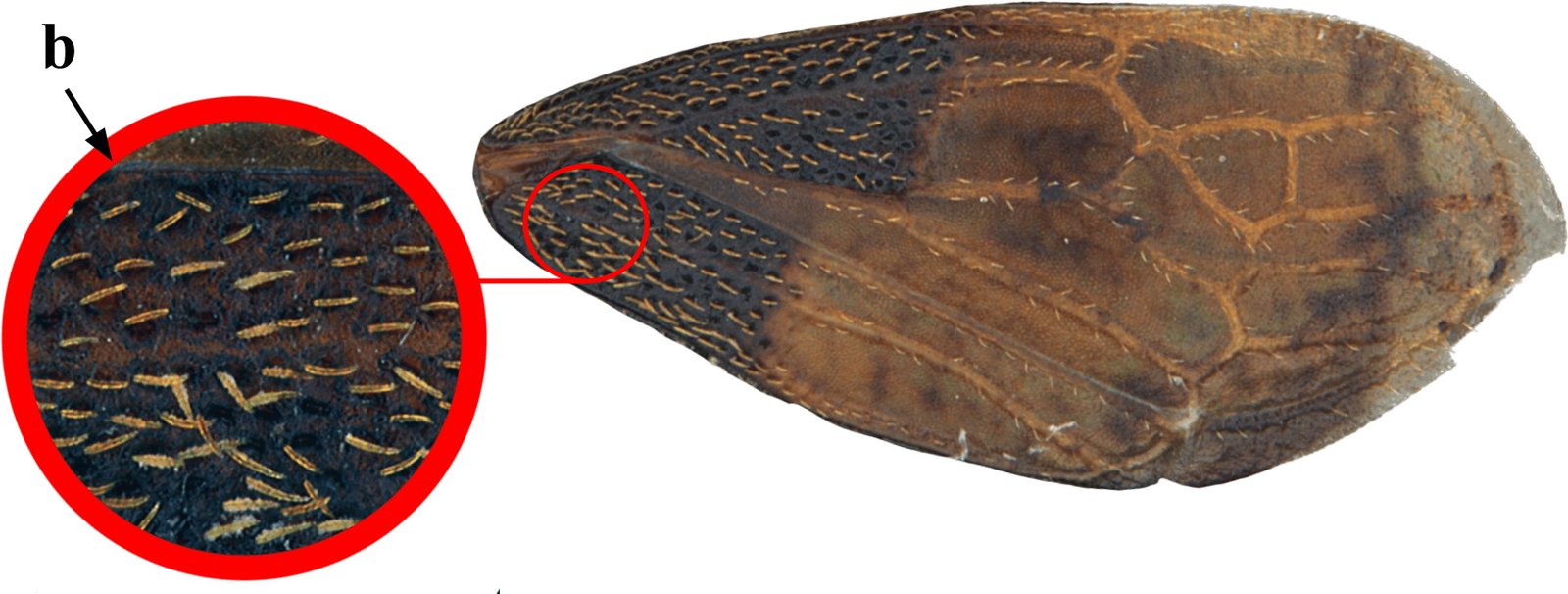		
25	Veins of forewing lined with distinct nodules (a); humeral angles inconspicuous (b)	**27**
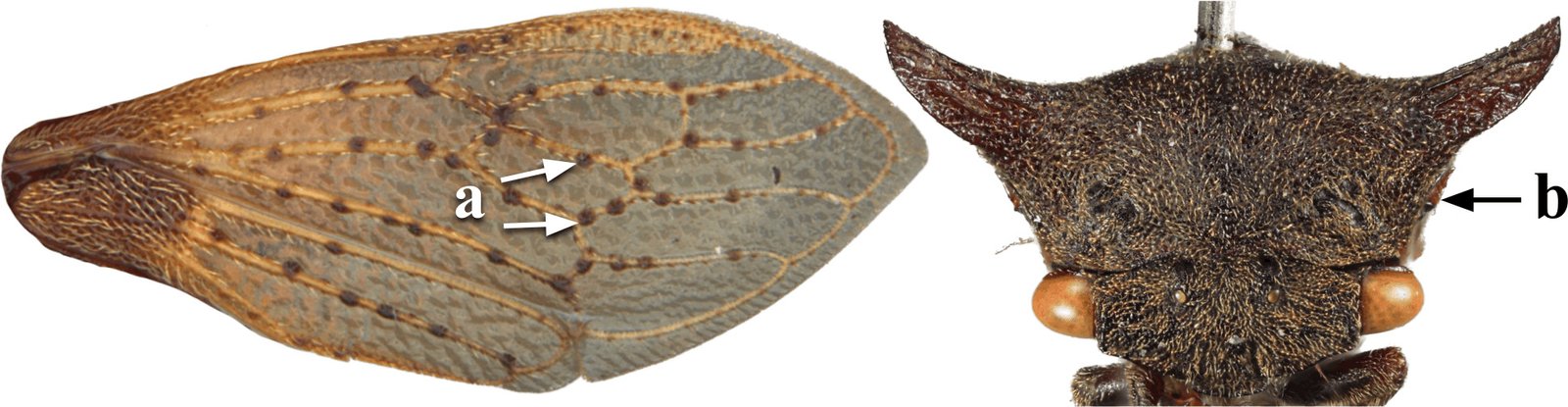		
–	Veins of forewing without distinct nodules (c); humeral angles conspicuous (d)	**28**
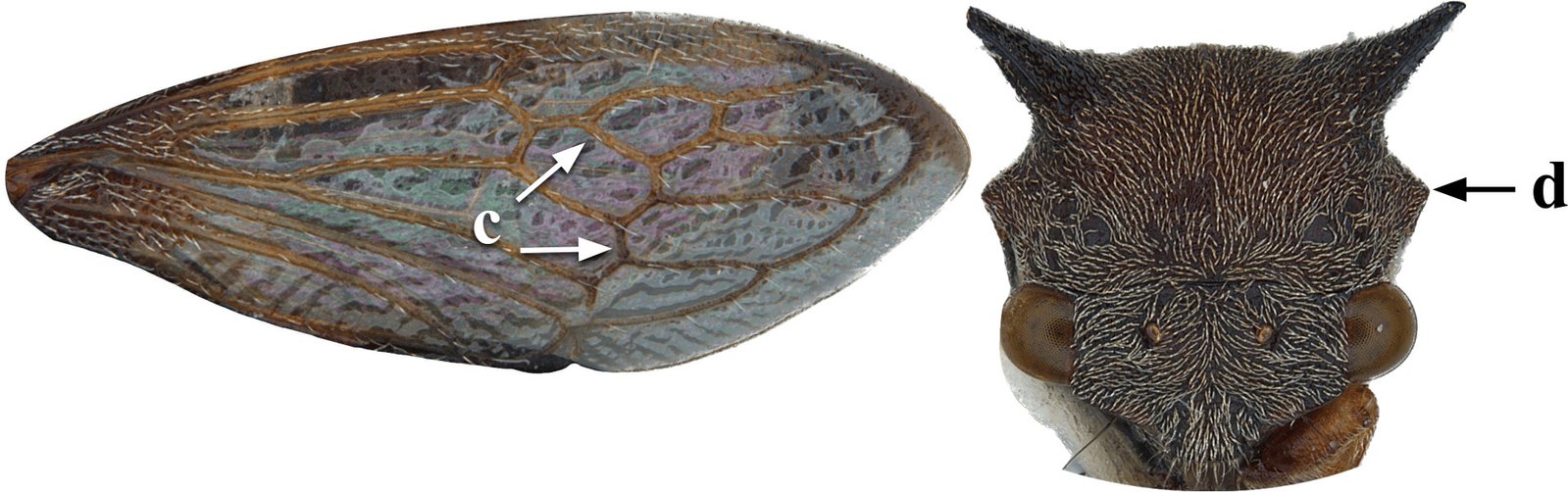		
26	Tip of posterior process not reaching 5ac of forewing (a)	**29**
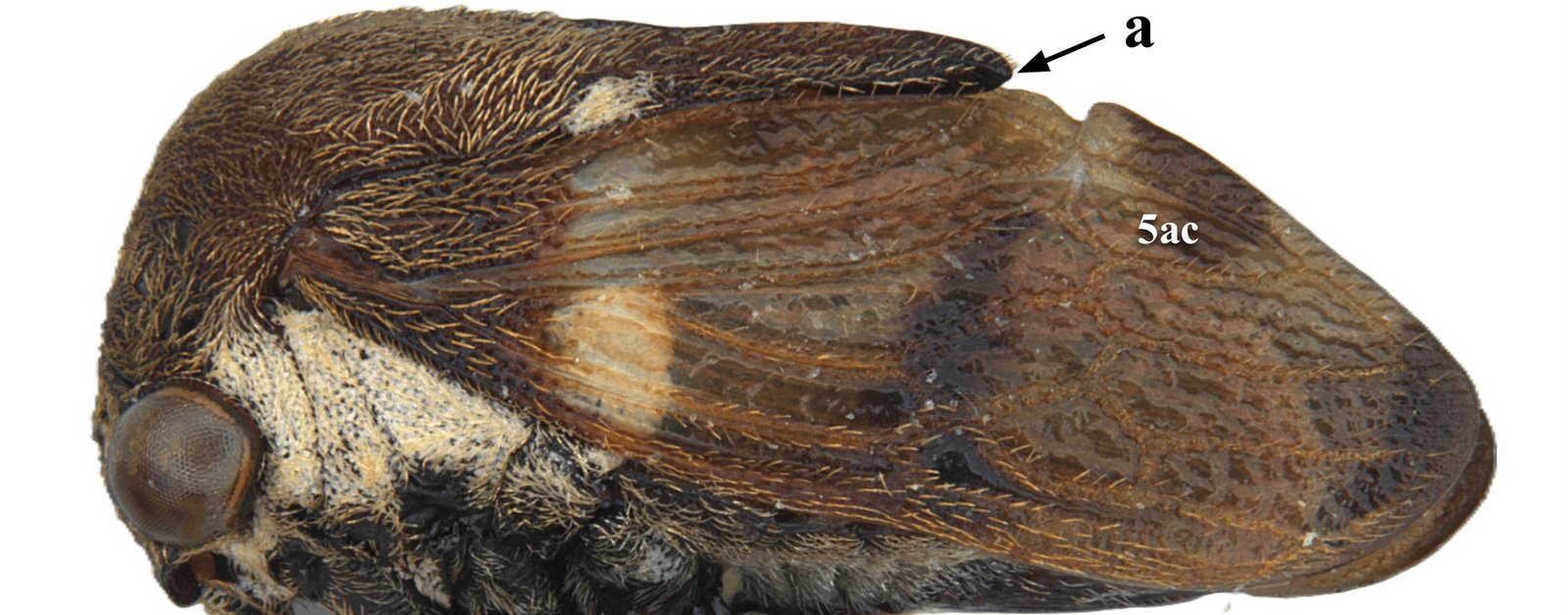		
–	Tip of posterior process just reaching or surpassing 5ac of forewing (b)	**30**
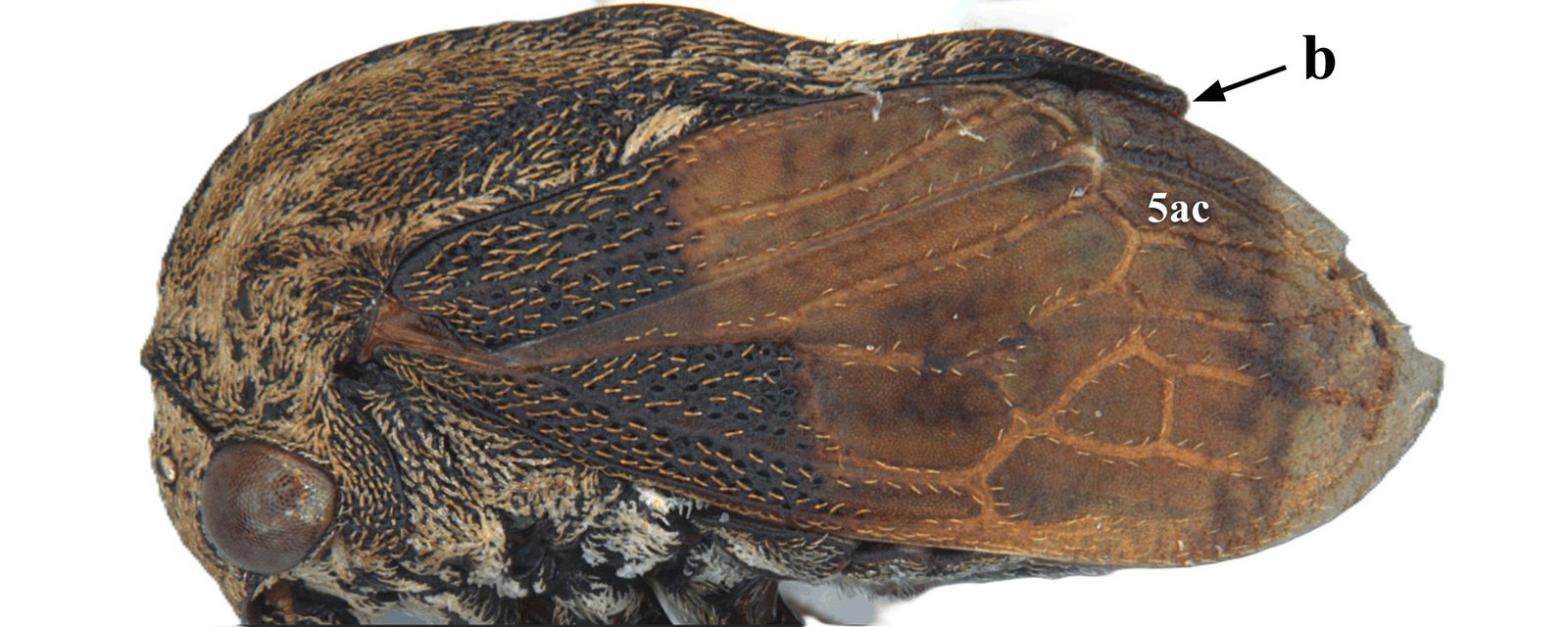		
27	Suprahumerals acuminate from front view (a)	***Tricentrus truncaticornis* Funkhouser, 1918**
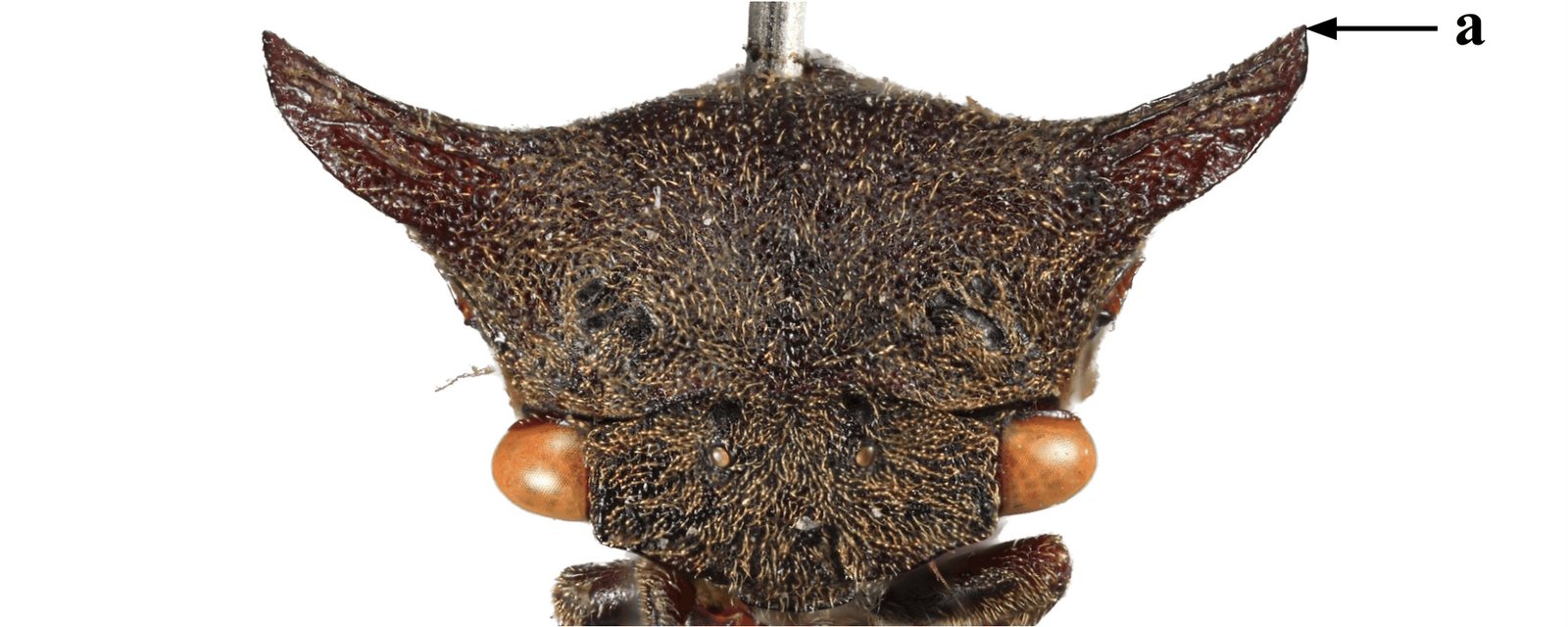		
–	Suprahumerals truncated from front view (b)	***Tricentrus intermedius* (Schmidt, 1911)**
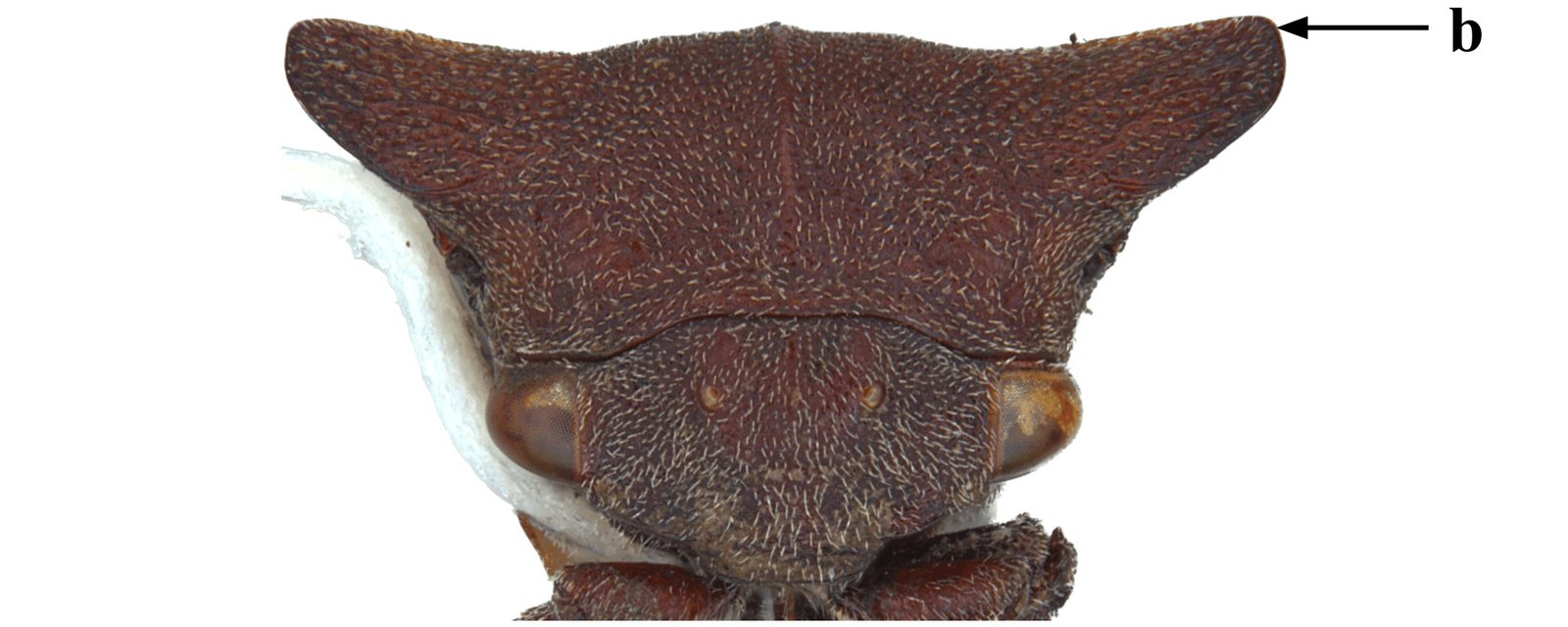		
28	Pronotum distinctly shiny (a); eyes distinctly red (b)	***Tricentrus fulgidus* Funkhouser, 1929**
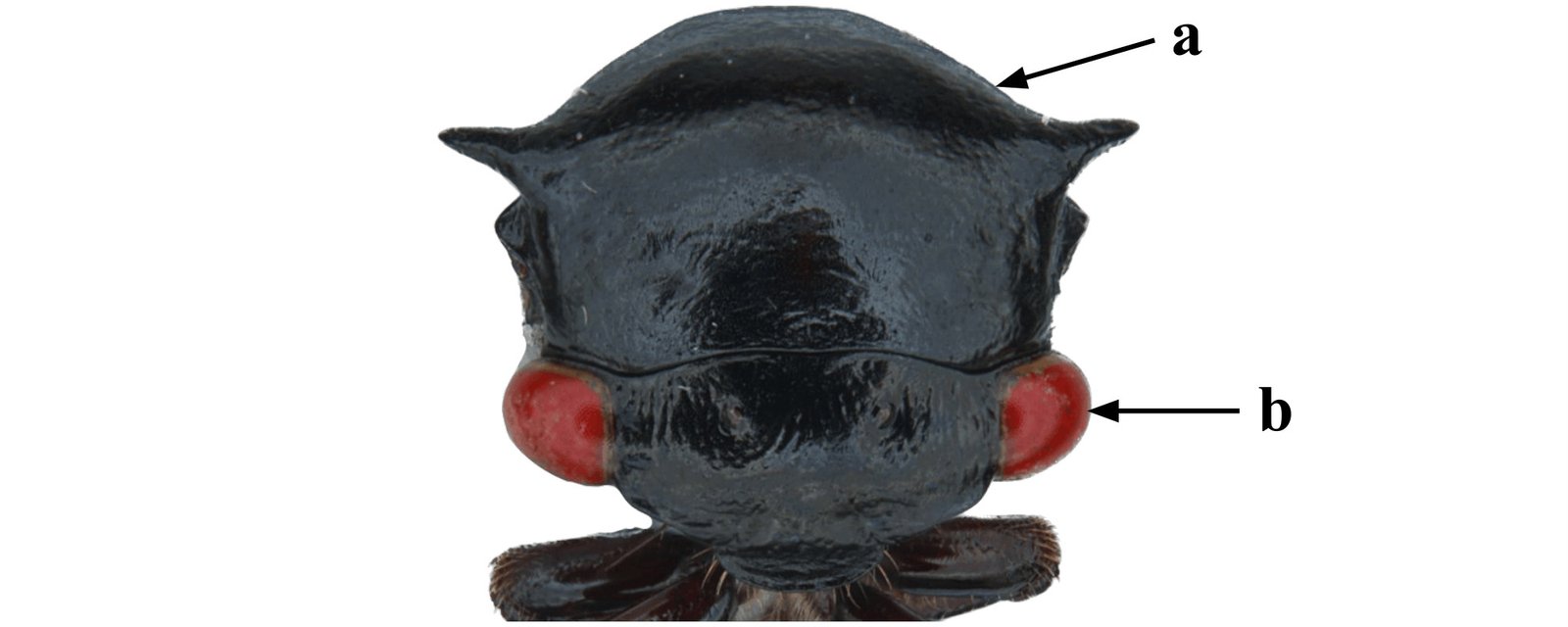		
–	Pronotum not shiny (c); eyes never red (d)	**31**
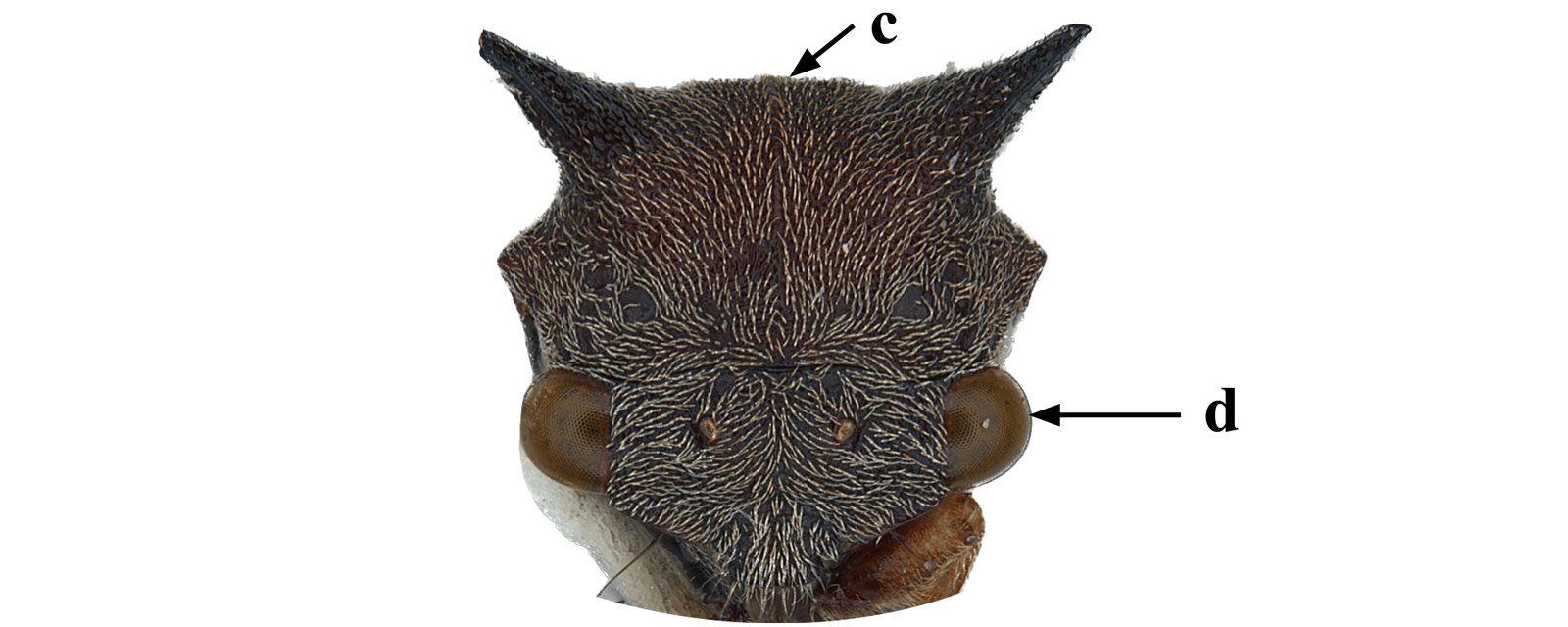		
29	Forewing with 3ac constricted medially (a)	***Gargara nervosa* Funkhouser, 1918**
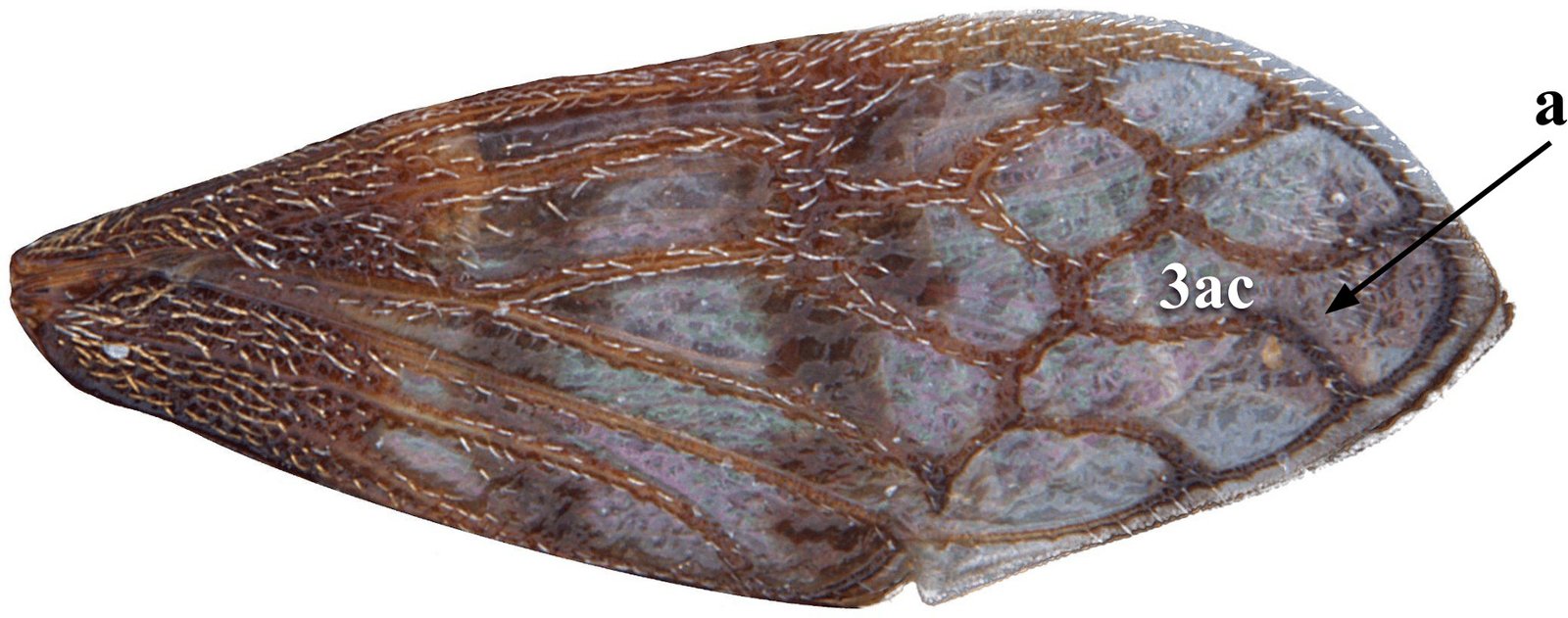		
–	Forewing with 3ac unconstricted (b)	**32**
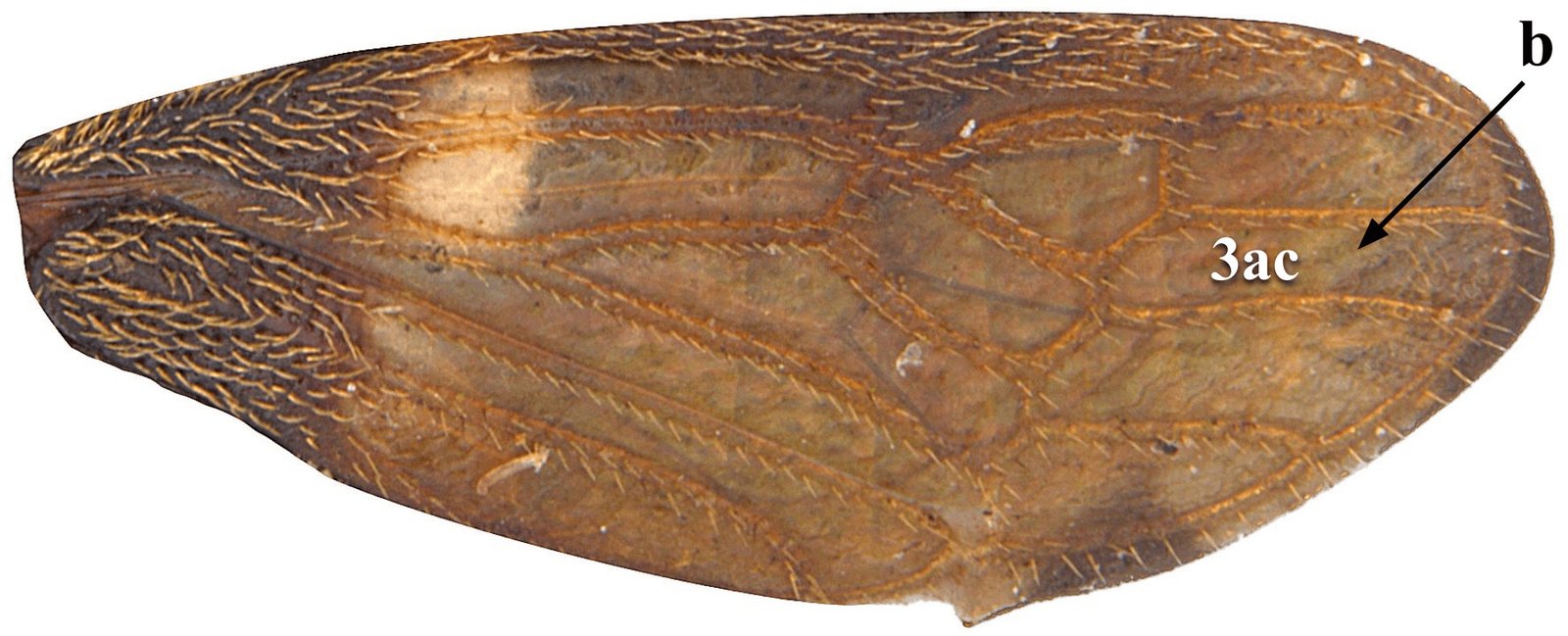		
30	Median carina produced with distinct bright band percurrent through the metopidium (a)	***Gargara nitidipennis* Funkhouser, 1914**
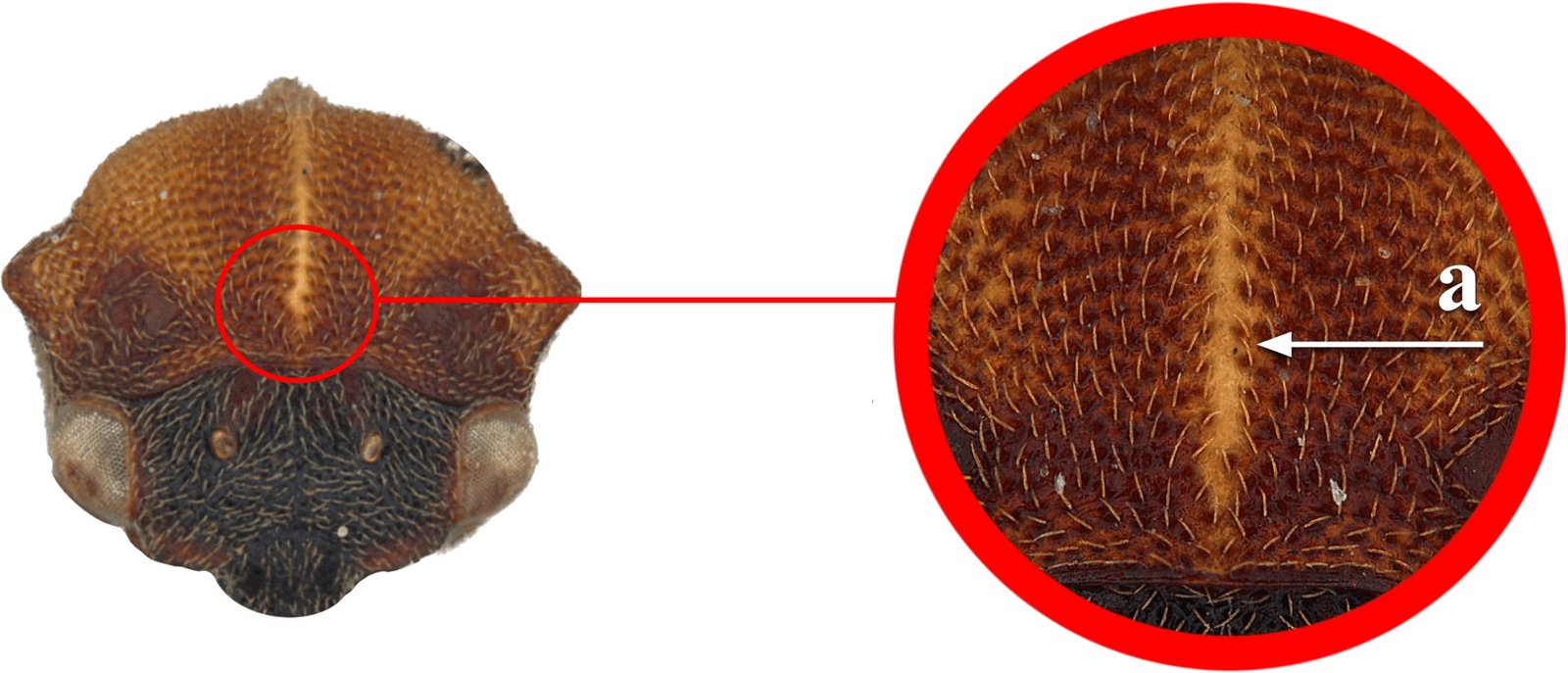		
–	Median carina percurrent through the metopidium but of dull colouration (b) or, median carina indistinct (c)	**33**
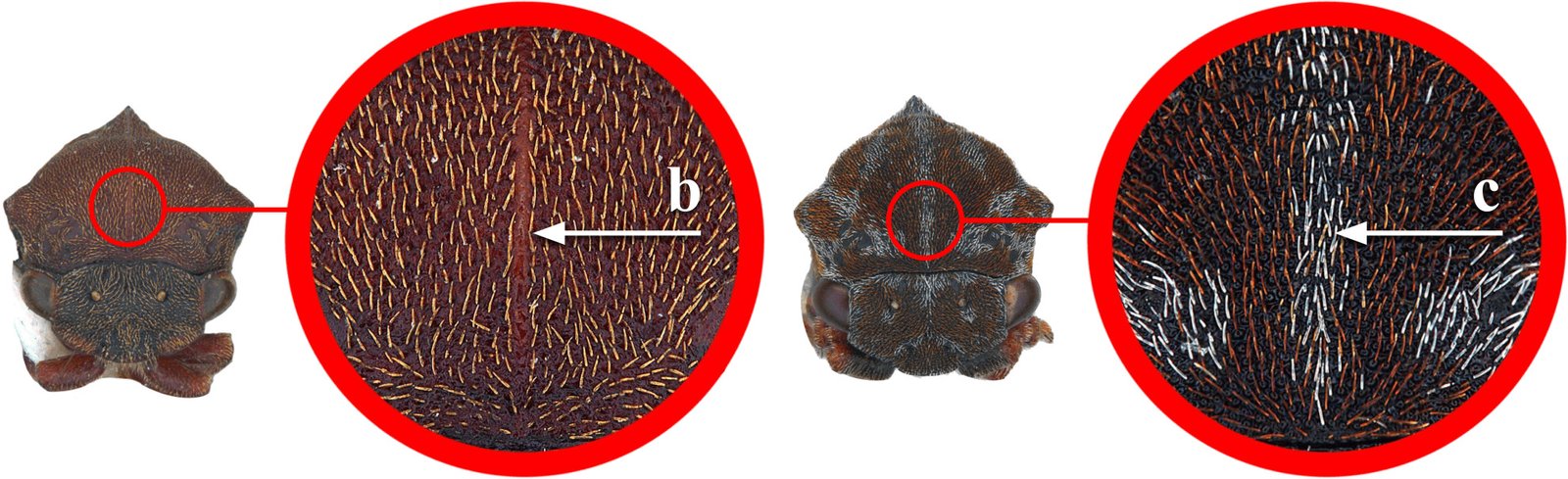		
31	Metapleuron with white tomentose patch visible through forewing (a)	**34**
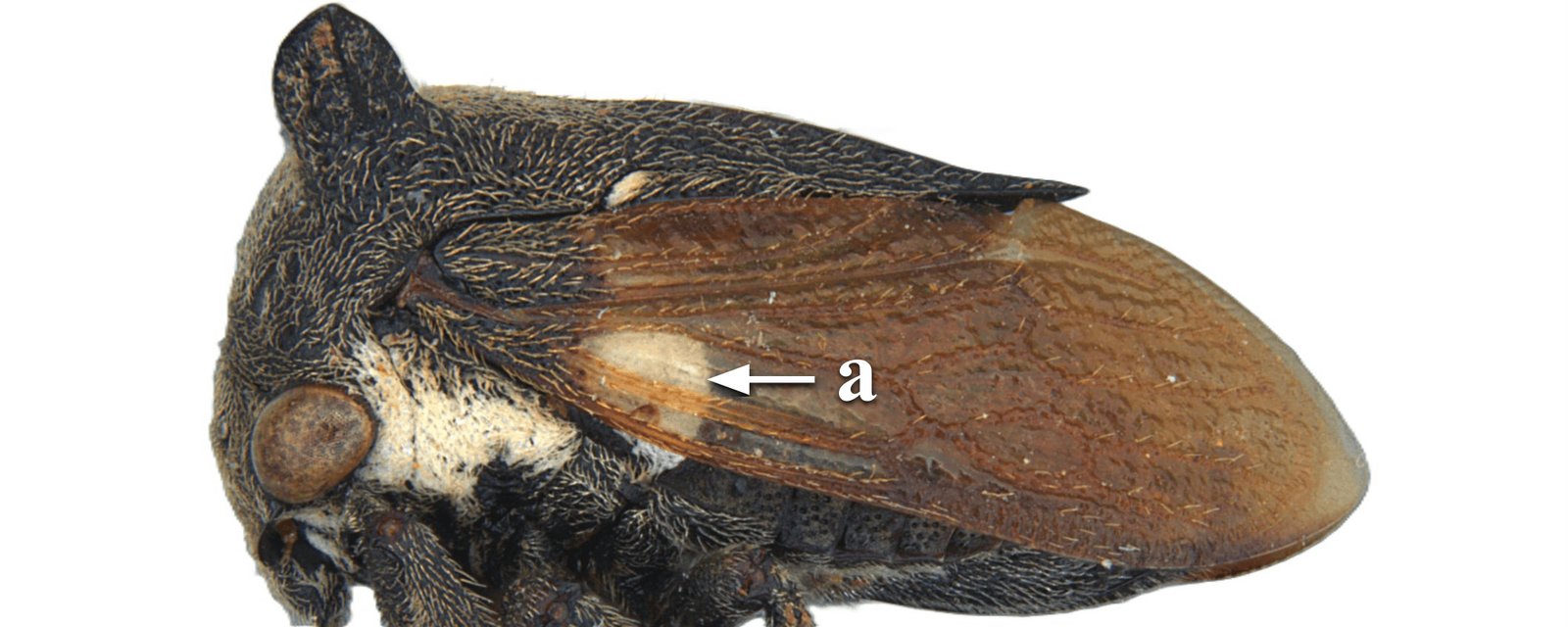		
–	No white tomentosity on the metapleuron discernible through forewing (b)	**35**
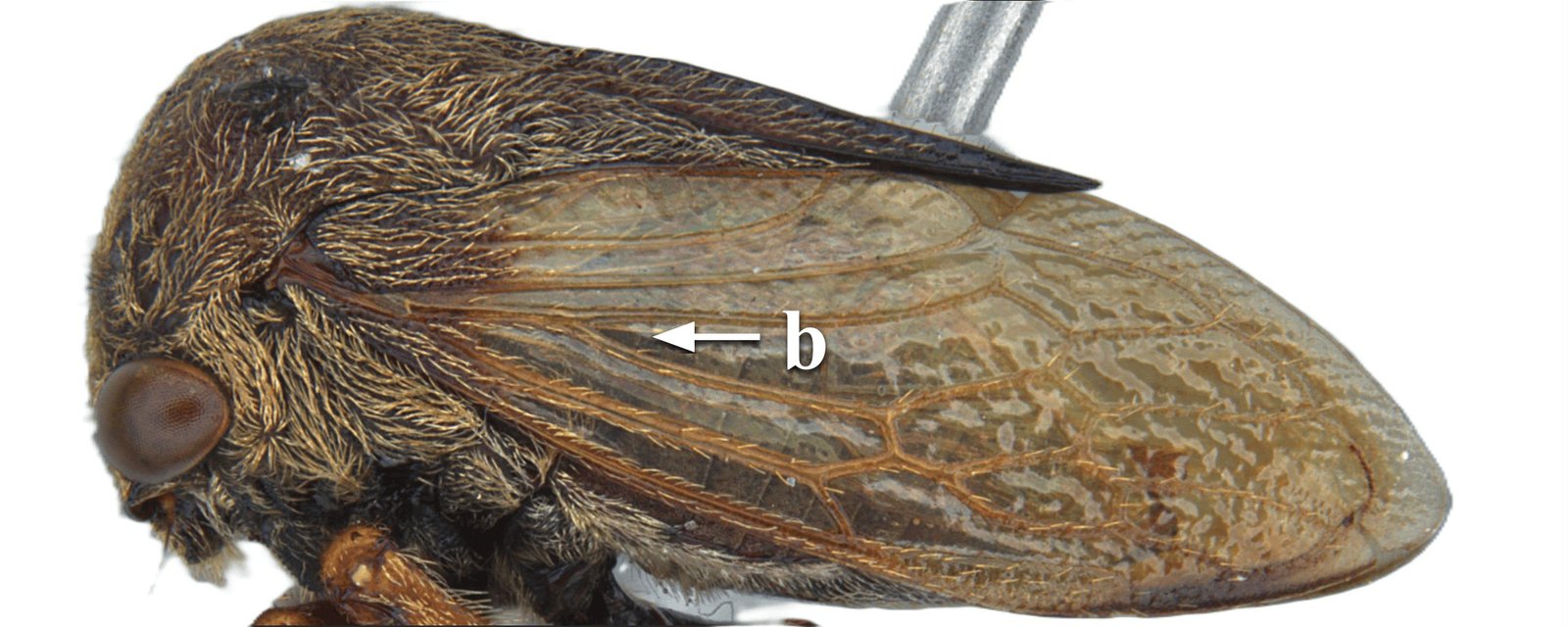		
32	Base of scutellum with distinct white tomentosity visible from lateral view (a); pleuron clothed in white tomentosity (b), metapleuron with white tomentose patch visible through forewing (c)	***Gargara sordida* Funkhouser, 1918** ♀
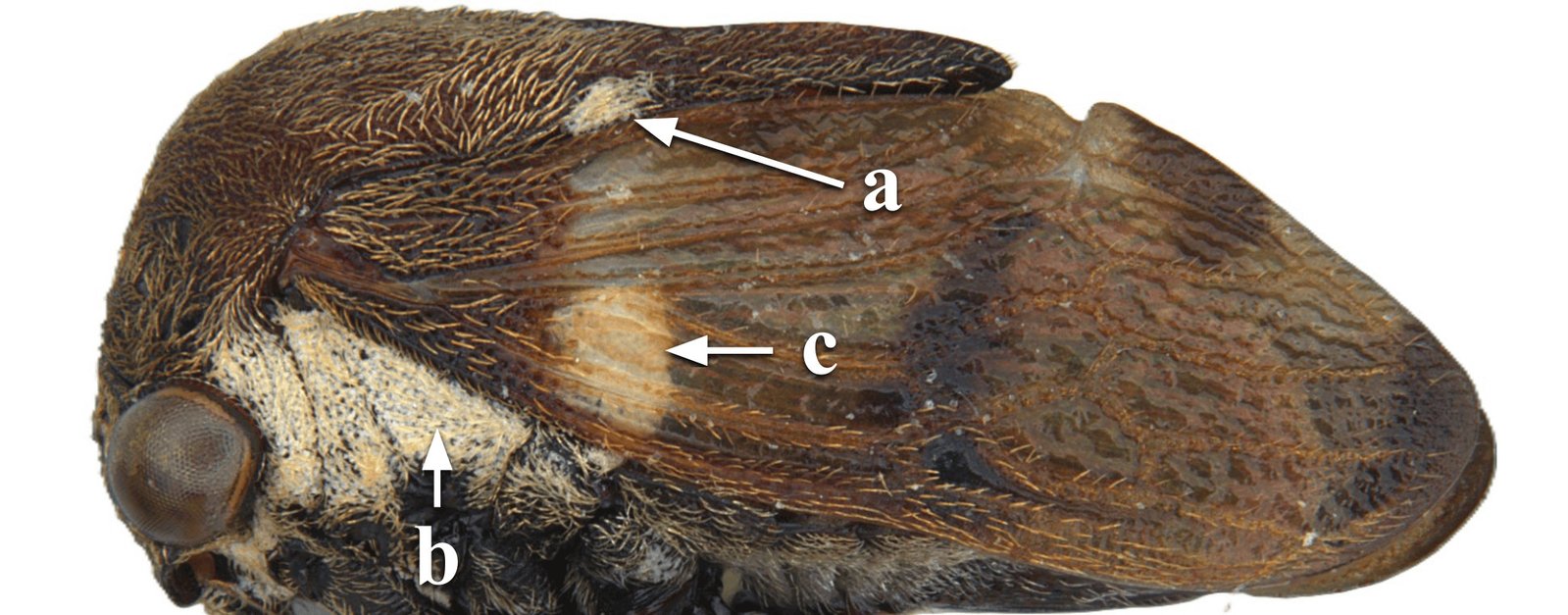		
–	Base of scutellum black and sparsely haired (d); pleuron clothed in silver hairs (e), no white tomentosity on the metapleuron discernible through forewing (f)	***Gargara* sp. 1**
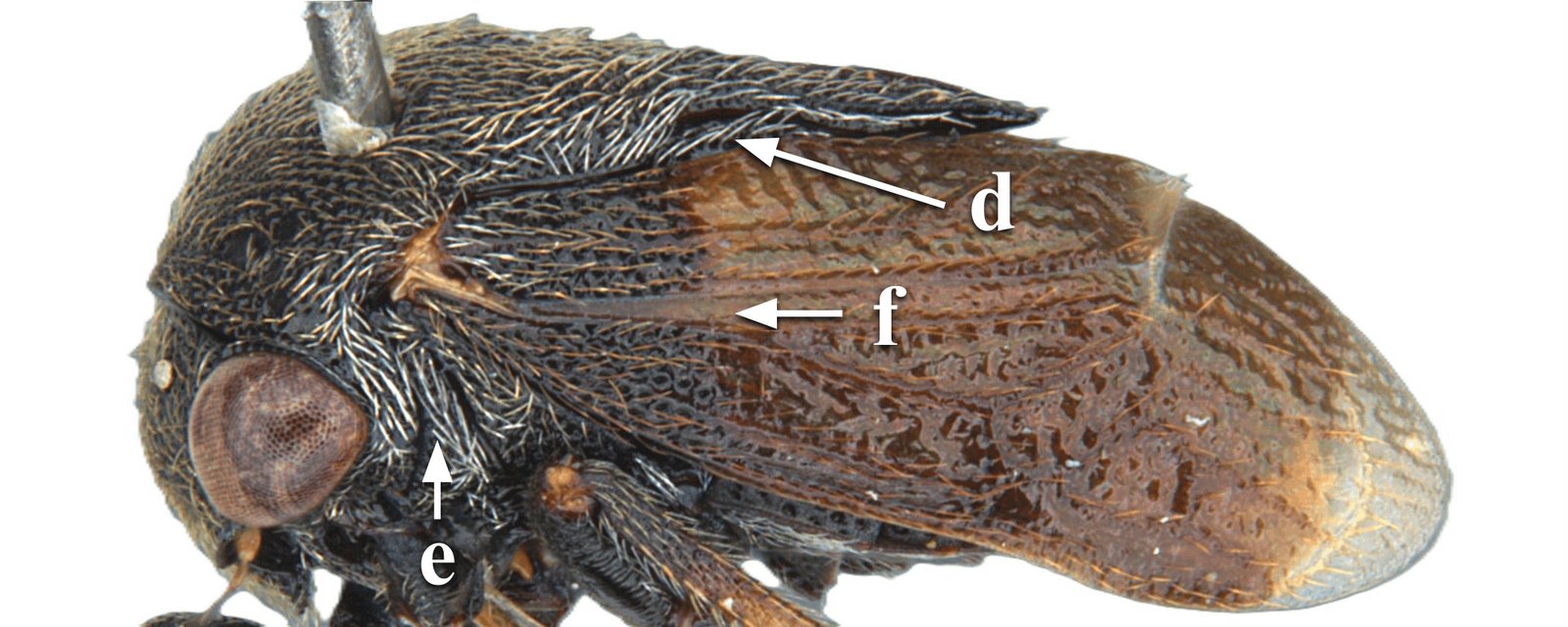		
33	Veins of forewing red bearing distinct nodules (a)	***Gargara rubrogranulata* Bierman, 1910**
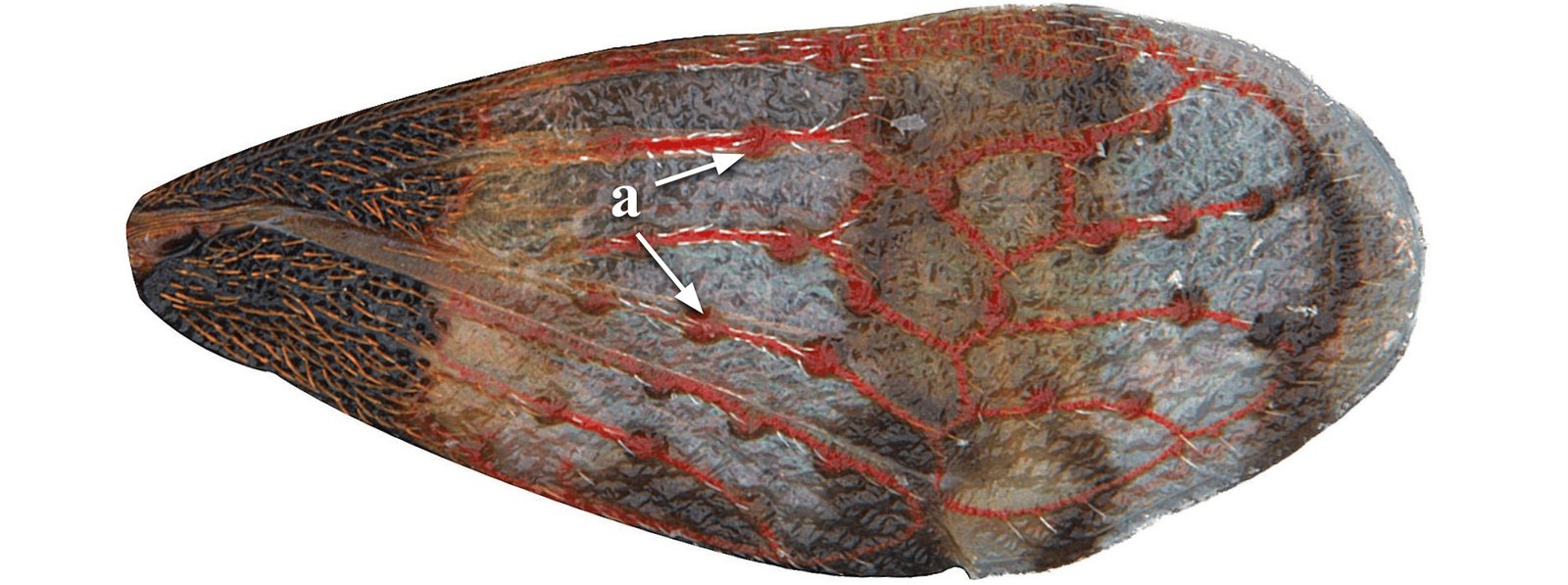		
–	Veins of forewing brown without distinct nodules (b)	**36**
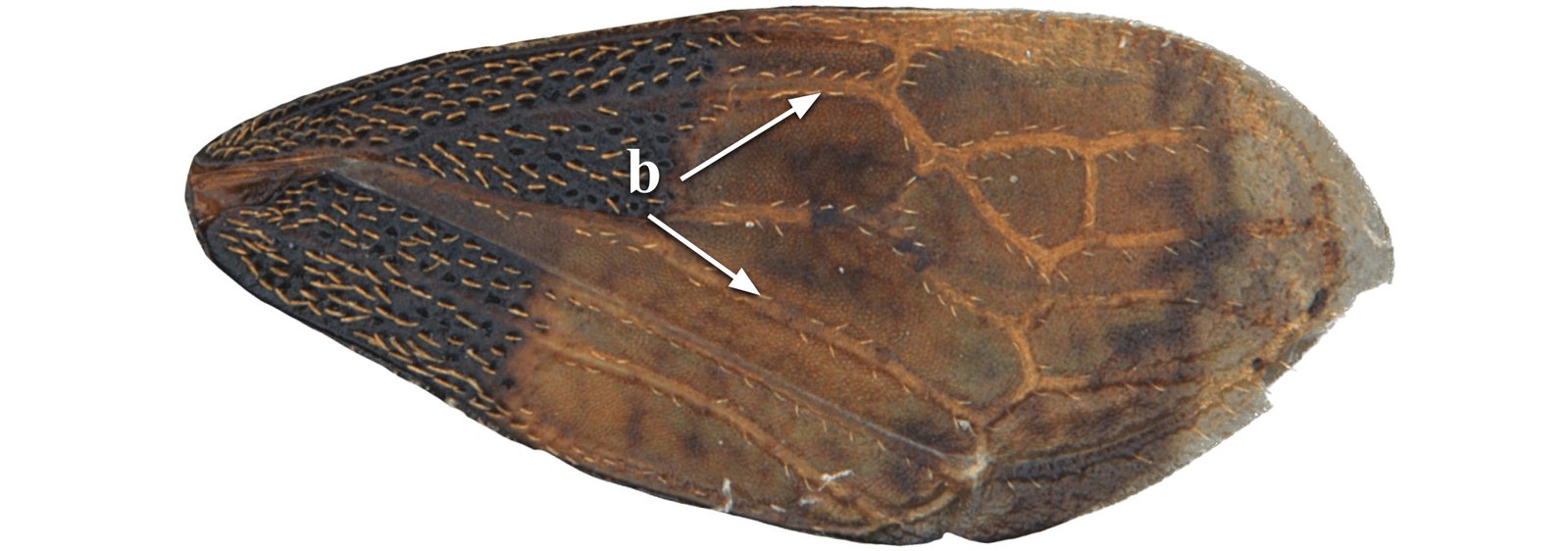		
34	Suprahumerals strongly directed anteriorly from lateral view (a); tibiae ochraceous (b)	***Tricentrus acuticornis* Funkhouser, 1919** ♀
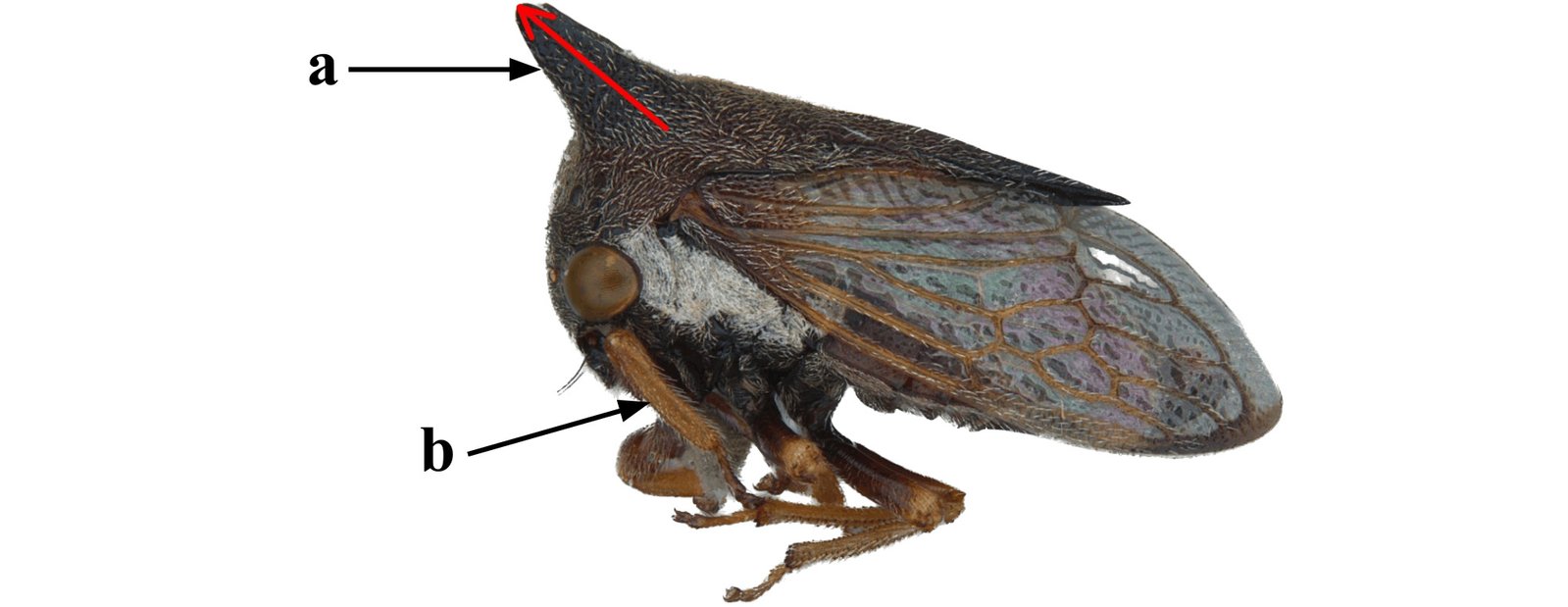		
–	Suprahumerals directed only slightly anteriorly from lateral view (c); tibiae black (d)	***Tricentrus cf. albomaculatus* Distant, 1908** ♀
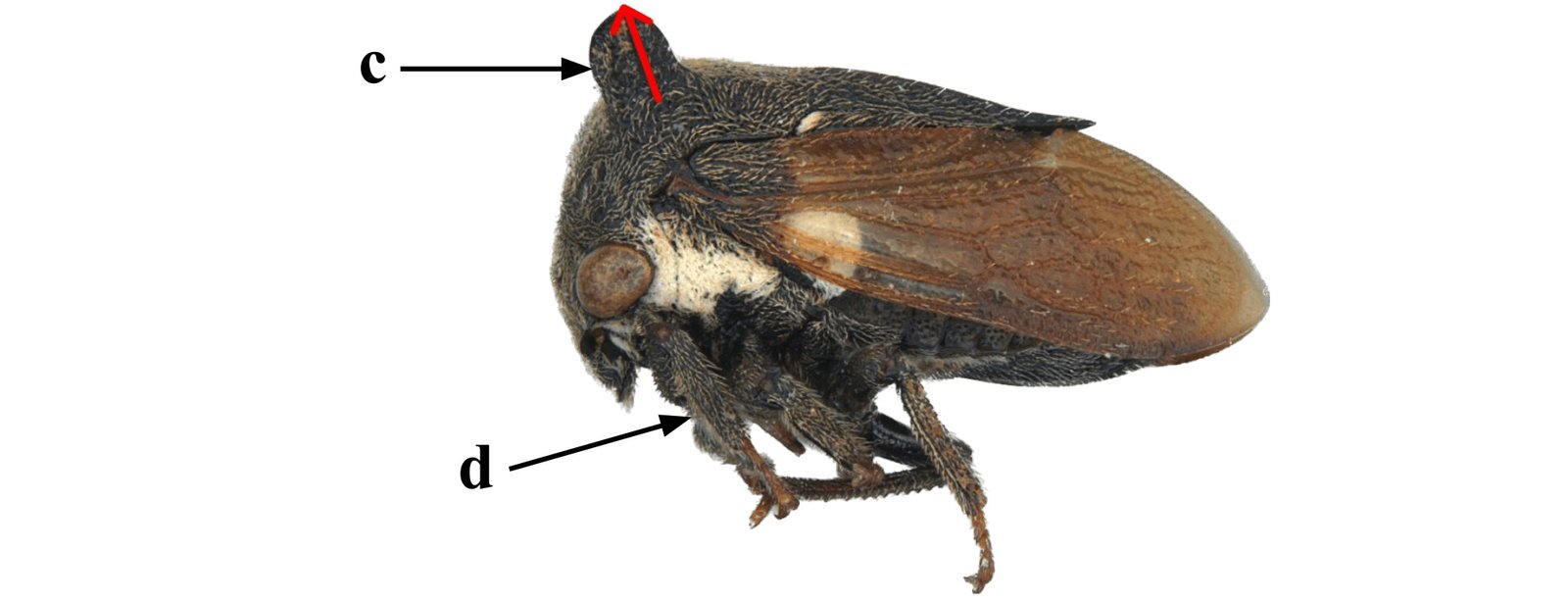		
35	Length of suprahumeral distinctly longer than the length of its base (a)	***Tricentrus spinicornis* Funkhouser, 1918** ♀
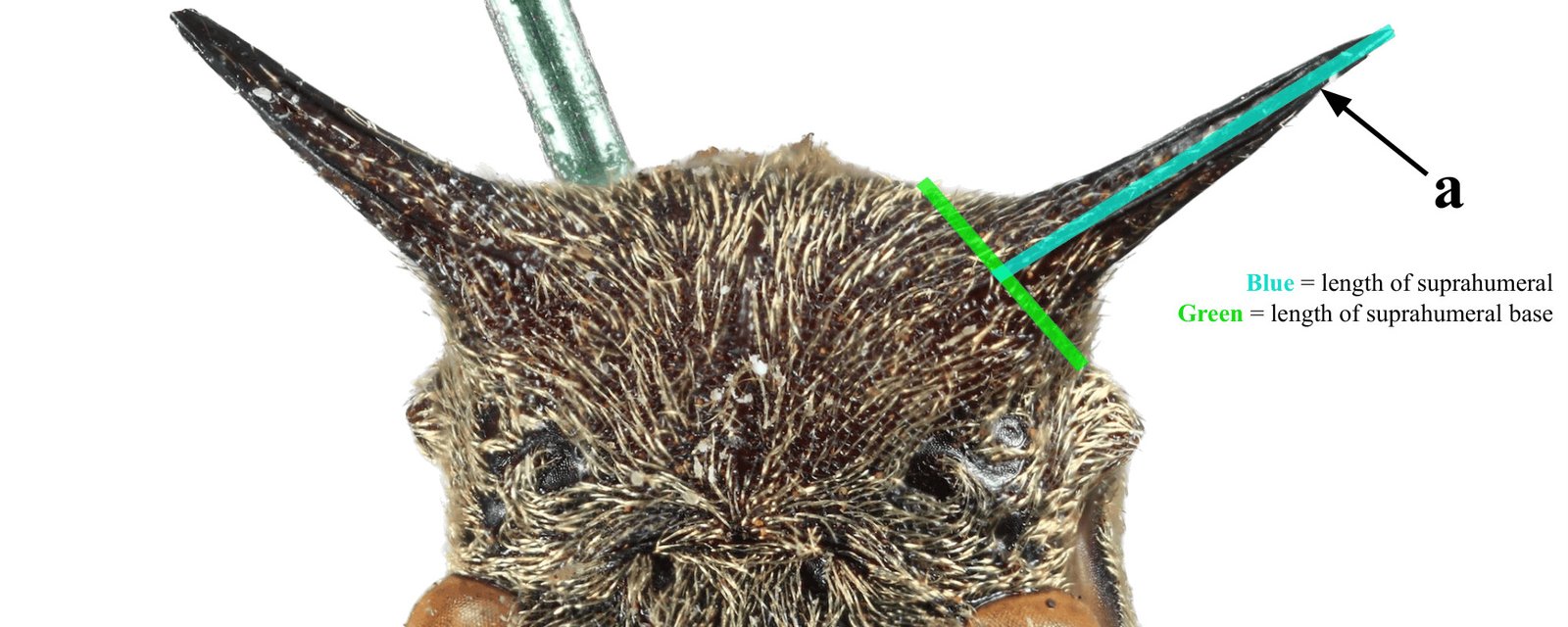		
–	Length of suprahumeral subequal to the length of its base (b)	**37**
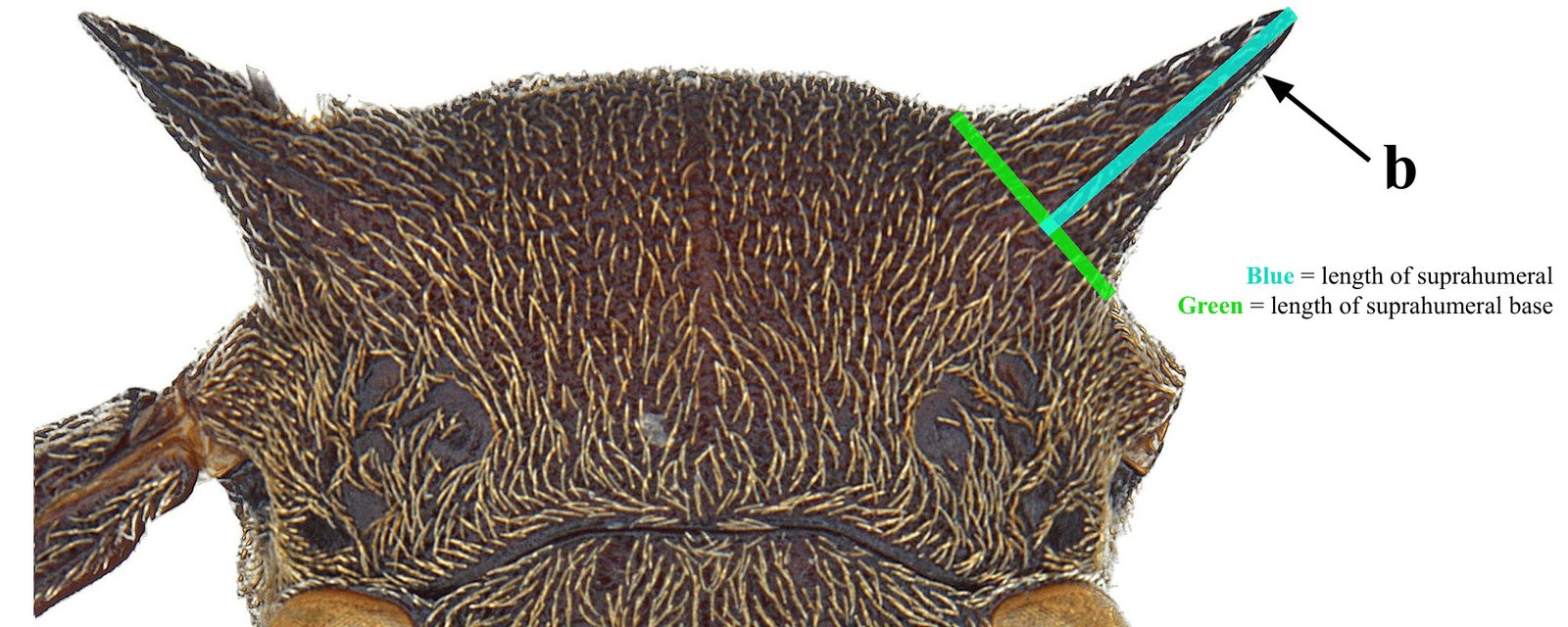		
36	Distinct yellowish band adjacent to the base of the forewing (a); pronotum plain from dorsal view (b)	***Gargara* sp. 2** ♀
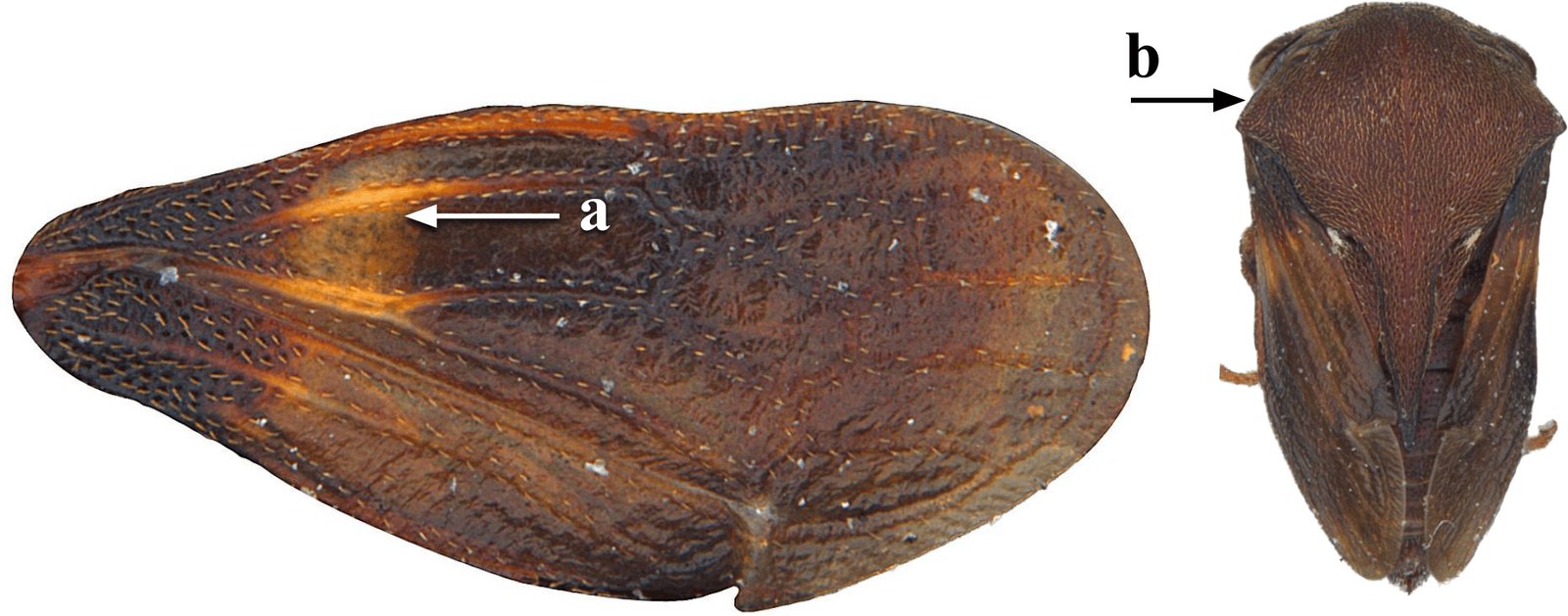		
–	Base of the forewing without a lightened band adjacent (c); pronotum adorned with symmetrical markings from dorsal view (d)	**38**
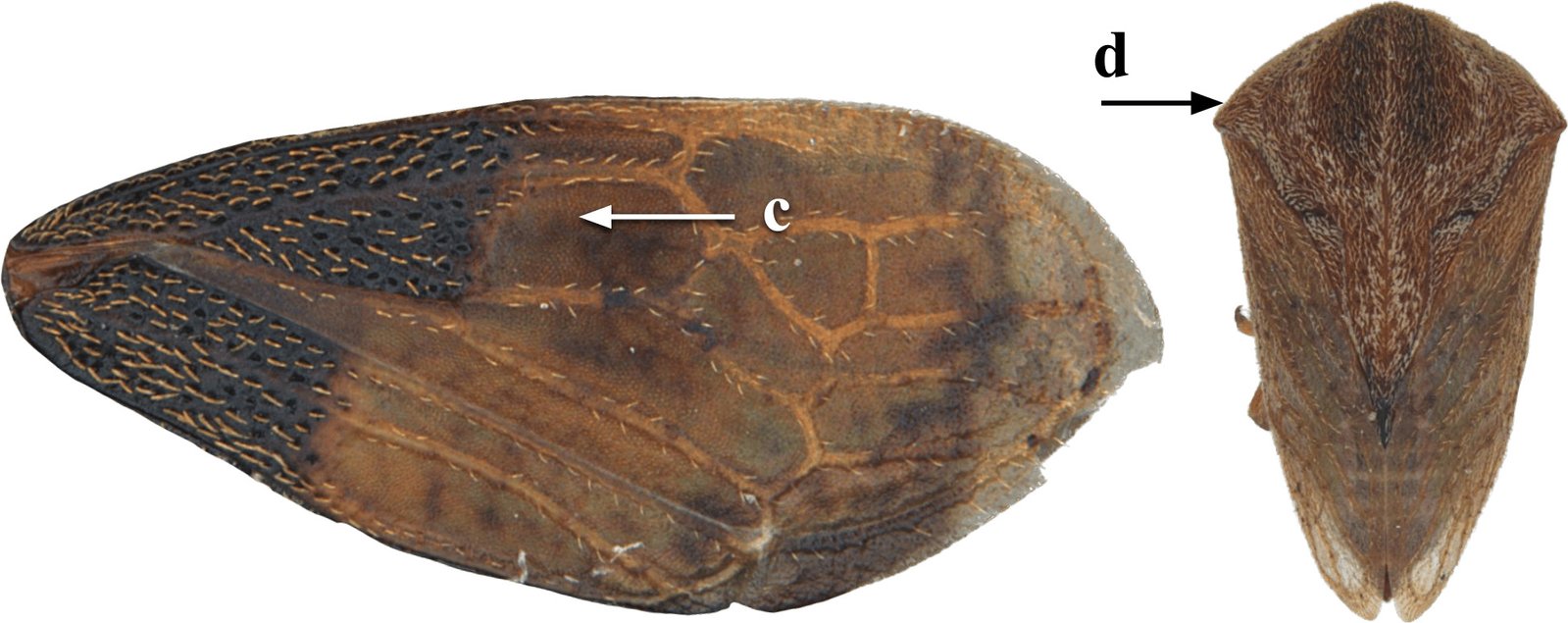		
37	Forewing without any darkened spots at the costal-apical region (a)	***Tricentrus banguensis* Funkhouser, 1914**
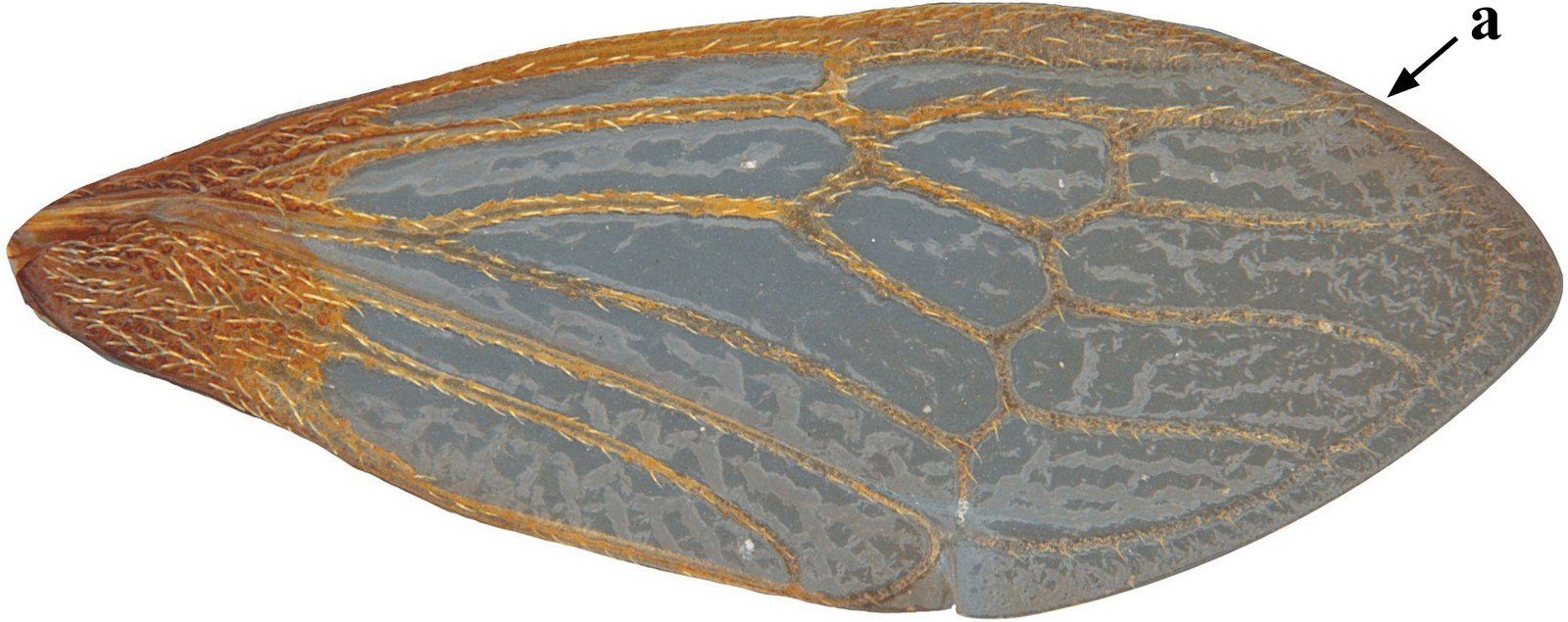		
–	Forewing with distinct darkened spot at costal-apical region (b)	**39**
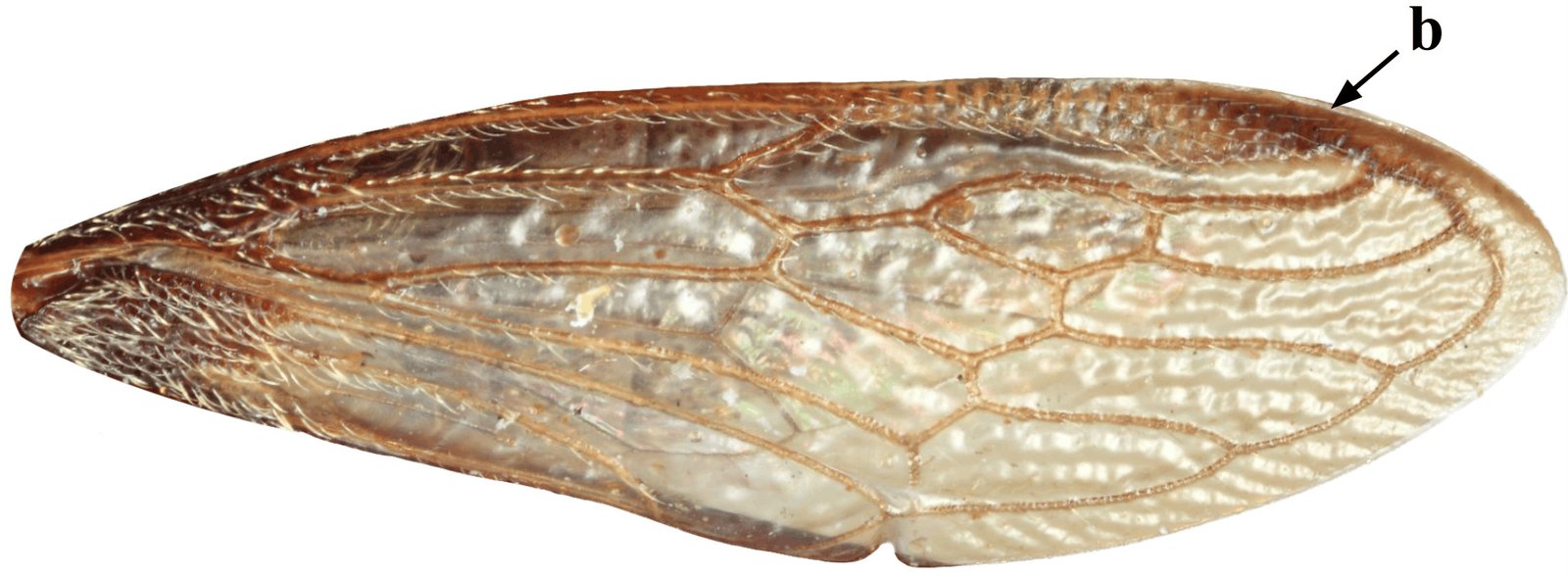		
38	Punctate base of forewing black, distinct from the remainder of the forewing (a)	***Gargara luconica* (Fairmaire, 1846)**
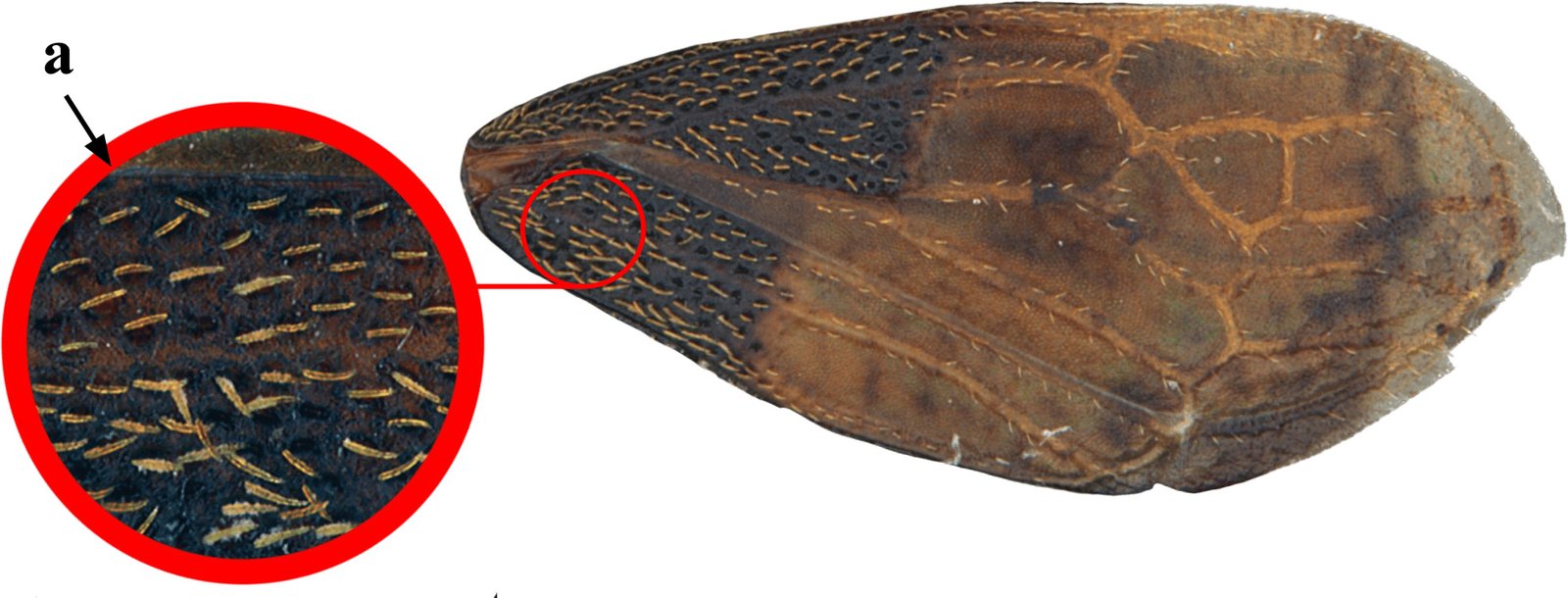		
–	Punctate base of forewing brown, somewhat indiscernible from the remainder of the forewing (b)	***Gargara triangulata* Funkhouser, 1918** ♀
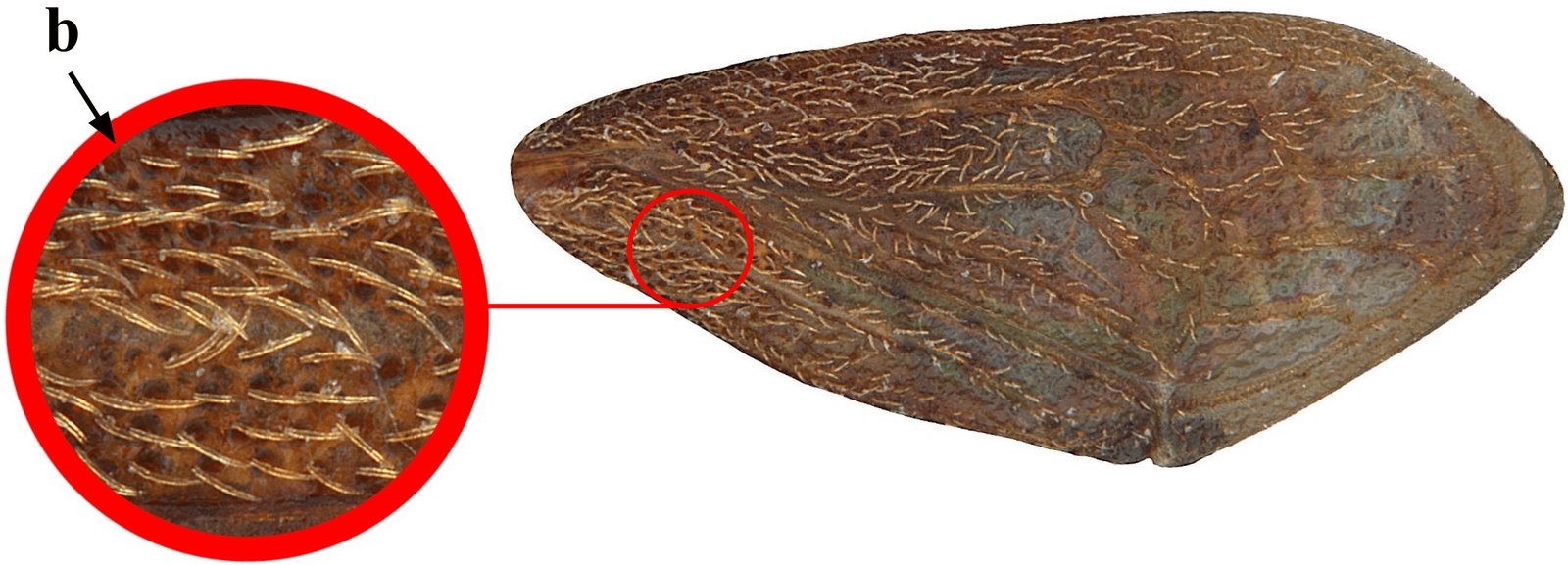		
39	Suprahumerals do not exceed dorsal margin of pronotum from the lateral view (a)	***Tricentrus spinis* Funkhouser, 1935**
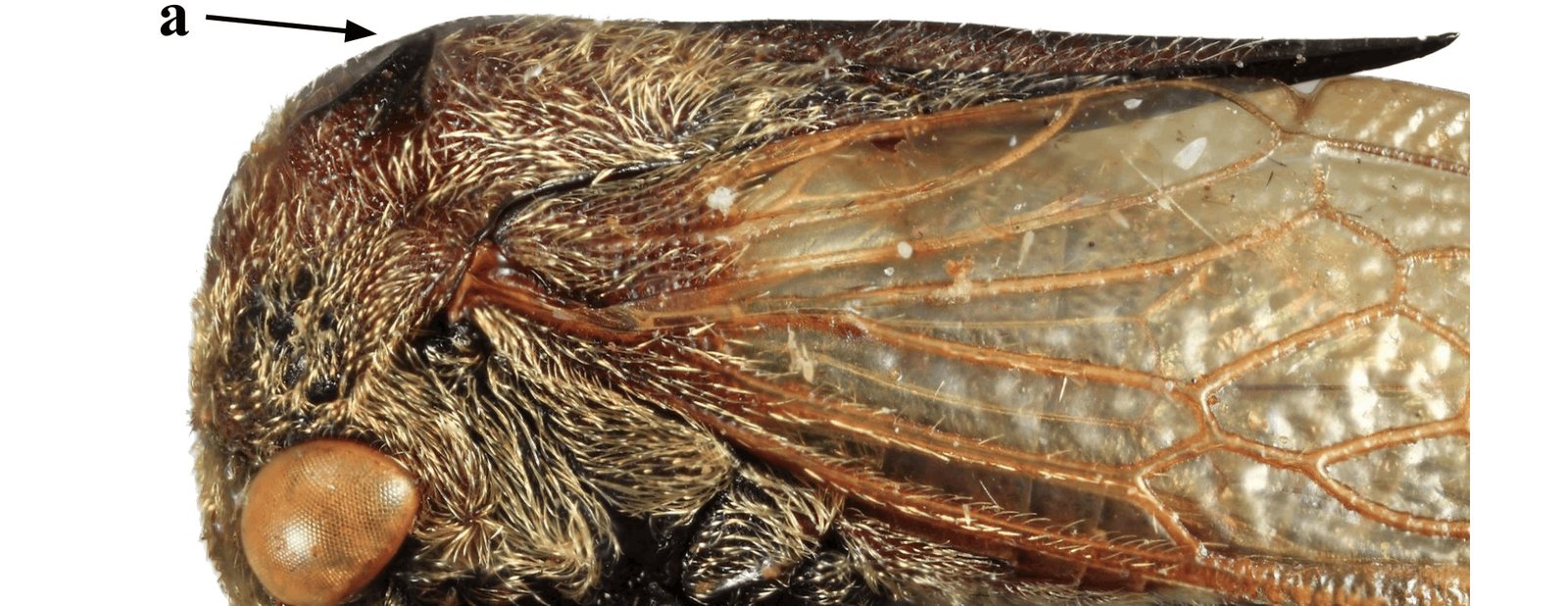		
–	Suprahumerals well exceed dorsal margin of pronotum from the lateral view (b)	**40**
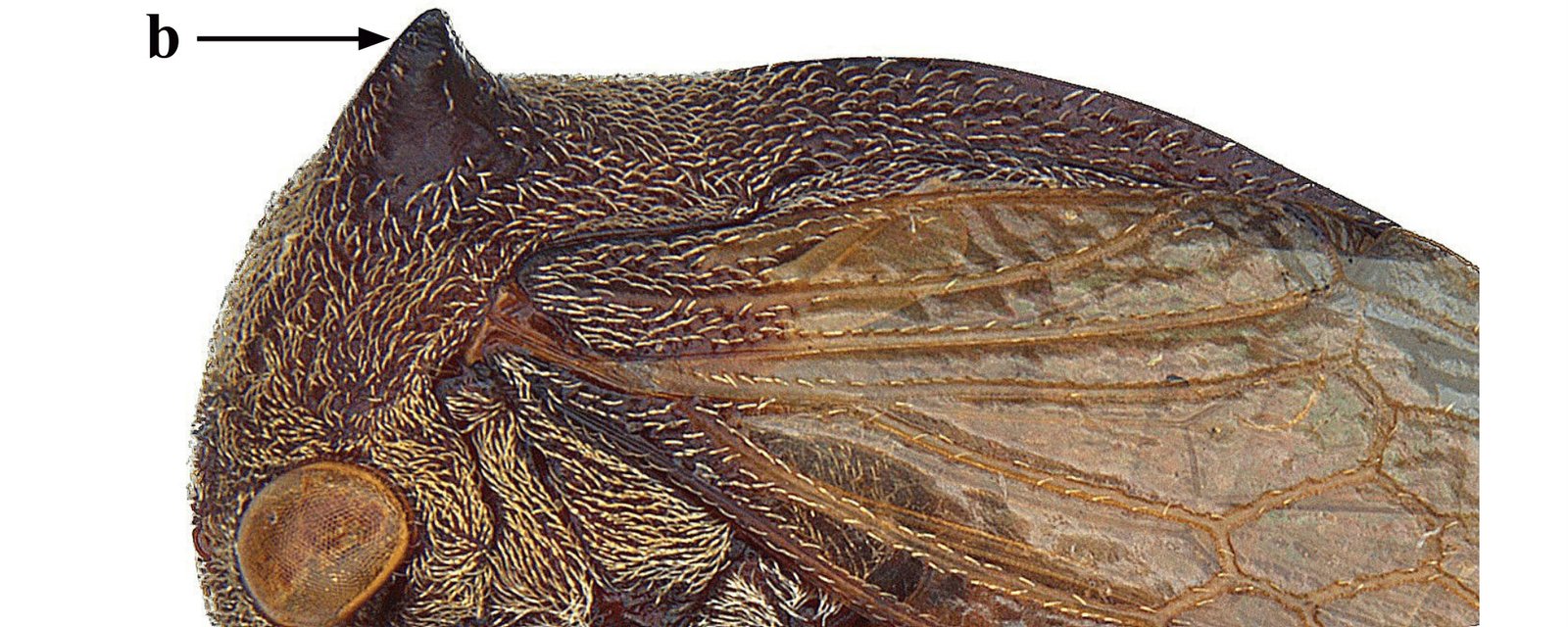		
40	Posterior process thin, straight (a)	***Tricentrus brunneus* Funkhouser, 1918** ♀
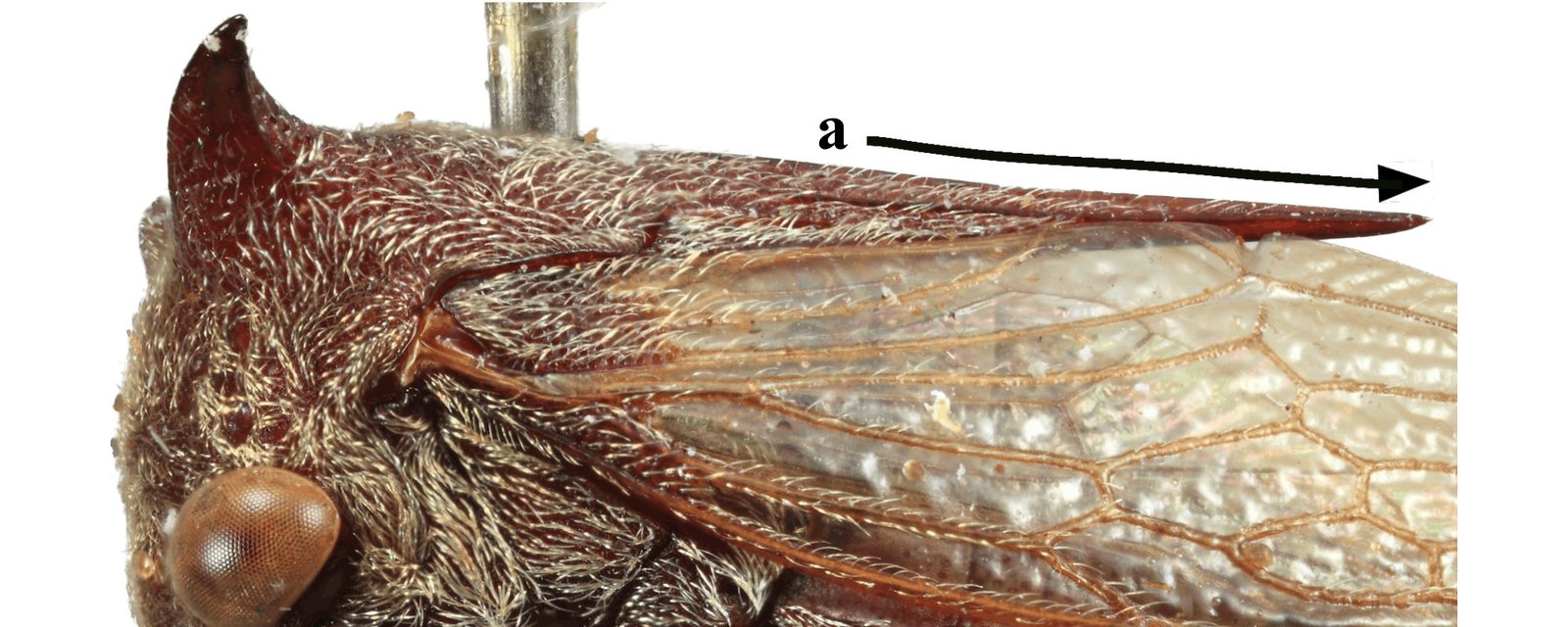		
–	Posterior process slightly thickened, gently sloping towards its apex (b)	***Tricentrus assamensis* Distant, 1908**
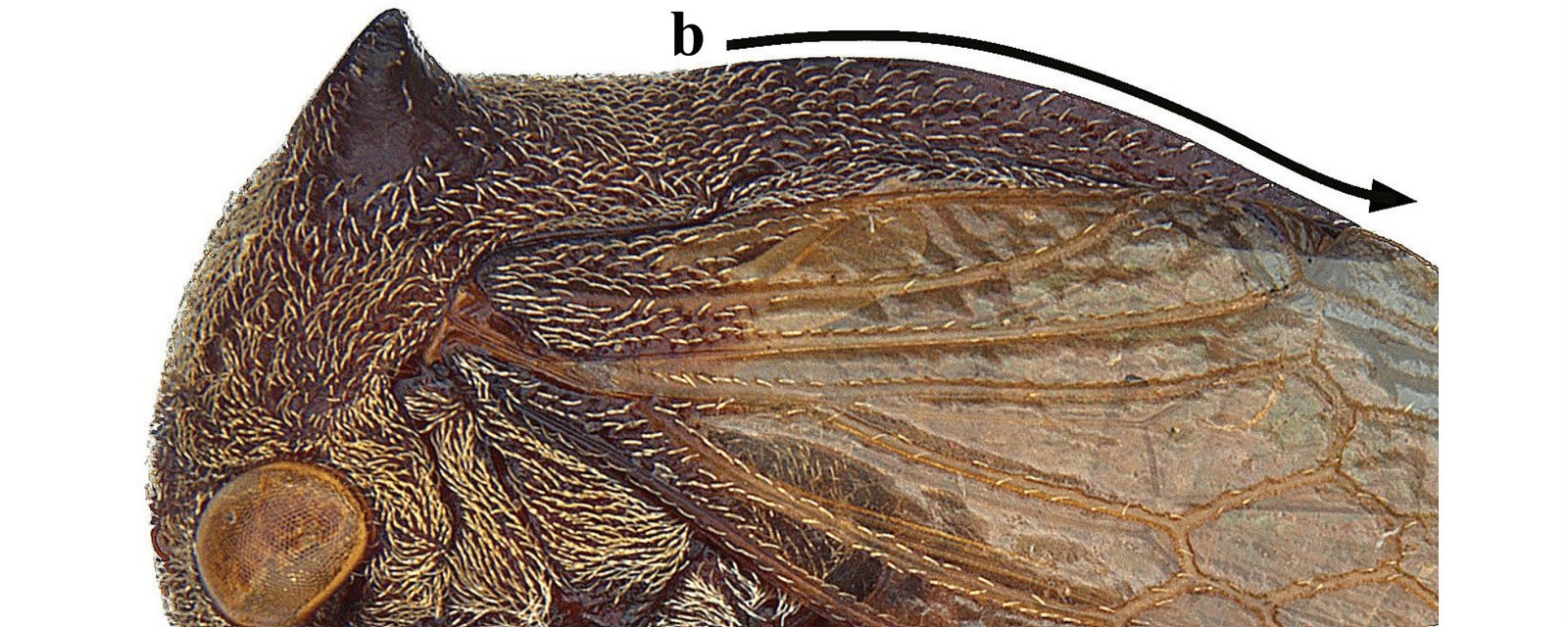		

## Discussion

This study provides the first comprehensive account of Singapore’s membracid diversity with 16 new species records (including eight morphospecies), raising the total number of recorded species from 36 to 52, spread across 11 tribes and 17 genera.

### Host plants and ant attendants

**Figure 7.**
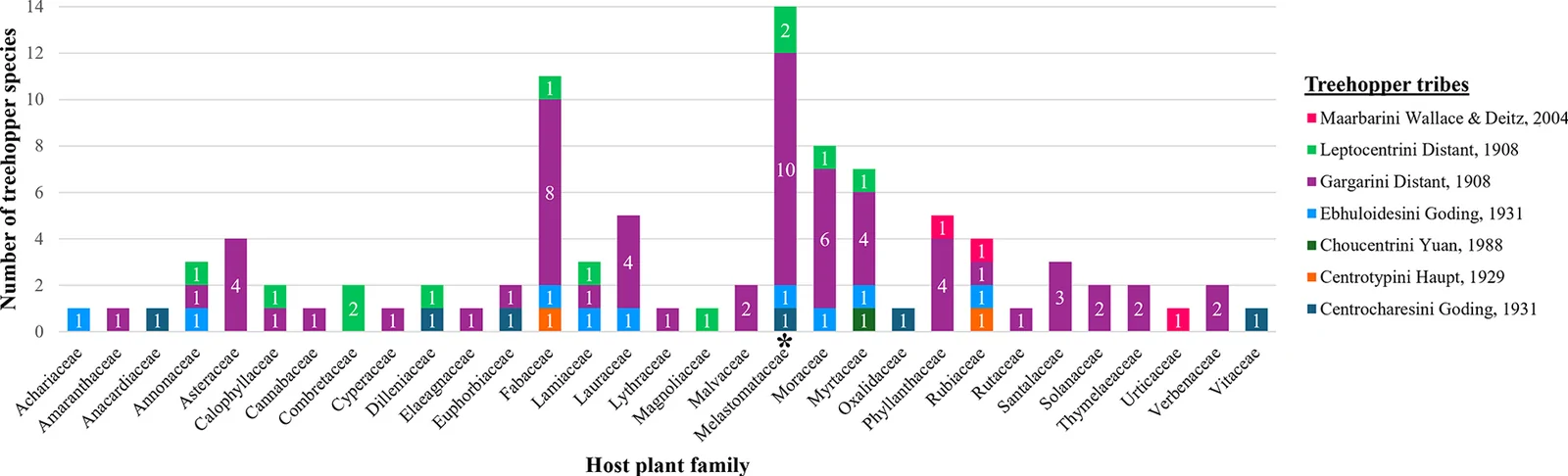
Singapore’s treehoppers sorted by their recorded host plant associations. Host records for *Leptocentrus taurus* are excluded from the figure on account of the species’ exceptionally polyphagous nature (concomitant with 72 plant species across 23 families; refer to the annotated species checklist for the full list of host plants of *L. taurus*). An asterisk (*) marks Melastomataceae which is represented almost entirely by records from *Melastoma malabathricum*. These patterns are based on opportunistic records and reflect only the species for which host plant data are available.

Host plant and ant attendant data were available for 37 and 11 treehopper species respectively. Notably, key contributions are highlighted for *Evanchon maculatum*, *Gargara luconica* and *Nilautama minutispina* which received their first host plant and ant attendant records. Such baseline ecological data remains absent for many Southeast Asian species. Host plant relationships are summarised in Fig. 7. Amongst the 37 species with host plant records, more than a third (14 spp. spread across Centrocharesini, Ebhuloidesini, Gargarini, and Leptocentrini) were found on *Melastoma malabathricum*, indicating the plant as a key host for Singapore’s treehoppers. Given that *M. malabathricum* is widespread across forest edges and disturbed habitats in Singapore, its strong concomitance with the local membracid fauna may reflect ecological availability rather than phylogenetic constraint. Broadly, other important host plant taxa include species from Fabaceae (collectively hosts 11 spp.), Moraceae (collectively hosts 8 spp.) and Myrtaceae (collectively hosts 7 spp.).

Species with host plant records were frequently polyphagous, with 22 species (across Centrocharesini, Ebhuloidesini, Gargarini, Leptocentrini, and Maarbarini) feeding on more than one plant family. Because these species use multiple hosts, their patterns scale upward to resemble broad host use at the tribal level, consistent with earlier observations that most centrotine tribes are polyphagous (Wallace and Deitz 2004). However, the patterns illustrated in Fig. 7 derive solely from Singaporean taxa (with *Leptocentrus taurus* excluded; see Fig. 7 caption) which represent only a small portion of each tribe’s overall diversity. As such, it remains unclear whether the apparent breadth of host use reflects characteristics of the tribes or simply the limited species sampled here. Expanded host plant surveys across a fuller representation of each tribe will help contextualise these patterns.

In contrast to host plant associations, ant attendance was documented for only 11 species. Amongst these, most were associated with Formicinae, particularly *Oecophylla smaragdina*. Ant attendance is known to decrease predation (Morales 2000) and in some instances may even be required for nymphal survival (Day 1999). Notably, Ananthasubramanian (1996) noted almost all Indian species to be ant attended. If similar patterns apply to species throughout the Old World, ant attendance of Singapore’s treehoppers is likely heavily undersampled.

### Taxonomic issues

While most Oriental taxa are easily distinguishable at the tribal and genus levels, species identification proved challenging without access to types as descriptions from earlier works (late 1800s to early 1900s) seldom provide useful diagnostic information. This issue is particularly pertinent in the hyper-diverse *Leptocentrus* (90 spp.), *Gargara* (174 spp.), and *Tricentrus* (238 spp.) which are incidentally the largest yet most poorly studied taxa within Membracidae. Species from some of the smaller genera such as *Centrochares* (7 spp.), *Centrotypus* (36 spp.), and *Leptobelus* (7 spp.) were also challenging to differentiate based on available descriptions (presented species counts derived from Dmitriev et al. 2022). While Goding’s keys (Goding 1931, 1934, 1939, 1949a, 1949b, 1950, 1951) attempted to differentiate all Oriental species known at the time, they are limited in usefulness due to usage of unclear terminology (Stegmann et al. 2002). To add further uncertainty, it is unlikely that Goding’s keys were based on types (Goding 1931). Species-level identification of Indian and Chinese membracids is somewhat better established given the descriptive work in the monographs of Ananthasubramanian (1996) and Yuan and Chou (2002), but even so, uncertainties remain. First, several species redescriptions provided by these workers were not based on types. Second, Yuan and Chou (2002) did not reference Ananthasubramanian (1996), and descriptions of the same species in both works occasionally differ (for example, see remarks in the annotated species checklist for *Leptocentrus longispinus*). A similar issue pertains to the independent treatment of the same fauna by Ananthasubramanian (India) and Ahmad and Yasmeen (Pakistan), who at times provided conflicting descriptions of Type specimens (for examples, see respective remarks in the annotated species checklist for *Tricentrus albomaculatus* and *T. gibbosulus*). Overall, species redescriptions based on type material are sorely needed to elucidate current ambiguities.

### Differentiating species based on external morphology

The present study relied upon external morphology to delineate species, and genitalia were not examined as prior workers have cited its lack of usefulness in separating centrotines (Ananthasubramanian 1987; Stegmann et al. 2002; Tishechkin 2022). Nonetheless, recent descriptive work has placed renewed emphasis on male genitalia in Centrotine taxonomy (Li et al. 2019, 2020, 2022a). An analysis centred upon external morphology in the present study was also necessary as most Oriental taxa were delineated by past workers using conspicuous external features. Such a reliance upon external morphology, however, may result in inaccurate species counts.

Membracids demonstrate intra-specific variation due to sexual dimorphism (including sexual dichromatism or multichromatism), host-plant specificity, or geographic isolation (Ananthasubramanian 1987; Wood 1993; Ananthasubramanian 1996; Stegmann et al. 2002; Yuan and Chou 2002; Su et al. 2014; De-la-Mora and Pinero 2023). As a result, past species counts based on morphological delineations may be inflated (Wood 1993). This issue is likely rampant in the hyper-diverse Oriental genera (*Leptocentrus*, *Gargara*, *Tricentrus*) as examination of the literature implies high degrees of similarity between congeners, often to the point where many are challenging to differentiate based on descriptions alone. On the other hand, underestimates of species diversity can also arise due to the insufficiency of morphological methods in identifying cryptic treehopper species complexes, the most well-known example being the *Enchenopa binotata* (Say, 1824) complex from the Neotropics: traditionally defined as a single species based on morphology, *E. binotata* is now recognised as a group of up to 15 species differentiated by substrate-borne mating signals and host plant specificity (Wood and Guttman 1982; McNett and Cocroft 2008; Hamilton and Cocroft 2009). Besides the *E. binotata* group, other complexes, including those of some Oriental taxa, have been similarly disentangled through integrative approaches (Su et al. 2014; Evangelista et al. 2017; Tishechkin 2022). A locally occurring species deemed as the foremost candidate for such investigations is *Leptocentrus taurus*. Besides its unusually wide distribution and exceptionally polyphagous nature, the species is also known to display slight levels of pronotal variation across wide series of specimens (Funkhouser 1915) and is currently in a state of taxonomic ambiguity given its contrasting sets of synonyms (see respective remarks in the annotated species checklist). Broadly, clarity of species limits within the hyper-diverse *Leptocentrus*, *Gargara*, and *Tricentrus* will likely benefit from integrative analyses as well.

Given the issues associated with differentiating species based on adult morphology, some workers have proposed the use of nymphal morphology as an alternate form of identification. Ananthasubramanian (1987) observed that nymphs of highly polymorphic species were remarkably monomorphic in appearance, displaying characters sufficient to distinguish them from related species. Works on neotropical membracids have also demonstrated the usefulness of nymphal characters in delineating genera (McKamey et al. 2015; McKamey and Wallner 2022). There are, however, challenges associated with nymph-based research. Most importantly, nymphal specimens are rarely present in natural history collections (Ananthasubramanian 1987). Furthermore, comparative data on the nymphal morphology of Oriental treehoppers is non-existent for most taxa. Hence, renewed collection efforts of nymphs alongside observations of their life history will be required before nymphal morphology becomes a useful tool for species identification.

## Conclusions

This study presents the first comprehensive account of Singapore’s treehoppers, noting a total of 52 locally occurring species (inclusive of 8 morphospecies) for which all available, pertinent information is compiled via an annotated species checklist. Further exploration of host plant / ant attendant relationships are highlighted. Crucially, the unidentifiable morphospecies within the present study highlight the need to clarify species identities via redescriptions based on types and integrative approaches. Given a paucity of work related to the membracids of Southeast Asia since the early 1900s, this study provides a timely update of our knowledge of the treehopper fauna of this region, particularly for Singapore. With approximately 0.28% of Singapore’s original vegetative cover intact (Yee et al. 2011), it is possible that some species originally recorded during the 1800s and 1900s have become locally extinct. Future targeted surveys, particularly within primary forest habitats, will be helpful to ascertain the continued presence of previously recorded taxa. The uncovering of new records for Singapore suggests that treehopper diversity is also undersampled in adjacent Southeast Asian states.

## Additional information

### Artificial Intelligence (AI) use

The authors accept full responsibility for the content of the manuscript, including the disclosure of any use of AI.

No AI tools were used in the preparation of this manuscript.

### Author ORCIDs

V. Tan https://orcid.org/0009-0002-8530-7935

W.S. Hwang https://orcid.org/0000-0003-3462-2142

### Data availability

All of the data that support the findings of this study are available in the main text or Supplementary Information.

## Supplementary materials

Supplementary material 1 A simplified checklist, summaries of host plant and ant attendant data Data type docx https://binary.pensoft.net/file/1816681This dataset is made available under the Open Database License (http://opendatacommons.org/licenses/odbl/1.0/). The Open Database License (ODbL) is a license agreement intended to allow users to freely share, modify, and use this Dataset while maintaining this same freedom for others, provided that the original source and author(s) are credited. Varen Tan, Wei Song Hwang
