# Supplementary material for: The treehoppers (Hemiptera, Membracidae) of Singapore: An annotated checklist, new records, and an illustrated identification key
Source: Zookeys. 2026 Oct 23;1295:161–225. doi: 10.3897/zookeys.1295.204613 (PMC13723010; doi:10.3897/zookeys.1295.204613)
Supplement: Supplementary file 1 [file zookeys-1295-161_article-204613__-s001.docx]

**Supplementary data**

**Table S1.** Singapore’s treehoppers sorted by their recorded host plant associations. Records are derived from literature, museum labels, and personal observation. To provide a clearer view of host plant relationships, host records for *Leptocentrus taurus* are excluded from the table on account of the species’ exceptionally polyphagous nature (concomitant with 72 plant species across 23 families); refer to the annotated checklist for the full list of host plants of *L*. *taurus*.

| **Host family** | **Host species** | **Treehopper species** |
| --- | --- | --- |
| Achariaceae | *Hydnocarpus alpinus* | *Ebhul varius* (Yuan and Chou 2002) |
| Amaranthaceae | *Amaranthus viridis* | *Tricentrus gibbosulus* (Ahmad and Yasmeen 1978) |
| Anacardiaceae | *Anacardium* sp. | *Centrochares ridleyana* |
| Annonaceae | *Annona muricata* | *Nilautama minutispina* |
|  | *Cananga odorata* | *Ebhul varius* (Yuan and Chou 2002), *Tricentrus albomaculatus* (Zimmerman 1948) |
| Asteraceae | *Cyanthillium cinereum* | *Tricentrus congestus* (Ananthasubramanian and Ananthakrishnan 1975) |
|  | *Mikania scandens* | *Tricentrus gibbosulus* (Ahmad and Yasmeen 1978) |
|  | *Mikania* sp. | *Tricentrus* cf. *albomaculatus* |
|  | *Xanthium strumarium* | *Tricentrus albomaculatus* (Ahmad and Yasmeen 1978), *Tricentrus gibbosulus* (Ahmad and Yasmeen 1978) |
|  | *Xanthium* sp. | *Tricentrus gibbosulus* (Ananthasubramanian 1996) |
| Calophyllaceae | *Calophyllum inophyllum* | *Gargara* sp. 2, *Nilautama minutispina* |
| Cannabaceae | *Humulus scandens* | *Tricentrus basalis* (Yuan and Chou 2002) |
| Combretaceae | *Terminalia catappa* | *Leptocentrus leucaspis* (Ananthasubramanian 1996), *Nilautama minutispina* |
|  | *Terminalia* sp. | *Nilautama minutispina* |
| Cyperaceae | *Hypolytrum nemorum* | *Gargara nitidipennis* |
|  | *Scleria* sp. | *Gargara nitidipennis* |
| Dilleniaceae | *Dillenia excelsa* | *Centrochares ridleyana* |
|  | *Dillenia suffruticosa* | *Nilautama minutispina* |
| Elaeagnaceae | *Elaeagnus pungens* | *Tricentrus brunneus* (Yuan and Chou 2002) |
| Euphorbiaceae | *Acalypha amentacea* var. *amentacea* | *Centrochares horrifica*, *Sipylus dilatatus* (Funkhouser 1919) |
| Fabaceae | *Acacia* sp. | *Tricentrus* cf. *albomaculatus* |
|  | *Archidendron* sp. | *Centrotypus belus* |
|  | *Bauhinia tomentosa* | *Leptocentrus leucaspis* (Ananthasubramanian 1996) |
|  | *Bauhinia* sp. | *Tricentrus acuticornis* |
|  | *Butea monosperma* | *Gargara rubrogranulata* (Funkhouser 1935a) |
|  | *Cajanus cajan* | *Tricentrus albomaculatus* (Zimmerman 1948) |
|  | *Dalbergia latifolia* | *Gargara sordida* (Funkhouser 1935a) |
|  | *Dalbergia sissoo* | *Tricentrus albomaculatus* (Ahmad and Yasmeen 1978) |
|  | *Dendrolobium umbellatum* | *Gargara luconica*, *Gargara triangulata*, *Tricentrus acuticornis* |
|  | *Parkia* sp. | *Tricentrus* cf. *albomaculatus* |
|  | *Pongamia pinnata* | *Leptocentrus leucaspis* (Ananthasubramanian 1996) |
|  | *Robinia pseudoacacia* | *Tricentrus brunneus* (Yuan and Chou 2002) |
|  | *Senna bicapsularis* | *Tricentrus albomaculatus* (Zimmerman 1948) |
|  | *Sesbania* sp. | *Tricentrus albomaculatus* (Zimmerman 1948) |
|  | *Tamarindus indica* | *Ebhul varius* (Yuan and Chou 2002) |
| Lamiaceae | *Vitex pinnata* | *Ebhul varius*, *Gargara triangulata* (Funkhouser 1935a) |
|  | *Vitex* sp. | *Nilautama minutispina* |
| Lauraceae | *Cinnamomum burmanni* | *Ebhul varius* (Funkhouser 1927a; Yuan and Chou 2002) |
|  | *Cinnamomum iners* | *Gargara projecta*, *Gargara* sp. 2, *Tricentrus assamensis* |
|  | *Cinnamomum* sp*.* | *Gargara nervosa* |
| Lythraceae | *Sonneratia ovata* | *Gargara luconica* |
| Magnoliaceae | *Magnolia champaca* | *Leptocentrus longispinus* (Ananthasubramanian 1996) |
| Malvaceae | *Ayenia maingayi* | *Gargara sordida* |
|  | *Hibiscus tiliaceus* | *Tricentrus spinis* |
| Melastomataceae | *Melastoma malabathricum* | *Centrochares ridleyana* (Cheong 2011), *Ebhul varius*, *Gargara luconica*, *Gargara nitidipennis*, *Gargara projecta*, *Gargara rubrogranulata*, *Leptocentrus* sp., *Nilautama minutispina*, *Sipylus* sp., *Tricentrus acuticornis*, *Tricentrus assamensis*, *Tricentrus banguensis*, *Tricentrus intermedius*, *Tricentrus spinis* |
|  | *Memecylon caeruleum* | *Gargara rubrogranulata* |
|  | *Memecylon* sp. | *Gargara rubrogranulata* |
| Moraceae | *Broussonetia papyrifera* | *Tricentrus brunneus* (Yuan and Chou 2002) |
|  | *Ficus ampelos* | *Ebhul varius* (Funkhouser 1927a) |
|  | *Ficus benjamina* | *Tricentrus fulgidus* (Yuan and Chou 2002) |
|  | *Ficus heteromorpha* | *Tricentrus spinicornis* (Yuan and Chou 2002) |
|  | *Ficus hispida* | *Tricentrus fulgidus* (Yuan and Chou 2002) |
|  | *Ficus lacor* | *Tricentrus fulgidus* (Yuan and Chou 2002) |
|  | *Ficus microcarpa* | *Ebhul varius* (Yuan and Chou 2002) |
|  | *Ficus religiosa* | *Tricentrus fulgidus* (Yuan and Chou 2002) |
|  | *Ficus* sp. | *Gargara rubrogranulata, Nilautama minutispina, Tricentrus assamensis, Tricentrus fulgidus* |
|  | *Streblus asper* | *Sipylus dilatatus* |
|  | *Streblus elongatus* | *Nilautama minutispina* |
| Myrtaceae | *Eucalyptus robusta* | *Tricentrus albomaculatus* (Yuan and Chou 2002) |
|  | *Eucalyptus* sp. | *Tricentrus albomaculatus* (Kershaw 1913; Zimmerman 1948) |
|  | *Rhodomyrtus tomentosa* | *Gargara luconica* |
|  | *Syzygium grande* | *Ebhul varius*, *Evanchon maculatum*, *Gargara projecta*, *Gargara rubrogranulata*, *Nilautama minutispina* |
|  | *Syzygium* sp. | *Ebhul varius*, *Evanchon maculatum*, *Gargara rubrogranulata* |
| Oxalidaceae | *Averrhoa carambola* | *Centrochares ridleyana* |
| Phyllanthaceae | *Antidesma bunius* | *Gargara luconica*, *Telingana recurvata* |
|  | *Bridelia tomentosa* | *Gargara luconica*, *Gargara sordida*, *Tricentrus banguensis* |
|  | *Phyllanthus reticulatus* | *Sipylus dilatatus* (Funkhouser 1919) |
| Rubiaceae | *Uncaria rhynchophylla* | *Ebhul varius* (Yuan and Chou 2002) |
|  | *Uncaria* sp. | *Centrotypus* sp. 1, *Gargara* sp. 1, *Telingana recurvata* |
| Rutaceae | *Citrus* sp. | *Gargara rubrogranulata* |
| Santalaceae | *Santalum album* | *Cryptaspidia piceola* (Funkhouser 1933), *Tricentrus assamensis* (Funkhouser 1933; Ananthasubramanian 1996), *Tricentrus gibbosulus* (Dover 1932) |
| Solanaceae | *Datura metel* | *Tricentrus albomaculatus* (Ananthasubramanian 1996) |
|  | *Withania somnifera* | *Tricentrus albomaculatus*, *Tricentrus gibbosulus* (Ahmad and Yasmeen 1978) |
| Thymelaeaceae | *Aquilaria malaccensis* | *Gargara projecta* |
|  | *Phaleria macrocarpa* | *Gargara luconica* |
| Urticaceae | *Poikilospermum suaveolens* | *Telingana recurvata* |
| Verbenaceae | *Phyla nodiflora* | *Tricentrus albomaculatus* (Ahmad and Yasmeen 1978), *Tricentrus gibbosulus* (Ahmad and Yasmeen 1978) |
| Vitaceae | *Leea indica* | *Centrochares ridleyana* |

**Table S2.** Singapore’s treehoppers sorted by their recorded ant attendants. Records are derived from literature, museum labels, and personal observation.

| **Ant subfamily** | **Ant species** | **Treehopper species** |
| --- | --- | --- |
| Dolichoderinae | *Dolichoderus thoracicus* | *Ebhul varius* (Funkhouser 1927a) |
|  | *Dolichoderus* sp. | *Evanchon maculatum* |
| Formicinae | *Anoplolepis gracilipes* | *Gargara luconica*, *Gargara nitidipennis* |
|  | *Camponotus compressus* | *Leptocentrus taurus* (Ananthasubramanian 1996) |
|  | *Oecophylla smaragdina* | *Centrochares ridleyana*, *Gargara* sp. 2, *Tricentrus assamensis*, *Leptocentrus taurus* (Ananthasubramanian 1996), *Nilautama minutispina* |
| Myrmicinae | *Pheidole megacephala* | *Tricentrus albomaculatus* (Kershaw 1913) |
|  | *Solenopsis geminata* | *Leptocentrus leucaspis* (Ananthasubramanian 1996) |
